# Pyruvate kinase M2 regulates mitochondrial homeostasis in cisplatin-induced acute kidney injury

**DOI:** 10.1038/s41419-023-06195-z

**Published:** 2023-10-10

**Authors:** Wenjia Xie, Qingyun He, Yan Zhang, Xinxin Xu, Ping Wen, Hongdi Cao, Yang Zhou, Jing Luo, Junwei Yang, Lei Jiang

**Affiliations:** Center for Kidney Disease, The Second Affiliated Hospital, Nanjing Medical University, Nanjing, China

**Keywords:** Apoptosis, Acute kidney injury

## Abstract

An important pathophysiological process of acute kidney injury (AKI) is mitochondrial fragmentation in renal tubular epithelial cells, which leads to cell death. Pyruvate kinase M2 (PKM2) is an active protein with various biological functions that participates in regulating glycolysis and plays a key role in regulating cell survival. However, the role and mechanism of PKM2 in regulating cell survival during AKI remain unclear. Here, we found that the phosphorylation of PKM2 contributed to the formation of the PKM2 dimer and translocation of PKM2 into the mitochondria after treatment with staurosporine or cisplatin. Mitochondrial PKM2 binds myosin heavy chain 9 (MYH9) to promote dynamin-related protein 1 (DRP1)-mediated mitochondrial fragmentation. Both in vivo and in vitro, PKM2-specific loss or regulation PKM2 activity partially limits mitochondrial fragmentation, alleviating renal tubular injury and cell death, including apoptosis, necroptosis, and ferroptosis. Moreover, staurosporine or cisplatin-induced mitochondrial fragmentation and cell death were reversed in cultured cells by inhibiting MYH9 activity. Taken together, our results indicate that the regulation of PKM2 abundance and activity to inhibit mitochondrial translocation may maintain mitochondrial integrity and provide a new therapeutic strategy for treating AKI.

## Introduction

Acute kidney injury (AKI) is a clinically common, severe disease with high morbidity and mortality rates in both developing and developed countries [1]. According to statistics, the incidence rate of AKI in hospitalized patients in China ranges from 3.02% [2] to 11.6% [3], which accounts for approximately 2 million lives worldwide each year [4]. Since cisplatin is a commonly used chemotherapeutic drug in clinical patients with tumor, cisplatin-induced AKI might be an important medical problem [5]. For decades, scientists have continued to study the pathophysiological mechanism, drug intervention and clinical trials of cisplatin-induced AKI. In fact, there is no clear intervention measures to effectively limit or reverse cisplatin-induced AKI. Most of the previous research results are still limited to the experimental research stage and have not been successfully translated into clinical trials. Therefore, it is necessary to re-evaluate the research strategies for preventing and treating cisplatin-induced AKI and further explore the pathophysiological mechanism of AKI to improve the clinical relevance of experimental studies.

Renal tubular epithelial cell injury and death are considered as the main characteristics of cisplatin-induced AKI [6]. Our previous studies have revealed that antagonizing apoptosis could alleviate kidney injury induced by cisplatin [7, 8]. Moreover, specific knockdown of receptor interacting protein (RIP) and mixed lineage kinase domain like pseudokinase (MLKL) inhibited cisplatin-induced necroptosis and could prevent renal tubular injury [9]. Ferrostatin-1, an ferroptosis inhibitor, was shown to alleviate cisplatin-induced AKI [10]. This suggests that cisplatin-induced death of renal tubular epithelial cells is complex and multiform. Therefore, it is necessary to study the mechanism of upstream regulation and seek therapeutic targets. Central to tubular injury is mitochondrial homeostasis imbalance [11]. Proximal renal tubular epithelial cells are rich in mitochondria and rely on mitochondrial respiration to maintain renal tubular reabsorption, secretion and other functions [12]. Mitochondria are the main target for cisplatin to damage proximal tubular epithelial cells. Cisplatin could induce reactive oxygen species (ROS) generation, destroy the integrity of respiratory chain complexes, interfere with mitochondrial fission/fusion to promote mitochondrial fragmentation [8, 13]. Thus, an in-depth understanding of the molecular basis of mitochondria-regulating cell survival may provide new research ideas for elucidating the pathogenesis and treatment strategies of AKI.

Pyruvate kinase M2 (PKM2), an M2 subtype encoded by the PK gene, is an important regulatory protein involved in glucose catabolism. It catalyzes the transfer of phosphate groups from phosphoenolpyruvate (PEP) to adenosine diphosphate (ADP), producing pyruvate and adenosine triphosphate (ATP) to regulate the final rate-limiting step of glycolysis [14–16]. In addition to its metabolic role in glycolysis, the function of PKM2 in regulating cell survival has been recognized and has focused on diseases, such as tumors, inflammation, and cerebrovascular diseases [17–20]. The role of PKM2 has been wildly accepted in podocytes. PKM2 activation protects against podocyte injury and the progression of diabetic kidney diseases and hypertensive nephropathy, and PKM2 knockout in podocytes aggravated the podocyte injury [21–23]. In recent years, increasing attention has been paid to the role of PKM2 in AKI. PKM2 expression in renal tubular epithelial cells was found to increase [24], and the level of PKM2 in urine was used as a sensitive biomarker at the early stage of cisplatin-induced AKI [25]. *Pkm2* knockout in tubular epithelial cells protect kidneys against ischemia–reperfusion (I/R)-induced AKI [26]. In addition, Wu et al. [27] confirmed that inhibiting PKM2 by Shikonin alleviated lipopolysaccharide (LPS)-induced AKI. While, the mechanisms of PKM2 in AKI are still unknown.

The biological effects of PKM2 are related to its aggregation status and subcellular localization. Although both the tetramer and dimer of PKM2 are composed of the same monomer, their biological effects are quite different [28, 29]. Tyrosine 105 on PKM2 (PKM2 Tyr^105^) can be directly phosphorylated, which inhibits the presence of the PKM2 tetramer to reduce its pyruvate kinase activity and promote the PKM2 dimer formation to increase its protein kinase activity [30]. PKM2 dimers exist in different intracellular locations to perform their biological functions [31]. Many studies have suggested that mitochondrial PKM2 stabilizes mitochondrial biosynthesis, regulates fission/fusion [21, 32], and stabilizes B-cell lymphoma 2 (Bcl2) [33] or voltage-dependent anionic channel 3 (VDAC3) [34] to promote cell survival. Similarly, PKM2 translocation into the nucleus induces non-caspase-dependent cell death [35]. An in-depth exploration of the subcellular PKM2 localization may be the basis for its specific biological effects.

In this study, we used tubular epithelial cells specific *Pkm2* knockout mice, and Shikonin or TEPP46 supplementation to investigate the role and mechanism of PKM2 in tubular epithelial cells during AKI. This study not only identifies the physiological function of PKM2 in the survival of tubular epithelial cells, but also offers potential therapeutic targets to preserve kidney function and prevent AKI.

## Results

### Mitochondrial PKM2 is increased in tubular epithelial cells during cisplatin-induced AKI

**Fig. 1:**
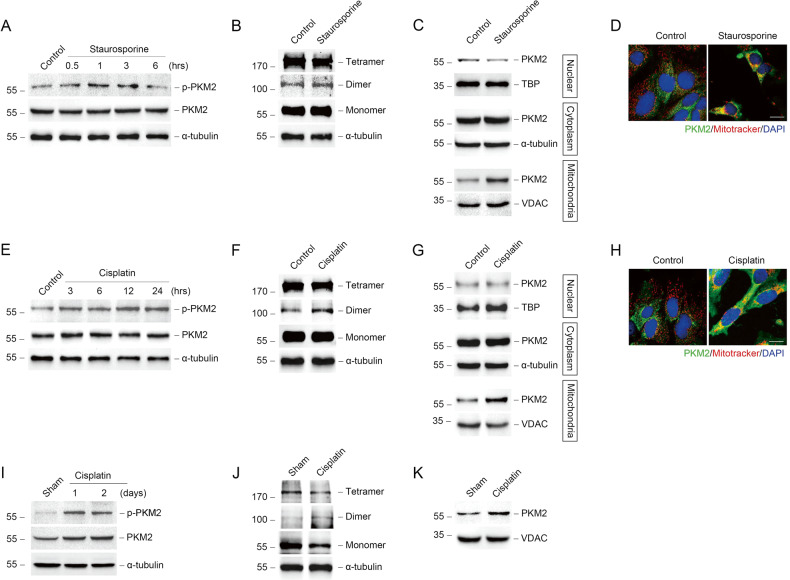
Mitochondrial PKM2 is increased in tubular epithelial cells during AKI. **A** Western blots showing the abundance of p-PKM2 and PKM2 in NRK-52E cells after staurosporine treatment at different times. **B** Representative images of cross-linking NRK-52E cells treated with staurosporine for 1 hour to show PKM2 monomer, dimer, and tetramer. **C** Western blots of PKM2 abundance in the nucleus, cytoplasm, and mitochondria of NRK-52E cells. **D** Representative images showing the mitochondrial morphology visualized by staining of PKM2 and mitotracker red in primary tubular epithelial cells (PTCs) treated with staurosporine or cisplatin. Mitotracker, red; PKM2, green; DAPI, blue. scale bar = 20 μm. **E** Western blots showing the abundance of p-PKM2 and PKM2 in NRK-52E cells after cisplatin treatment at different times. **F** Representative images of cross-linking NRK-52E cells treated with cisplatin for 12 hours to show PKM2 monomer, dimer, and tetramer. **G** Western blots of PKM2 abundance in the nucleus, cytoplasm, and mitochondria of NRK-52E cells after cisplatin treatment for 12 hours. **H** Representative images showing the mitochondrial morphology visualized by staining of PKM2 and mitotracker red in PTCs treated with cisplatin. scale bar = 20 μm. **I** Western blot analysis of p-PKM2 and PKM2 expression in kidney cortex after cisplatin injection for different times. **J** Western blots of cross-linking renal cortexes from mice 1 day after cisplatin treatment to show PKM2 monomer, dimer, and tetramer. **K** Western blots of the abundance of PKM2 in mitochondria isolated from renal cortexes from cisplatin-induced mice at day 1.

We first examined the expression of PKM2 in tubular epithelial cells during AKI. We used staurosporine or cisplatin to stimulate normal rat kidney epithelial (NRK-52E) cells to induce acute injury in vitro. As shown in Fig. 1A, E, PKM2 phosphorylation (p-PKM2) was substantially increased after staurosporine or cisplatin stimulation. Some studies have shown that PKM2 phosphorylation promotes PKM2 dimer formation [36]. As expected, PKM2 was transformed from tetramer to dimer in NRK-52E cells after staurosporine or cisplatin stimulation (Fig. 1B, F). Dimer PKM2 has different subcellular locations including nucleus, cytoplasm, and mitochondria [31]. First, in order to clarify the subcellular localization of PKM2, the nucleus, cytoplasm, and mitochondria were isolated from NRK-52E cells. In contrast to our previous results of transforming growth factor, beta 1 (TGF-β1) stimulation, PKM2 expression in the nucleus did not increase in response to staurosporine or cisplatin stimulation, and seemed to decrease slightly. However, PKM2 expression in the mitochondria was significantly increased (Fig. 1C, D, G, H).

Furthermore, we analyzed the PKM2 expression in vivo. The expression of p-PKM2 was increased in kidney tissues at day 1 and day 2 after cisplatin injection (Fig. 1I). Consistent with in vitro study, dimer PKM2 was found in cisplatin injured kidneys at day 1 (Fig. 1J). The abundance of PKM2 and dimer PKM2 was also increased in mitochondria (Fig. 1K and Supplementary Fig. 1). Those data indicated that during the progressing of AKI induced by cisplatin, PKM2 was translocated to mitochondria.

### Mitochondrial PKM2 binds to myosin heavy chain 9 (MYH9) to promote dynamin-related protein 1 (DRP1)-mediated mitochondrial fragmentation in response to acute injury

**Fig. 2:**
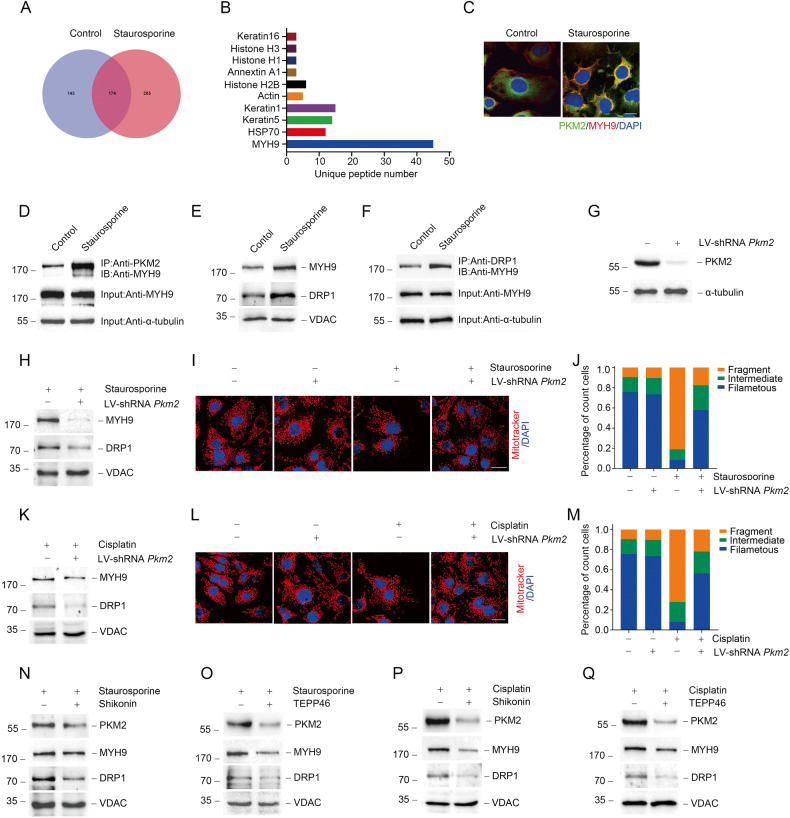
Mitochondrial PKM2 binds MYH9 to promote DRP1-mediated mitochondrial fragmentation in response to acute injury in vivo. **A**, **B** Co-IP of PKM2 and further proteomic analysis by LC-MS/MS in NRK-52E cells treated 1 h with staurosporine. **C** Representative images showing the confocal immunofluorescence staining of PKM2 and MYH9 in PTCs. PKM2, green; MYH9, red; DAPI, blue. scale bar=20 μm. **D** Co-IP experiments detected the interaction of PKM2 and MYH9 in NRK-52E cells after staurosporine stimulation. **E** Western blot results of MYH9 and DRP1 expression in mitochondria extracted from NRK-52E cells after staurosporine treatment. **F** Co-IP experiments detected the interaction of MYH9 and DRP1 in NRK-52E cells after staurosporine treatment. **G** The expression of PKM2 protein in NRK-52E cells with or without lentivirus shRNA *Pkm2* (LV-shRNA *Pkm2*) transfection. **H**, **K** Western blot results of MYH9 and DRP1 expression in mitochondria separated from staurosporine or cisplatin treated NRK-52E cells transfected with or without LV-shRNA *Pkm2*. **I**, **J**, **L**, **M** Representative images of confocal immunofluorescence and percentage of mitochondrial fragmentation in PTCs. Mitotracker, red; DAPI, blue. scale bar = 20 μm. **N**, **O** Western blot analysis of MYH9 and DRP1 expression in mitochondria from staurosporine-treated NRK-52E cells pretreated with Shikonin or TEPP46. **P**, **Q** Western blot analysis of MYH9 and DRP1 expression in mitochondria from cisplatin-treated NRK-52E cells pretreated with Shikonin or TEPP46.

To explore the role of PKM2 in the mitochondria, we conducted a co-immunoprecipitation (co-IP) experiment on PKM2 binding proteins in NRK-52E cells treated with staurosporine. Proteomic analysis using liquid chromatography-mass spectrometry/mass spectrometry (LC-MS/MS) showed that MYH9 was the most abundant protein bound to PKM2 (Fig. 2A, B). Simultaneously, colocalization of PKM2 and MYH9 was observed by confocal immunofluorescence in NRK-52E cells treated with staurosporine (Fig. 2C). Furthermore, western blotting results of co-IP revealed that PKM2 bound to MYH9 after staurosporine treatment (Fig. 2D). Previous studies have found that MYH9 may mediate mitochondrial fission through DRP1 [37, 38]. Our results indicated that under staurosporine stimulation, MYH9 and DRP1 were both increased in mitochondria (Fig. 2E). Moreover, an interaction between MYH9 and DRP1, which was significantly enhanced by staurosporine, was detected in NRK-52E cells by co-IP (Fig. 2F). Knockdown *Myh9* expression by transfected with siRNA or inhibiting MYH9 activity by Blebbistatin could decreased the phosphorylated DRP1 level in NRK-52E cells treated with staurosporine or cisplatin (Supplementary Fig. 2 and Supplementary Fig. 3). Furthermore, staurosporine or cisplatin-induced mitochondrial fragmentation and cell death were limited by MYH9 inhibition (Supplementary Fig. 2 and Supplementary Fig. 3).

We next investigated the role of PKM2 in regulating MYH9 and mitochondrial dynamic changes. First, we transfected NRK-52E cells with lentivirus mediated *Pkm2* shRNA (LV-shRNA *Pkm2*) to downregulate PKM2 expression (Fig. 2G). The expression of p-DRP1, and mitochondrial MYH9 and DRP1 were reduced in NRK-52E cells transfected with *Pkm2* shRNA under staurosporine or cisplatin treatment (Fig. 2H, K; Supplementary Fig. 4A, D). Parallel to this, PKM2 expression downregulation inhibited staurosporine or cisplatin-induced mitochondrial fragmentation (Fig. 2I, J, L, M).

To explore the possible role of PKM2 activity in AKI, Shikonin, and TEPP46 were used to regulate the activity of PKM2. Shikonin is a naphthoquinone compound extracted from the roots of Chinese traditional medicine and has been identified as a new PKM2 inhibitor that prevents glycolysis in cancer cells [39]. Additionally, TEPP46 was used to inhibit PKM2 dimer formation to reduce its activity as a protein kinase [31]. We next used Shikonin and TEPP46 to pre-treat NRK-52E cells to explore the role of PKM2 during acute injury induced by staurosporine or cisplatin. As shown in Fig. 2N–Q, both Shikonin and TEPP46 could hamper the increasement of p-DRP1, and mitochondrial MYH9 and DRP1 expression induced by staurosporine or cisplatin (Fig. 2N–Q, Supplementary Fig. 4B, C, 4E, F). Besides, the mitochondrial fragmentation was also improved by Shikonin or TEPP46 treatment (Supplementary Fig. 5). The above results suggest that mitochondrial PKM2 binds to MYH9 and promotes DRP1-mediated mitochondrial fragmentation during acute injury.

### PKM2 regulates renal tubular cell death in vitro

**Fig. 3:**
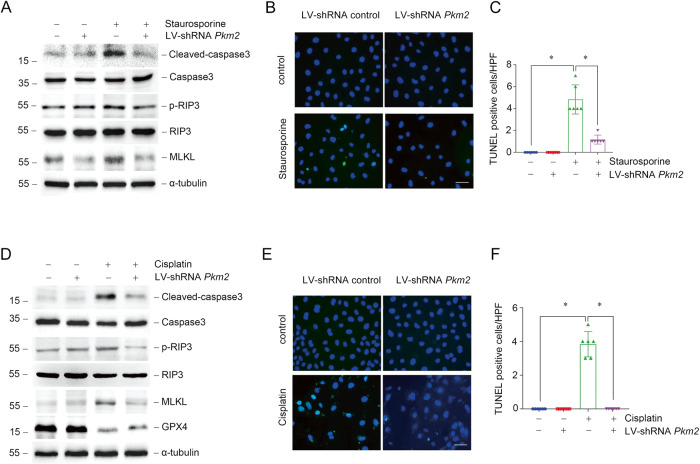
Down-regulation of PKM2 expression alleviates renal tubular cell death in vitro. **A** Western blot analysis of cleaved-caspase3, caspase3, p-RIP3, RIP3, and MLKL expression in NRK-52E cells treated with staurosporine with or without LV-shRNA *Pkm2* transfection. **B**, **C** Representative micrographs and quantification results of TUNEL staining as indicated in NRK-52E cells. scale bar = 50 μm. *P < 0.05, *n* = 6 per group. **D** Western blot analysis of cleaved-caspase3, caspase3, p-RIP3, RIP3, MLKL, and GPX4 expression in NRK-52E cells treated with cisplatin with or without LV-shRNA *Pkm2* transfection. **E**, **F** Representative micrographs and quantification results of TUNEL staining as indicated in NRK-52E cells. scale bar = 50 μm. *P < 0.05, *n* = 6 per group.

Renal tubular injury and cell death are predisposing factors; however, they are also AKI outcomes. We found that the expression of cleaved-caspase3 was lower in *Pkm2* knockdown group compared to scramble shRNA group for both staurosporine treatment, indicating that knowndown *Pkm2* expression could inhibit apoptosis induced by staurosporine (Fig. 3A). Besides apoptosis, we also found necroptosis related proteins such as phosphorylated RIP3 and MLKL were both upregulated in NRK-52E cells under staurosporine treatment, which was consistent with previous funding [40]. *Pkm2* knockdown could also block the upregulation of phosphorylated RIP3 and MLKL induced by staurosporine (Fig. 3A). TUNEL staining analysis showed that cell death induced by staurosporine could regulated by PKM2 (Fig. 3B, C). The pro-apoptotic effect of PKM2 was also confirmed in cisplatin-treated NRK-52E cells shown as lower cleaved-caspase3, p-RIP3, and MLKL expression, less TUNEL positive cells after *Pkm2* knockdown (Fig. 3D–F). Surprisingly, we found that the abundance of glutathione peroxidase 4 (GPX4), which regulates ferroptosis, was reduced after cisplatin stimulation, while *Pkm2* knockdown restored its expression (Fig. 3D).

**Fig. 4:**
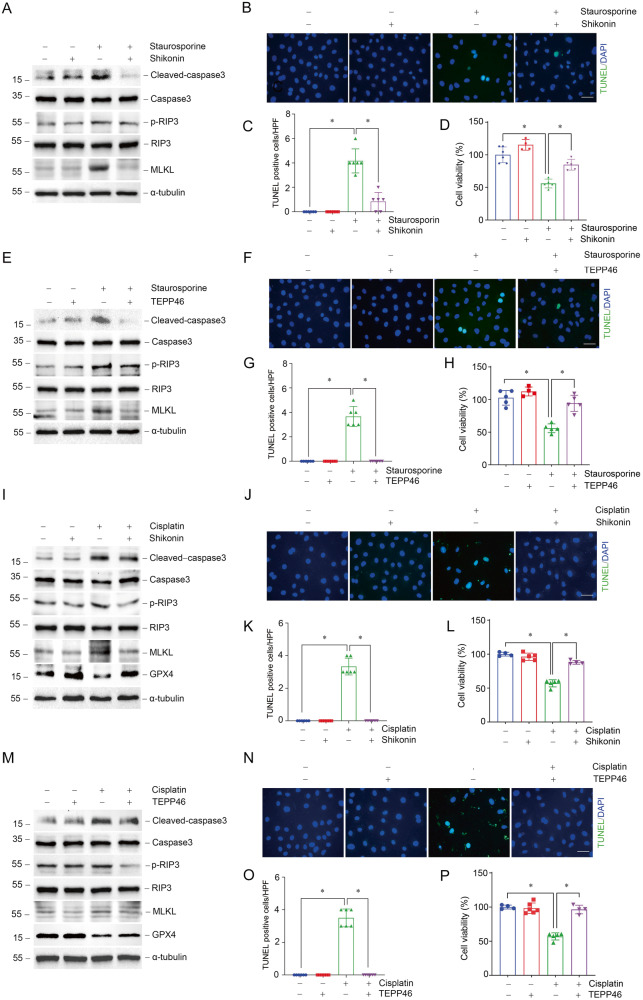
Regulation of PKM2 activity attenuates cell death in NRK-52E cells induced by staurosporine or cisplatin. **A**, **D** Western blots of cleaved-caspase3, caspase3, p-RIP3, RIP3, and MLKL expression in NRK-52E cells stimulated by staurosporine pre-treated with Shikonin or TEPP46. **B**, **C**, **F**, **G** Representative micrographs and quantification results of TUNEL staining as indicated in NRK-52E cells. scale bar=50 μm. *P < 0.05, *n* = 6 per group. **D**, **H** CCK-8 experiments of cell viability in NRK-52E cells treated with staurosporine with Shikonin or TEPP46 pre-treatment. *P <0.05, n = 4 ~ 5 per group. **I**, **M** Western blots of cleaved-caspase3, caspase3, p-RIP3, RIP3, MLKL, and GPX4 expression in NRK-52E cells stimulated by cisplatin pre-treated with Shikonin or TEPP46. **J**, **K**, **N**, **O** Representative micrographs and quantification results of TUNEL staining as indicated in NRK-52E cells. scale bar = 50 μm. *P < 0.05, *n* = 6 per group. **L**, **P** CCK-8 experiments of cell viability in cisplatin-treated NRK-52E cells with Shikonin or TEPP46 pre-treatment. *P < 0.05, *n* = 4–5 per group.

Subsequently, we analyzed whether regulating PKM2 activity would be beneficial for the survival of tubular epithelial cells after staurosporine or cisplatin treatment. Both Shikonin and TEPP46 render NRK-52E cells resistant to apoptosis and necroptosis induced by staurosporine (Fig. 4A–H). Additionally, cisplatin induced apoptosis, necroptosis and ferroptosis could also be hampered by Shikonin or TEPP46 (Fig. 4I–P). In summary, reduced PKM2 expression or inhibition of PKM2 phosphorylation or dimerization could promote cell survival in the presence of staurosporine or cisplatin stimulation in vitro.

### The role of PKM2 in AKI induced by cisplatin in vivo

**Fig. 5:**
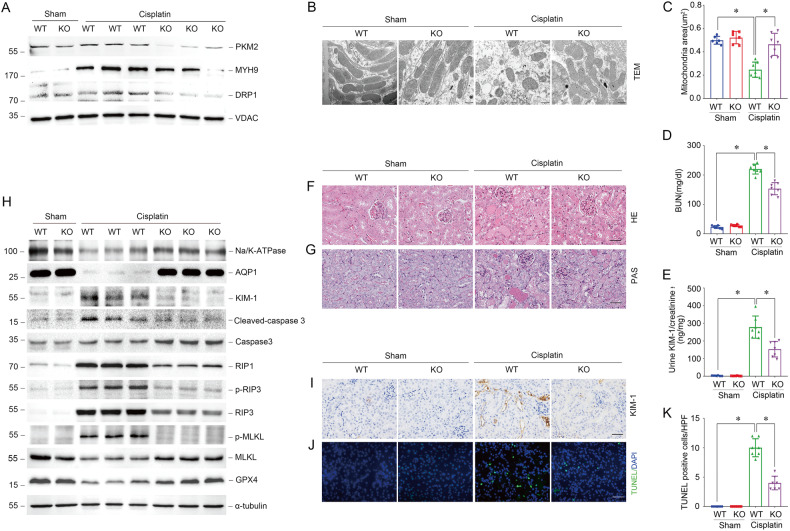
Deletion of PKM2 in tubular epithelial cells inhibits mitochondrial fragmentation and alleviates cisplatin-induced AKI in vivo. **A** Western blots result of mitochondrial PKM2, MYH9, and DRP1 expression in tubules from tubular-specific *Pkm2* knockout mice (KO) compared to wild-type mice (WT) 1 day after cisplatin injury. **B**, **C** Representative images of Transmissionelectron microscopy (TEM) and quantification of mean mitochondrial area (μm^2^) in proximal tubular epithelial cells from WT mice and KO mice 3 days after cisplatin injury. scale bar = 500 nm. *P < 0.05, *n* = 6–7 per group. **D**, **E** The BUN level or urinary KIM-1 to creatinine ratio of WT mice and KO mice 3 days after cisplatin injury. *P < 0.05, *n* = 6–7 per group. **F**, **G** Representative images of H&E and PAS staining of kidney tissues from WT mice and KO mice 3 days after cisplatin injury. scale bar = 50 μm. **H** Western blot analysis of Na/K-ATPase, AQP1, KIM-1, cleaved-caspase3, caspase3, RIP1, p-RIP3, RIP3, p-MLKL, MLKL, and GPX4 expression in kidney tissues from WT mice and KO mice 3 days after cisplatin injury. **I**, **J** Representative images of immunohistochemical and immunofluorescent staining for KIM-1, TUNEL in kidney tissues from WT mice and KO mice 3 days after cisplatin injury. scale bar = 50 μm. (K) Quantitative analysis of TUNEL positive cells among groups as indicated. scale bar = 50 μm. *P < 0.05, *n* = 6–7 per group.

**Fig. 6:**
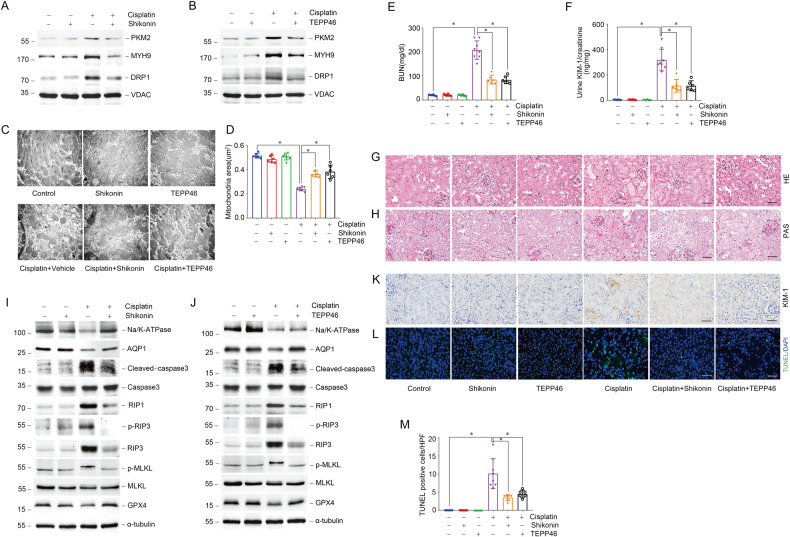
Regulating PKM2 activity alleviates cisplatin-induced AKI in vivo. **A** Western blots result of PKM2, MYH9, and DRP1 expression in mitochondria isolated from mice after cisplatin injection at day 1 pretreated with or without Shikonin. **B** Western blot results of PKM2, MYH9, and DRP1 expression in mitochondria isolated from mice after cisplatin injection at day 1 pretreated with or without TEPP46. **C**, **D** Representative images of TEM and quantification of mean mitochondrial area (μm^2^) in proximal tubular epithelial cells from cisplatin injected mice pretreated Shikonin or TEPP46. scale bar = 500 nm. *P < 0.05, *n* = 6–8 per group. **E**, **F** The BUN level or urinary KIM-1 to creatinine ratio of mice 3 days after cisplatin injury pretreated with Shikonin or TEPP46. *P <0.05, *n* = 6–8 per group. **G**, **H** Representative images of H&E and PAS staining of kidney tissues from cisplatin injected mice pretreated with Shikonin or TEPP46. scale bar=50 μm. **I** Western blot analysis of Na/K-ATPase, AQP1, KIM-1, cleaved-caspase3, caspase3, RIP1, p-RIP3, RIP3, p-MLKL, MLKL, and GPX4 expression in kidney tissues from cisplatin injected mice pretreated with or without Shikonin. **J** Western blot analysis of Na/K-ATPase, AQP1, KIM-1, cleaved-caspase3, caspase3, RIP1, p-RIP3, RIP3, p-MLKL, MLKL, and GPX4 expression in kidney tissues from cisplatin injected mice pretreated with or without TEPP46. **K**, **L** Representative images of immunohistochemical and immunofluorescent staining for KIM-1, TUNEL in kidney tissues from cisplatin injected mice pretreated with Shikonin or TEPP46. scale bar = 50 μm. **M** Quantitative analysis of TUNEL positive cells among groups as indicated. scale bar = 50 μm. *P < 0.05, *n* = 6–8 per group.

To further determine the effect of PKM2 on AKI, we generated tubular epithelial cells specific *Pkm2* knockout mice using Cre-Loxp recombinase technology. Genotyping of wild-type (ggt-PKM2^+/+^, WT) and knockout (ggt-PKM2^-/-^, KO) mice was performed (Supplementary Fig. 6). Consistent with the founding in vitro, PKM2 deletion in PTCs inhibited the expression of p-DRP1, mitochondrial MYH9, and DRP1, and alleviated mitochondrial fragmentation induced by cisplatin injection (Fig. 5A; Supplementary Fig. 4G). The mitochondrial area in PTCs was also improved in KO mice (Fig. 5B, C). Compared with WT mice, the blood urea nitrogen (BUN) and urinary kidney injury molecule 1 (KIM-1) expression levels were reduced in KO mice (Fig. 5D, E). As expected, *Pkm2* knockout in PTCs remarkably reversed morphological abnormalities such as renal tubule detachment, loss of brush border, and tubular crystal formation induced by cisplatin (Fig. 5F, G). Notably, the expression of KIM-1, cleaved-caspase3, RIP1, p-RIP3, RIP3, and p-MLKL was decreased, and the Na/K-ATPase, aquaporin 1 (AQP1), and GPX4 expressions were increased in kidney tissues from KO mice compared with WT mice after cisplatin injection (Fig. 5H). Immunohistochemical and immunofluorescent staining further confirmed that less KIM-1 positive cells and TUNEL positive cells was found in PTCs from KO mice compared to WT mice. (Fig. 5I–K). As expected, Shikonin and TEPP46 could inhibit PKM2 translocation into mitochondria, reduce mitochondrial MHY9 and DRP1 expression, and inhibited cisplatin-induced mitochondrial fragmentation, renal tubular injury, and cell death. (Fig. 6; Supplementary Fig. 4H, I).

## Discussion

In our study, we demonstrate that PKM2 translocates into mitochondria in renal tubular epithelial cells during AKI induced by cisplatin. Then, mitochondrial PKM2 binding to MYH9 promotes DRP1-mediated mitochondrial fragmentation. Knockout *Pkm2* or inhibiting PKM2 phosphrylation by Shikonin or promote PKM2 tetramerization by TEPP46 could inhibit mitochondrial fragmentation and alleviate AKI.

PKM2 is not only a key enzyme involved in regulating glycolysis but is also a vital protein that regulates cell survival [26, 41]. The role of PKM2 in cell survival is controversial. Several studies have found that PKM2 promoted tumor cell proliferation, inhibition of PKM2 expression could induce tumor cell death [42, 43]. However, several studies have shown that PKM2 plays different roles in acute injury. Treatment with TEPP46 to tetrameric PKM2 could prevent doxorubicin-induced p53 up-regulation and apoptosis in cardiomyocytes [44]. TEPP46 also showed the pro-survival ability during LPS or D-galactosamine (D-Gal) induced liver injury [45]. Furthermore, podocyte specific deletion of PKM2 ameliorated LPS-induced podocyte injury [46]. In this study, we found that the specific loss of PKM2 or inhibition of PKM2 phosphorylation or dimerization could promote the survival of renal tubular epithelial cells in cisplatin-induced AKI. The different effects of PKM2 may be related to the acute and chronic stages of the disease and the ability of cell proliferation.

Several studies have shown that PKM2 regulation is related to tetramer depolymerization, dimer formation, and subcellular localization [31, 33, 47]. The inactive PKM2 dimer leads to the accumulation of glycolysis intermediates and provides synthetic substrates for cell proliferation [48]. However, Proton Nuclear Magnetic Resonance (1H NMR) and Liquid Chromatography Mass Spectroscopy (LCMS) analysis of the metabolic changes in NRK-52E cells after cisplatin stimulation showed that the lactate acid levels did not increase but slightly decreased [49]. In our experiment, cisplatin treatment did not up-regulate the expression of glycolytic related proteins such as hexokinase 2 (HK2), pyruvate dehydrogenase kinase, isozyme 1 (PDK1), and the lactic acid level was not increased after cisplatin stimulation (data not shown). Therefore, PKM2 dimer formation upon cisplatin stimulation does not promote glycolysis. The formation of the PKM2 dimer may promote the localization of PKM2 to perform its biological functions in different subcellular structure. We found that p-PKM2 in renal tubular epithelial cells promotes the formation of PKM2 dimers and the mitochondrial translocation of PKM2. Liang et al. [33] first showed that PKM2 translocation into mitochondria regulates cell survival under oxidative stress. In contrast, PKM2 translocation into the mitochondria was significantly reduced by inhibiting PKM2 phosphorylation by Shikonin, or promoting PKM2 tetramer formation by TEPP46. We suggest that PKM2 may regulate cell survival by interfering with mitochondrial homeostasis.

Renal tubular cells require large amounts of ATP for transcellular transport and tubular reabsorption [50]. Mitochondria are highly dynamic organelles that regulate fusion and fission to maintain their shape and function. Mitochondrial damage and dysfunction are considered as the major factor leading to AKI [51]. During AKI, mitochondrial dynamic changes from fusion to fission, lead to mitochondrial fragmentation in numerous renal tubular cells [11]. Abnormalities in mitochondrial structure, function and homeostasis of renal tubule cells were also observed during cisplatin-induced AKI [52]. Studies have shown that DRP1 activates membrane bound BAX by binding to the N-terminal region of BAX, causing BAX activation and oligomerization of conformational change, leading to the loss of mitochondrial membrane potential, cytochrome C released into the cytoplasm, activating caspase, and ultimately inducing apoptosis [53]. In addition, DRP1-dependent mitochondrial fragmentation has been shown to be possible by stimulating the opening of mitochondrial permeability transition pores (mPTP), mitochondrial membrane potential (Δ*ψ*) depolarization, autophagosome formation, and the induction of mitochondrial autophagy. Stimulating autophagosome formation requires increasing cell ROS levels, stimulating lipid peroxidation and glutathione (GSH) depletion, ultimately leading to necroptosis and ferroptosis [54, 55].

MYH9 encodes non-muscular myosin IIA (NMIIA), which plays a role in cell adhesion, migration, proliferation, and differentiation [56], and is associated with several human syndromes, including kidney disease, hearing impairment, thrombocytopenia, and cataract [57, 58]. MYH9 was reported to promote mitochondrial fission, and inhibiting MYH9 could maintain mitochondrial integrity [59, 60]. Actin polymerization resulted in significant MYH9 enrichment at mitochondrial contraction sites, whereas the distribution of DRP1 in mitochondria decreased significantly after MYH9 inhibition, suggesting that MYH9 may enhance DRP1 accumulation and fission at mitochondrial fission sites [37]. MYH9 could activate EGFR-AKT-ERK signaling pathway [61, 62], and ERK1/2 inhibitors cloud block MYH9’s effect on DRP1 phosphorylation, which suggested that MYH9 may activate ERK to induce DRP1 phosphorylation [63]. Hu et al. [38]. found that MYH9 aggravated kidney damage in a cisplatin-induced AKI mouse model. In this study, we demonstrated that mitochondrial PKM2 interacted with MYH9 to promote DRP1 phosphorylation, leading to mitochondrial fragmentation, and cell death. This study provides a new mechanism for MHY9 to regulate DRP1-mediated mitochondrial fission in AKI.

In conclusion, we demonstrate that in the process of AKI induced by cisplatin, PKM2 enters the mitochondria and binds MHY9 to promote DRP1-mediated mitochondrial fission, resulting in mitochondrial fragmentation and promoting cell death in renal tubular epithelial cells. Inhibition of PKM2 expression or phosphorylation level, or promoting PKM2 tetramer can inhibit PKM2 translocating into mitochondria, alleviate mitochondrial fragmentation, hamper renal tubular epithelial cell death, and alleviate AKI. These selective rescue mechanisms may provide new therapeutic targets for treating AKI.

## Methods

### Animal models

Our experimental animals were male C57BL/6 J aging 6–8 weeks and weighing 18–22 g from Beijing Vital River Laboratory Animal Technology Limited Company. PKM2 in the renal proximal tubule of adult mice was knocked out by the conditional system, with exon 10 of the mouse PKM2 gene floxed. Tail DNA from all mice was genotyped by PCR analysis. All animals were in-house generated and maintained in the Specific Pathogen-Free (SPF) Experimental Animal Center of Nanjing Medical University. To establish an AKI mouse model, ggt-PKM2^+/+^ and ggt-PKM2^−/−^ mice were intraperitoneally injected with cisplatin (20 mg/kg, cat: P4394, Sigma Aldrich), and sacrificed 3 days later after cisplatin treatment. C57BL/6 J mice were gavaged consecutively by Shikonin (5 mg/kg, cat: HY-N0822/CS-5906, MCE) or TEPP46 (50 mg/kg, cat: HY-18657/CS-4865, MCE) for 3 days, followed by intraperitoneally injected with cisplatin and sacrificed 3 days later after cisplatin treatment. Blood and kidney samples were collected for further analysis.

### Cell culture and treatment

NRK-52E cells were derived from Shanghai Zhongqiaoxinzhou Biotechnology Limited Company. The cells were cultured in DMEM high glucose medium containing 1% penicillin/streptomycin (PS, cat: 15140, Sigma Aldrich) and 5% fetal bovine serum (FBS, cat: A3160902, Gibco). PTCs were cultured under sterile conditions from kidneys isolated from C57BL/6 J mice (about 7 days) digested of collagenase by improving the previously described method [64]. The cells were treated with staurosporine (0.1 μM, cat: 569397, Millipore), cisplatin (25 μg/ml, cat: P4394, Sigma Aldrich), Shikonin (1 μM, cat: HY-N0822/CS-5906, MCE), TEPP46 (10 μM, cat: HY-18657/CS-4865, MCE) or Blebbistatin (10 μM, cat: HY-13441/CS-4983, MCE) as indicated. Moreover, the cells were transfected with lentivirus-*Pkm2* shRNA to down-regulate PKM2 (the serial number: GGCCATTATCGTGCTCACCAA, The Genechem Company, Shanghai, China) expression and transfected with siRNA to down-regulate *Myh9* (sense: 5’-GAGACAAUGGAGGCCAUGAUU-3’, anti-sense: 5’-UCAUGGCCUCCAUUGUCUCUU-3’, The Integrated Biotech Solutions, Shanghai, China) expression according to the manufacturer’s instruction.

### Cross-linking PKM2 to evaluate the formation of monomer, dimer, and tetramer

Cells were counted and the samples were cross-linked with 5 mM dissuccinimide (DSS, cat: 21555, Thermo Scientific) at room temperature for 30 min as indicated by the protocol. In the kidney cross-linking assay, the samples were cross-linked with 250 µM DSS at room temperature for 30 min. The samples of cells or kidney tissues were collected by SDS-polyacrylamide gel electrophoresis sample loading buffer (cat: P0015A, Beyotime) and boiled for 10 min. Samples were separated by 10% SDS-PAGE.

### Nuclear extraction

Cells were collected by centrifugal method. Nuclear and cytoplasm extraction were carried out in accordance with the manufacturer’s protocol (cat: 78833, Thermo Scientific). Simply, 10 × 10^6^ cells were centrifuged at 500 g for 5 min to obtain precipitation added by cytoplasmic extraction reagent I (CER I). After incubation in ice after intense vortex for 10 min, cytoplasmic extraction reagent II (CER II) was added and centrifuged at 16,000 g for 5 min to obtain the supernatant as cytoplasm. A pre-cooled nucleoprotein extraction reagent (NER) was added to the precipitate and vortices violently for 15 s. The specimen was placed on ice and continued to swirl every 10 min for 15 s for a total of 40 min, followed by centrifugation at 16,000 g for 10 min to obtain the nuclear as the supernatant.

### Mitochondrial isolation

We collect cells or treat kidney tissues according to instructions. Appropriate amount of mitochondrial isolation reagent was added to the precipitated cells (cat: C3601, Beyotime) or kidney tissues (cat: C3606, Beyotime) with placed in ice for 15 min. The suspension was transferred to a homogenizer for moderate homogenization. After centrifuged at 600 g for 10 minutes, supernatant was centrifuged at 11,000 g for 10 min again. The precipitates are the isolated mitochondria.

### CCK-8 assay

NRK-52E cells were inoculated at a concentration of 5 × 10^3^ cells/ml on a 96-well plate. 10 µL/well CCK-8 solution (cat: A311-01/02, Vazyme) was added to each well and incubated at 37 °C for 1 h. The cells were counted by measuring absorbance at 450 nm.

### TUNEL staining

TUNEL staining (cat: G3250, Promega) was performed according to the manufacturer’s instructions. Positive-stained nucleus were counted in fields of ×400 images. Semi-quantitative analysis was performed by randomly counting selected areas in each kidney tissue or each well of cells.

### Co-Immunoprecipitation

Cells were treated with staurosporine. The cells were resuspended in RIPA buffer in the presence of protease inhibitors. Preclear lysate by adding appropriate control IgG (normal mouse IgG, cat: sc-2025, Santa), together with Protein G/A PLUS-Agarose (cat: sc-2003, Santa), incubated at 4 °C for 1 h. After centrifuging, supernatant was transferred to a fresh microcentrifuge tube. PKM2 (cat: 60268-1-Ig, Proteintech) or DRP1 (cat: ab14647, CST) antibody were incubated at 4 °C overnight. Then, Protein A/G PLUS-Agarose was added and incubated at 4 °C for 4 hours, collected immunoprecipitants by centrifugation and discarded the supernatant. Wash pellet 4 times with PBS buffer, bound proteins were eluted by boiling in SDS sample buffer, resolved by SDS-PAGE, and then subjected to western blot analysis.

### LC-MS/MS

Co-immunoprecipitation of PKM2 binding proteins were used as the follow-up experiment as indicated. The proteolysis peptide solution was desalted, freeze-drained, and separated by Thermo UltiMate 3000 UHPLC. The peptides obtained in the liquid phase separation system were ionized and then analyzed in a series mass spectrometer. The length distribution of the peptide and the reliability of the characterization peptide identification were tested. Bioinformatics analysis was performed on the results of mass spectrometry, and mapping was performed with related software.

### Western blotting

The protein collecting and western blotting were performed according to the protocol. The primary antibodies were as follows: anti-p-PKM2 (Tyr^105^) (cat: 3827 s, CST), anti-PKM2 (cat: 4053 s, CST), anti-Na/K-ATPase (cat: 3010 s, CST; cat: ab76020, Abcam), anti-AQP1 (cat: ab168387, Abcam), anti-KIM-1 (cat: ab47635, Abcam), cleaved-caspase3 (cat: 9664 s, CST), caspase 3 (cat: 9662 s, CST), anti-RIP1 (cat: 3493 s, CST), anti-p-RIP3 (cat: ab222320, Abcam), anti-RIP3 (cat: 15828 s, CST), anti-p-MLKL (cat: 37333, CST), and anti-MLKL (cat: 37705, CST), anti-GPX4 (cat: ab125066, Abcam), anti-p-DRP1 (cat: 3455, CST), anti-DRP1 (cat: ab184247, Abcam), anti-MYH9 (cat: 11128-1-AP, Proteintech), anti-VDAC (cat: 4866 s, CST; cat: ab14734, Abcam), anti-TBP (cat: ab818, Abcam), and anti-α- tubulin (cat: T9026, Sigma Aldrich).

### BUN and urinary creatinine assays

BUN was determined using a QuantiChrom^TM^ Urea Assay Kit (DIUR-500, Bioassay Systems), and urinary creatinine was determined using a QuantiChrom^TM^ Creatinine Assay Kit (DICT-500, Bioassay Systems) according to the manufacturer’s instructions.

### Urinary KIM-1 level

Urinary KIM-1 level was measured by the Mouse TIM-1/KIM-1/HAVCR Quantikine ELISA Kit (MKM100, R&D System) according to the manufacturer’s instructions. Urinary creatinine level corrects urinary KIM-1 level.

### Histology assay

The kidney samples were fixed in a light lens fixative and kept at room temperature overnight. Paraffin-embedded specimens were sectioned 3 μm thick, dewaxed, and stained with H&E or PAS. Each tissue section images at least eight non-overlapping regions.

### TEM

Kidney tissues were fixed according to instructions. The structure and size of mitochondria randomly were observed at the same magnification in a FEI Tecnai T20 TEM (Thermo Fisher Scientific, Carlsbad, CA, USA), operated at 120 kV. The mitochondrial areas were scanned and analyzed by Image J, and GraphPad Prism Software was used for statistical analysis of the data.

### Mitochondrial immunofluorescent staining

250 nM Mitotracker Red (Molecular Probes, Invitrogen, USA) was incubated with the cells for 30 min. Then, the cells were washed, immobilized, and blocked with a specific primary antibody, anti-PKM2 (cat: 4053 s, CST; cat: 60268-1-Ig, Proteintech) or anti-MYH9 (cat: 11128-1-AP, Proteintech) and stained with FITC labeled secondary antibodies. DAPI decontaminates the nucleus. Slides were observed with a confocal inverted laser microscope (LAM 510 Meta, Zeiss, Oberkochen, Germany).

### Statistical analyses

GraphPad Prism 8.0 software was used for statistical processing, and the obtained data was expressed as X ± SD. Comparison between two groups was performed by T test, and comparison between multiple groups was followed by one-way ANOVA. *P* < 0.05 was considered as significant difference.

### Supplementary information

Supplementary data

original data files

reproducibility checklist

## Data Availability

The mass spectrometry proteomics data have been deposited to the ProteomeXchange Consortium via the PRIDE partner repository with the dataset identifier PXD039380. Any additional information is available from the lead contact upon request.
