# Supplementary material for: Pyruvate kinase M2 regulates mitochondrial homeostasis in cisplatin-induced acute kidney injury
Source: Cell Death Dis. 2023 Oct 10;14(10):663. doi: 10.1038/s41419-023-06195-z (PMC10564883; doi:10.1038/s41419-023-06195-z)
Supplement: Supplementary file 1 — Supplementary data [file 41419_2023_6195_MOESM1_ESM.docx]

**Supplementary Figures and figure legends**

**
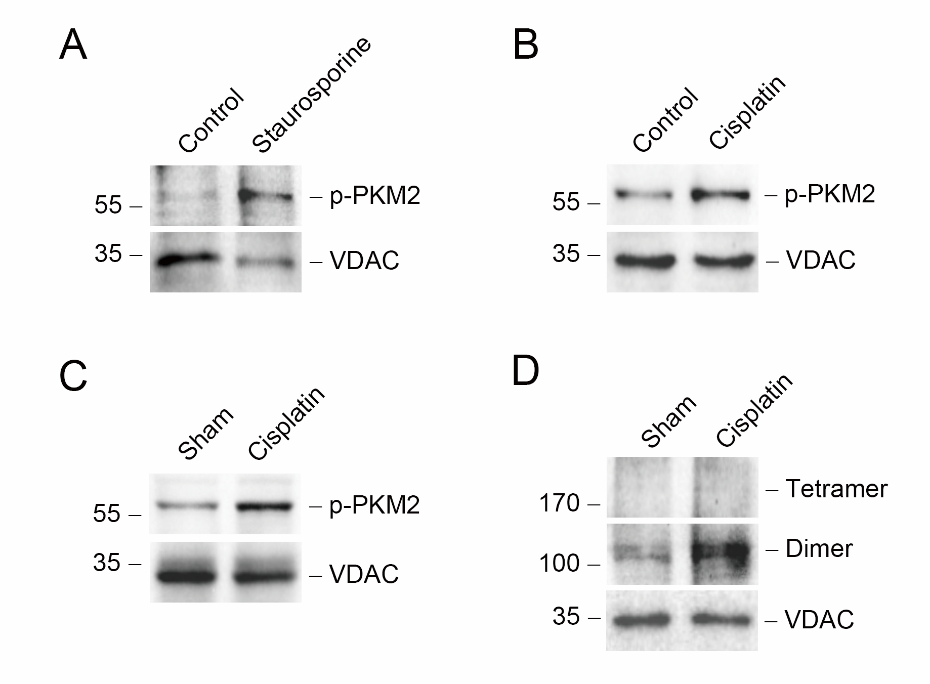
**

**Supplementary Figure 1. Mitochondrial p-PKM2 and PKM2 dimer are increased in tubular epithelial cells during acute injury**.

(A) Western blots showing the abundance of p-PKM2 in mitochondria extracted from NRK-52E cells after staurosporine treatment for 1 hour. (B) Western blot results of p-PKM2 expression in mitochondria extracted from NRK-52E cells after cisplatin treatment for 12 hours. (C) Western blot analysis of p-PKM2 expression in mitochondria isolated from mice after cisplatin injection at day 1. (D) Western blots of cross-linking mitochondria extracted from renal cortexes after cisplatin treatment to show PKM2 dimer, and tetramer.

**
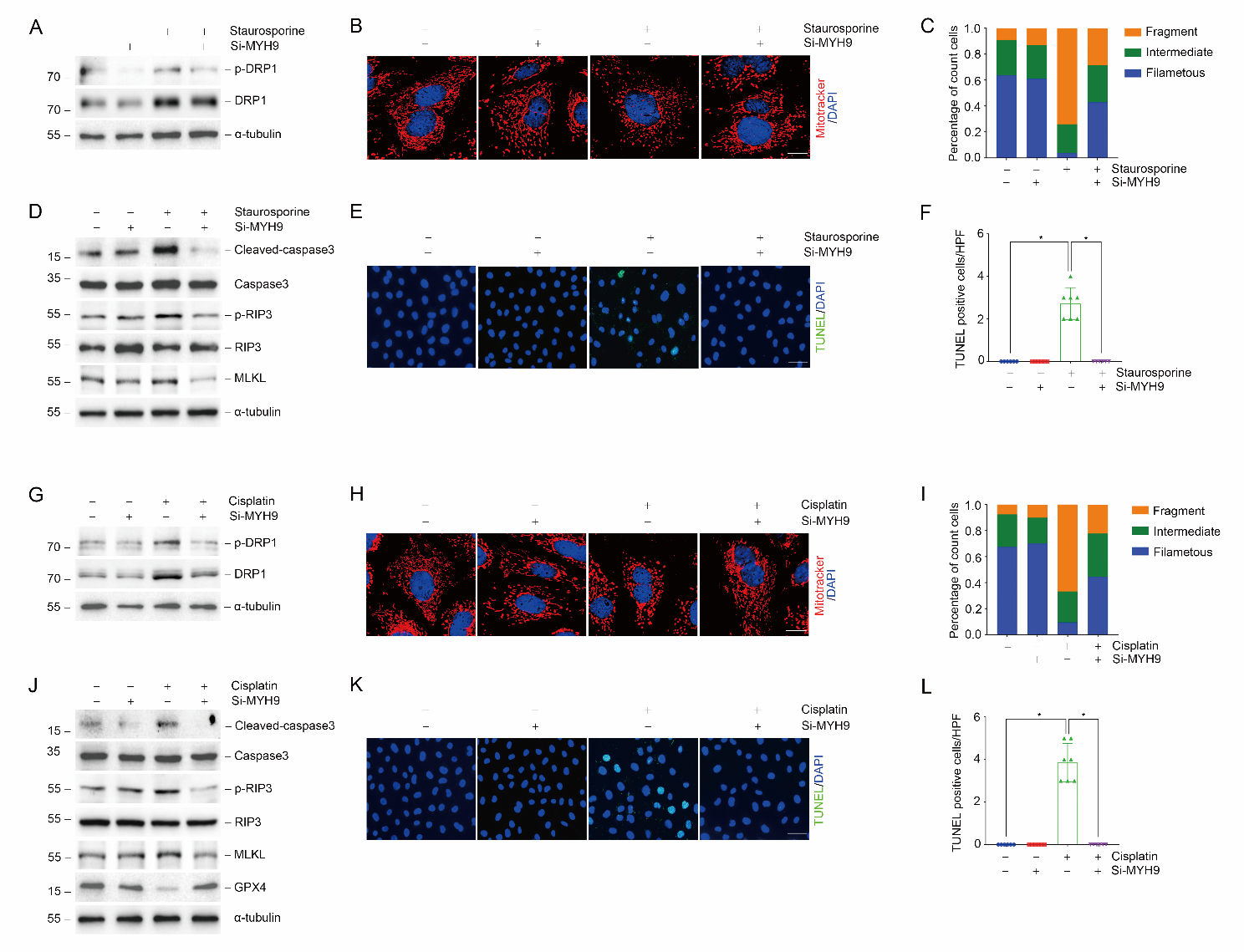
**

**Supplementary Figure 2. Inhibiting MYH9 expression limits mitochondrial fragmentation and alleviates cell death induced by staurosporine or cisplatin *in vitro*.**

(A, G) Western blots of p-DRP1 and DRP1 expression in NRK-52E cells under staurosporine stimulation 1 hour or cisplatin stimulation 12 hours pre-treated with or without *Myh9* SiRNA transfection. (B-C, H-I) Representative images of confocal immunofluorescence and percentage of mitochondrial fragmentation. Mitotracker, red; DAPI, blue. scale bar=20μm. n=5 per group. (D, J) Western blots of cleaved-caspase3, caspase 3, p-RIP3, RIP3, and MLKL expression in staurosporine or cisplatin stimulated NRK-52E cells pre-treated with or without *Myh9* SiRNA transfection. (E-F, K-L) Representative micrographs and quantitative analysis of TUNEL staining as indicated in NRK-52E cells. scale bar=50μm. **P*＜0.05, n=6~7 per group.

**
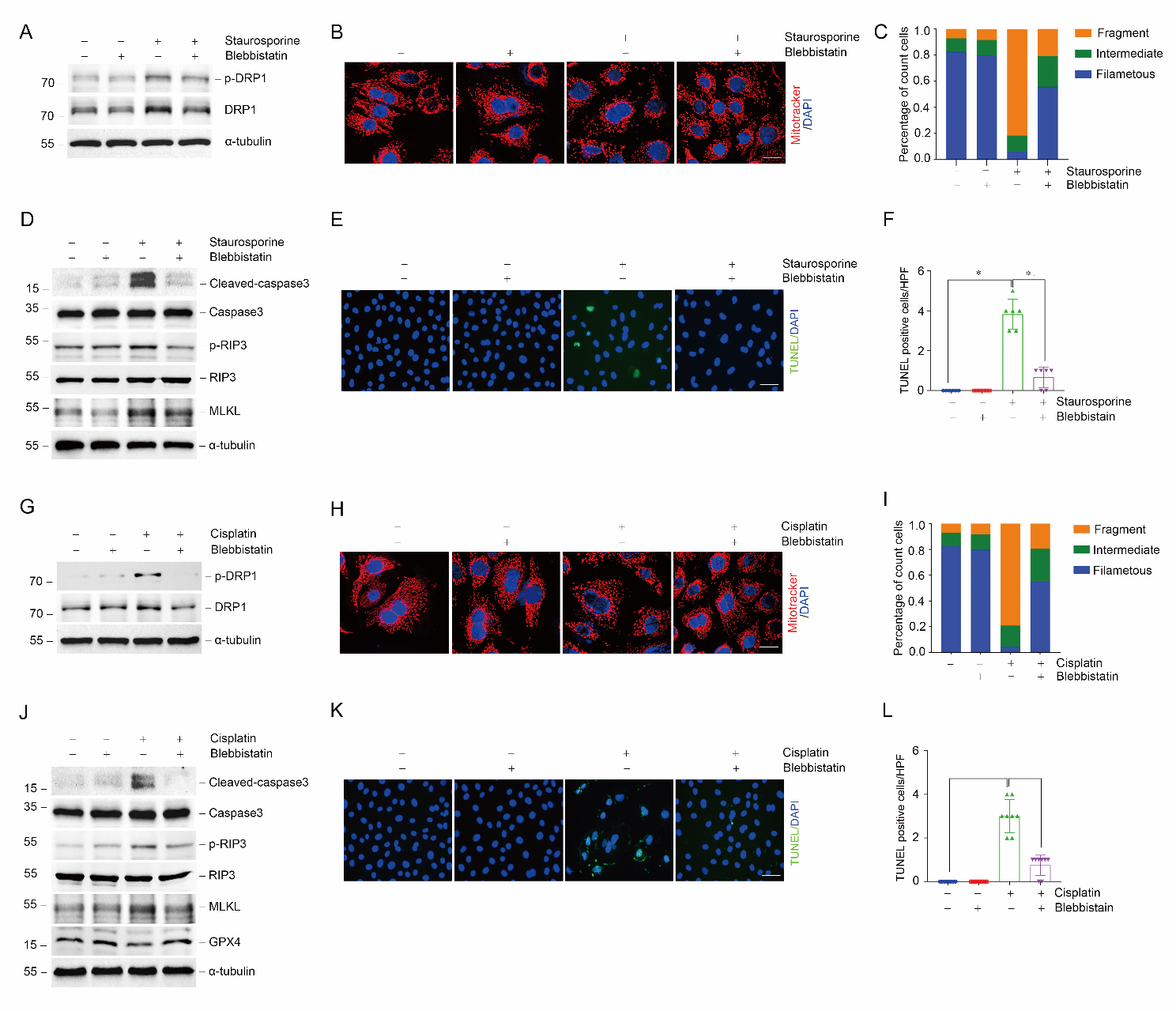
Supplementary Figure 3. Inhibiting MYH9 activity limits mitochondrial fragmentation and alleviates cell death induced by staurosporine or cisplatin *in vitro*.**

(A, G) Western blots of p-DRP1 and DRP1 expression in NRK-52E cells under staurosporine stimulation 1 hour or cisplatin stimulation 12 hours pre-treated with or without MYH9 inhibitor (Blebbistatin). (B-C, H-I) Representative images of confocal immunofluorescence and percentage of mitochondrial fragmentation. Mitotracker, red; DAPI, blue. scale bar=20μm. n=5 per group. (D, J) Western blots of cleaved-caspase3, caspase 3, p-RIP3, RIP3, MLKL, and GPX4 expression in staurosporine or cisplatin stimulated NRK-52E cells pre-treated with or without MYH9 inhibitor (Blebbistatin). (E-F, K-L) Representative micrographs and quantitative analysis of TUNEL staining as indicated in NRK-52E cells. scale bar=50μm. **P*＜0.05, n=6~8 per group.

**
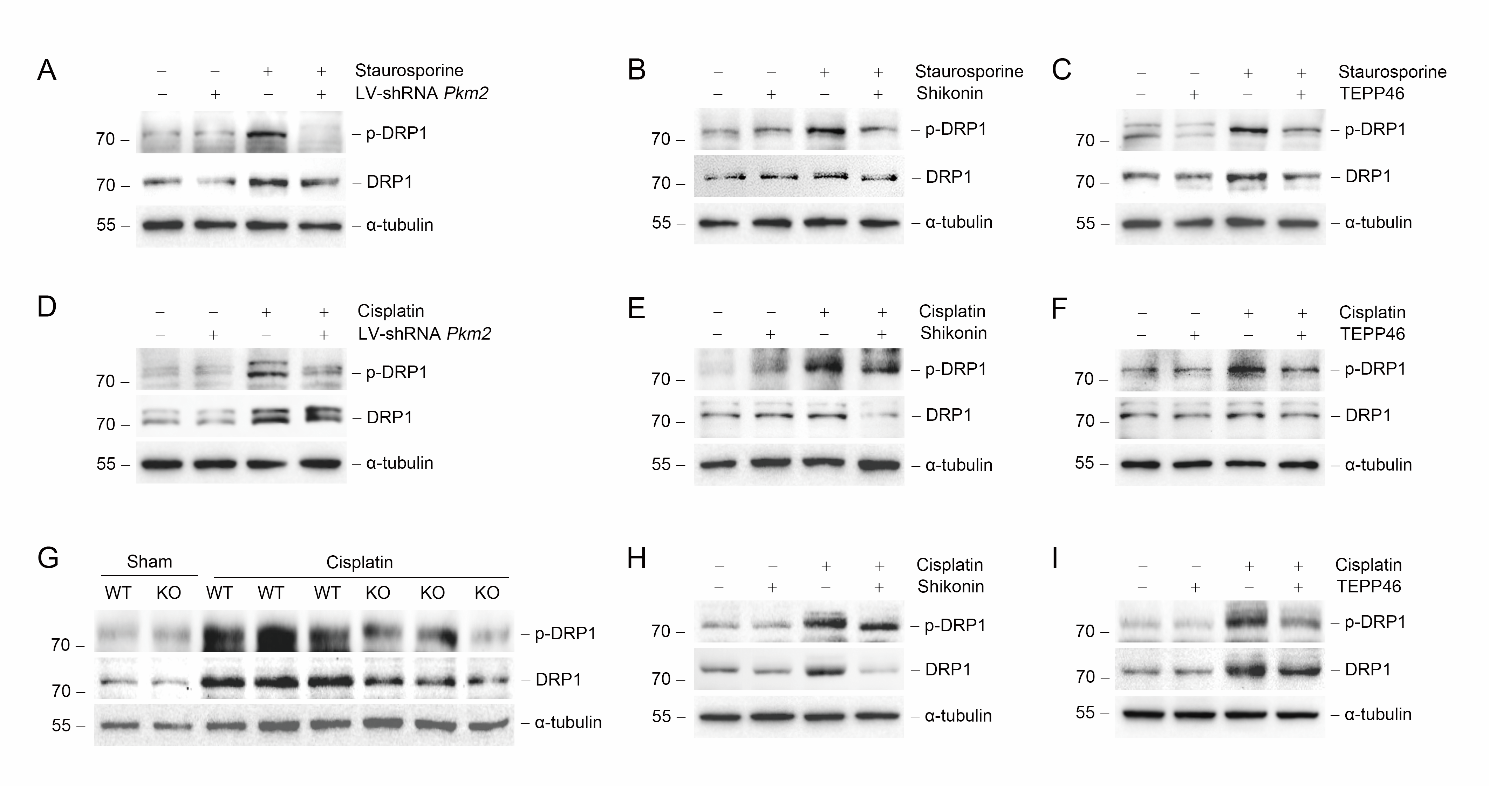
**

**Supplementary Figure 4. The role of PKM2 on DRP1 phosphorylation.**

(A) Western blots of p-DRP1 and DRP1 expression in NRK-52E cells treated 1 hour with staurosporine with or without LV-shRNA *Pkm2* transfection. (B-C) Western blots of p-DRP1 and DRP1 expression in NRK-52E cells stimulated by staurosporine pre-treated with Shikonin or TEPP46. (D) Western blots of p-DRP1 and DRP1 expression in NRK-52E cells treated 12 hours with cisplatin with or without LV-shRNA *Pkm2* transfection. (E-F) Western blots of p-DRP1 and DRP1 expression in NRK-52E cells stimulated by cisplatin pre-treated with Shikonin or TEPP46. (G) Western blots result of p-DRP1 and DRP1 expression in tubules from tubular-specific *Pkm2* knockout mice (KO) compared to wild-type mice (WT) 1 day after cisplatin injury. (H-I) Western blots result of p-DRP1 and DRP1 expression in kidney tissues from cisplatin injected at day 1 pretreated with or without Shikonin or TEPP46.


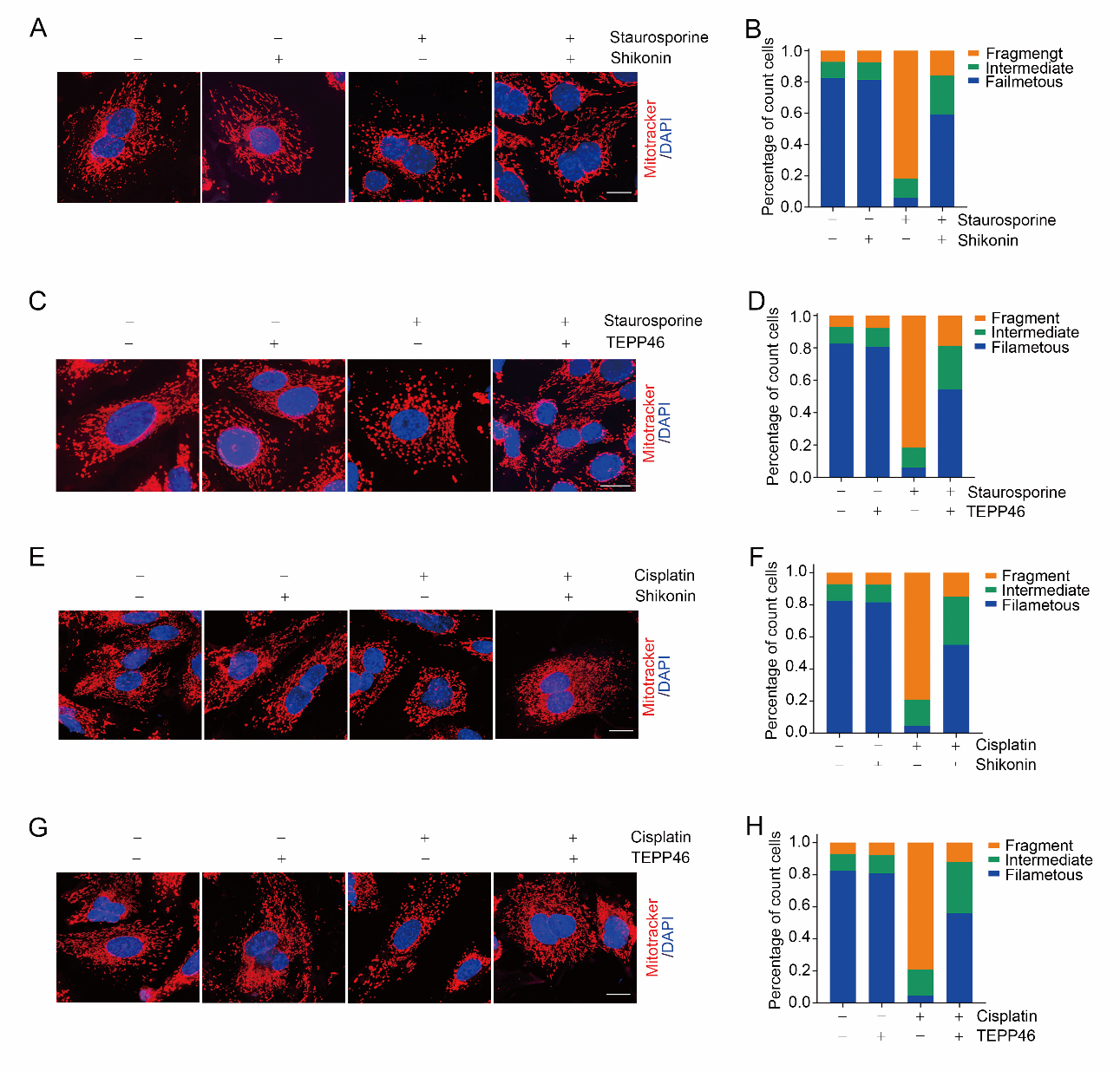


**Supplementary Figure 5. The role of PKM2 activity on mitochondrial fragmentation** **in PTCs induced by staurosporine or cisplatin.**

(A-D) Representative images of confocal immunofluorescence and percentage of mitochondrial fragmentation in staurosporine stimulated PTCs pre-treated with Shikonin or TEPP46. Mitotracker, red; DAPI, blue. scale bar=20μm. n=5 per group. (E-H) Representative images of confocal immunofluorescence and percentage of mitochondrial fragmentation in cisplatin stimulated PTCs pre-treated with Shikonin or TEPP46. Mitotracker, red; DAPI, blue. scale bar=20μm. n=5 per group.


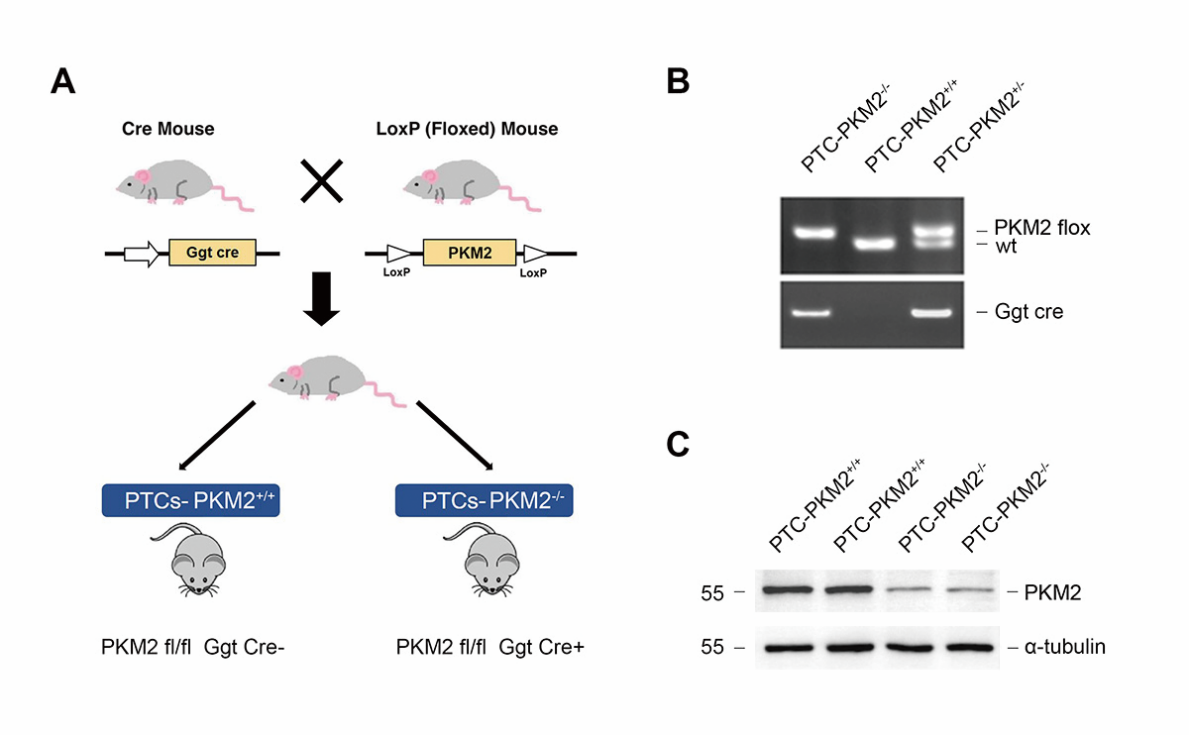


**Supplementary Figure 6. Generating mice with proximal tubular epithelial cells specific deletion of PKM2.**

A: Strategy for generating mice with deletion of PKM2 in proximal tubular epithelial cells. B: Genotyping of the mice by PCR analysis of genomic DNA. C: Western blot of PKM2 expression in proximal tubular epithelial cells isolated from WT and KO mice.
