# Supplementary material for: Pyruvate kinase M2 regulates mitochondrial homeostasis in cisplatin-induced acute kidney injury
Source: Cell Death Dis. 2023 Oct 10;14(10):663. doi: 10.1038/s41419-023-06195-z (PMC10564883; doi:10.1038/s41419-023-06195-z)

Figure 1 A

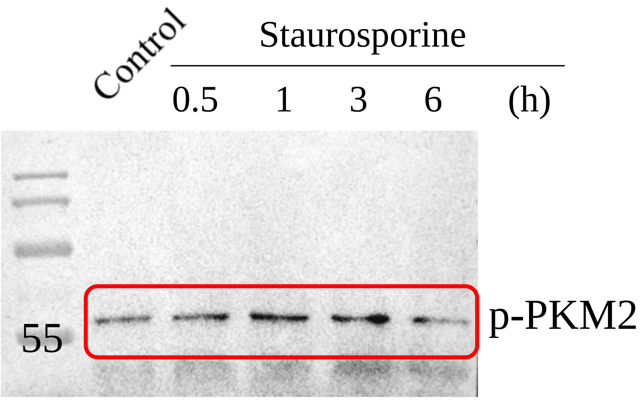
Control
Staurosporine
0.5 1 3 6 (h)
55
p-PKM2

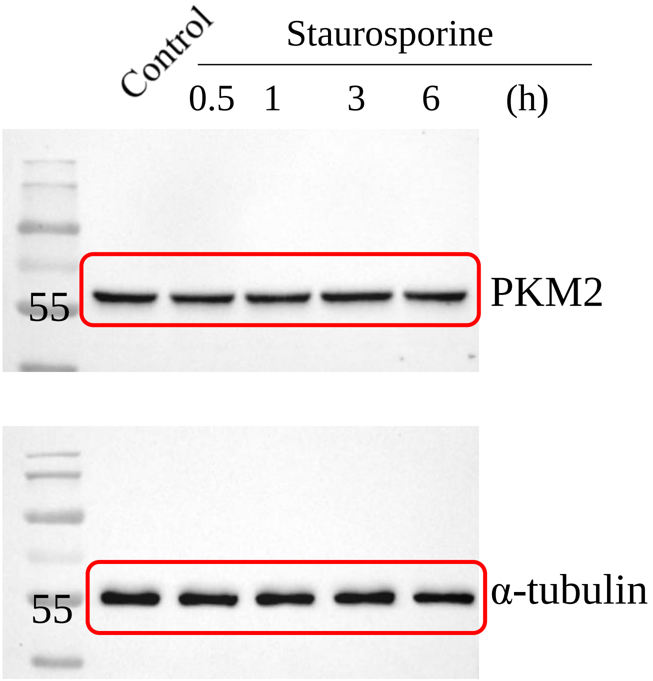
Control
Staurosporine
0.5 1 3 6 (h)
55
PKM2
55
α-tubulin

Figure 1 B

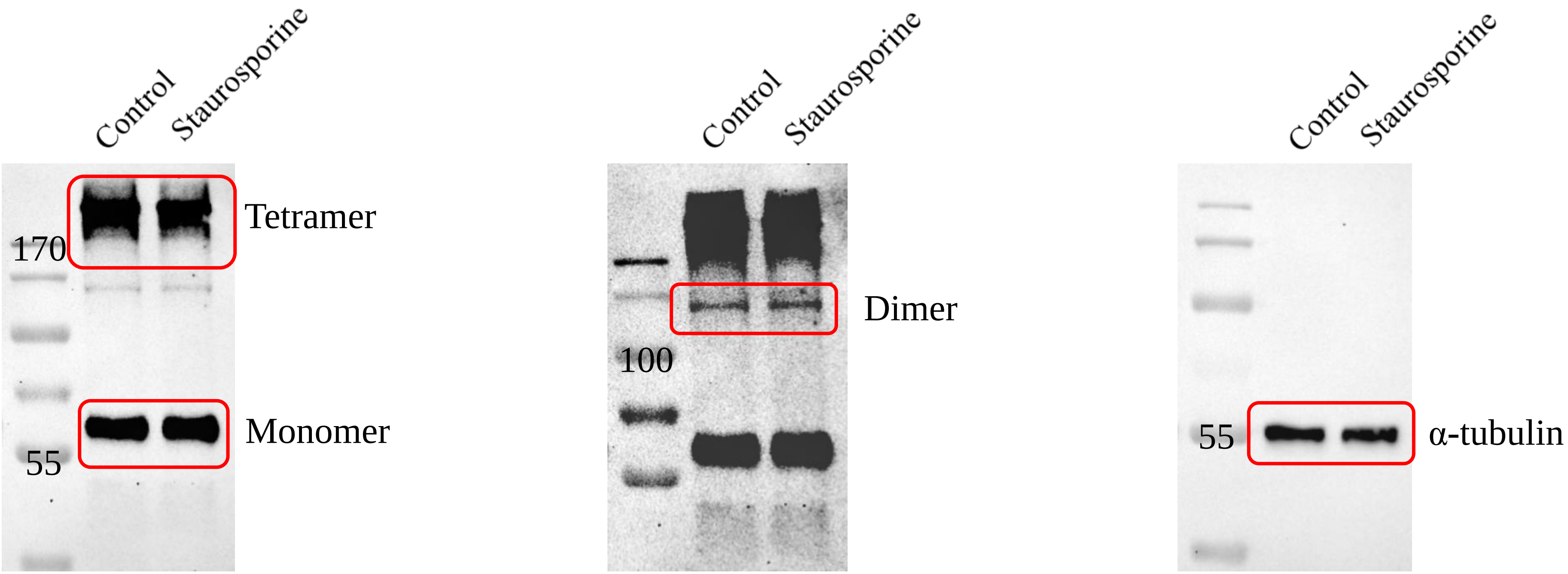
Control
Staurosporine
Tetramer
170
Monomer
55
Control
Staurosporine
Dimer
100
Control
Staurosporine
55
α-tubulin

Figure 1 C

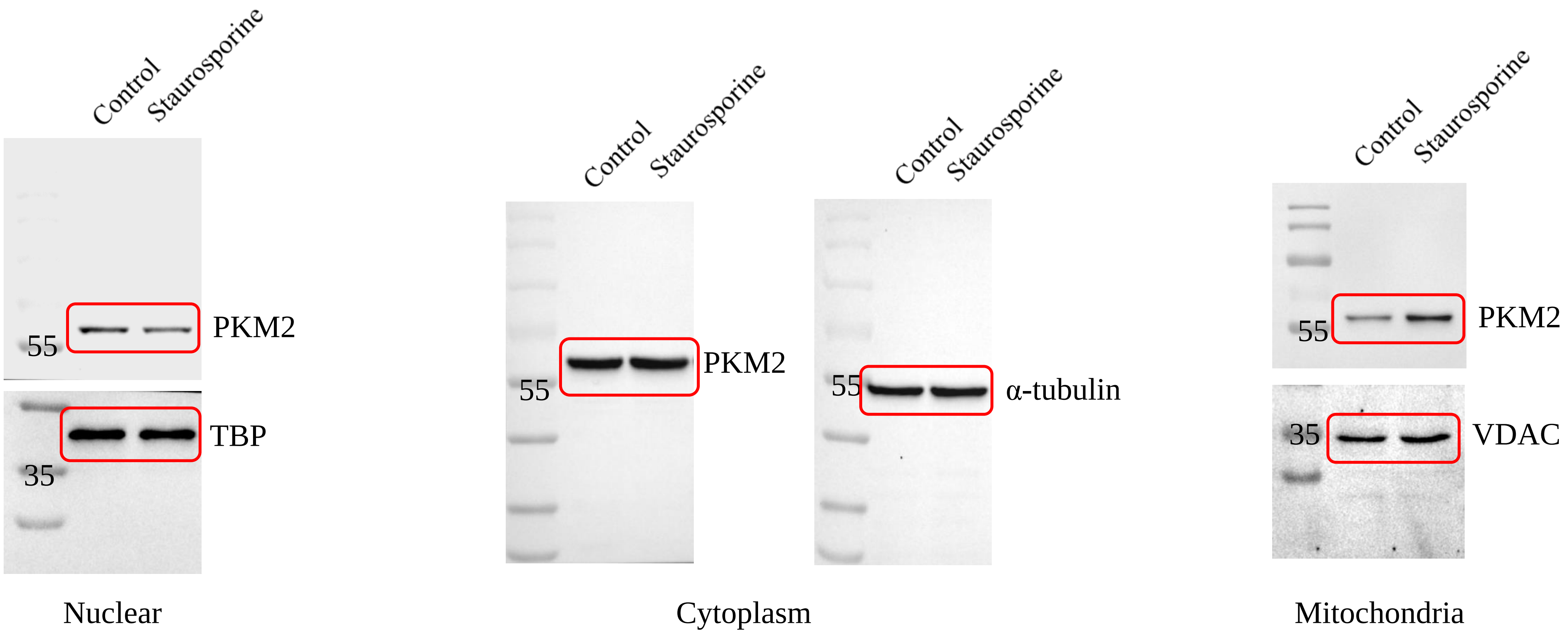
Control
Staurosporine
55
PKM2
TBP
35
Nuclear
Control
Staurosporine
55
PKM2
Control
Staurosporine
55
α-tubulin
Cytoplasm
Control
Staurosporine
55
PKM2
35
VDAC
Mitochondria

Figure 1 E

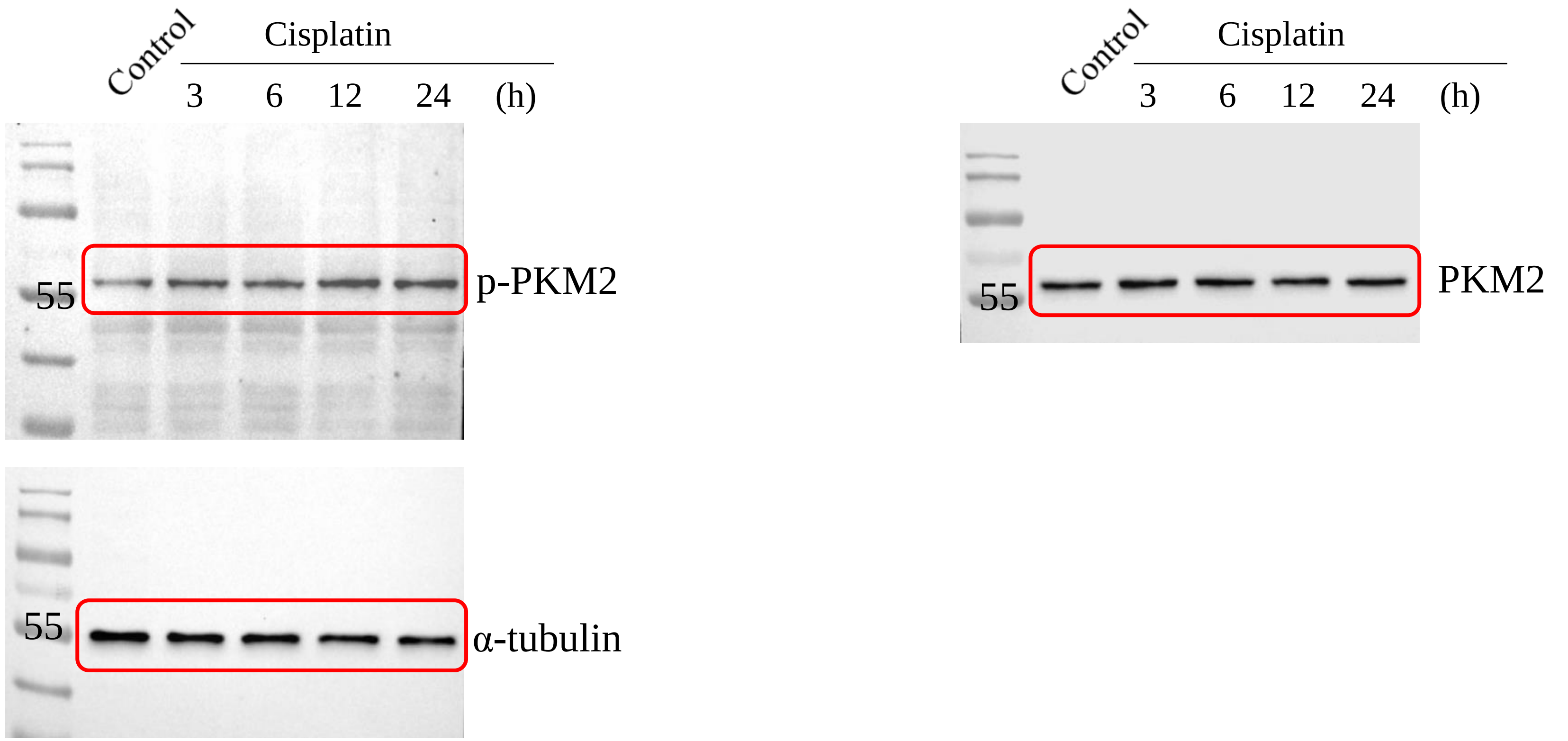
Control
Cisplatin
3
6
12
24
(h)
55
p-PKM2
55
α-tubulin
Control
Cisplatin
3
6
12
24
(h)
55
PKM2

Figure 1 F

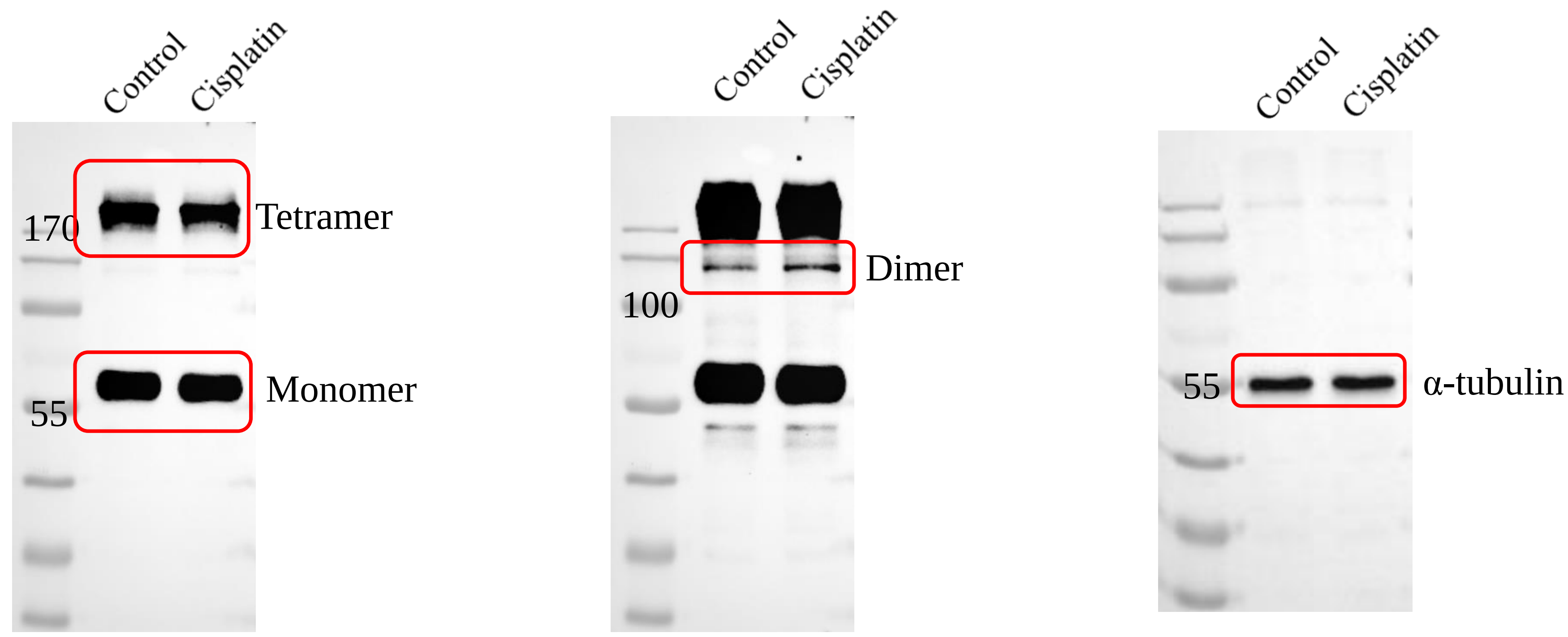

Control
Cisplatin
170
55
Tetramer
Monomer
Control
Cisplatin
100
Dimer
Control
Cisplatin
55
α-tubulin

Figure 1 G

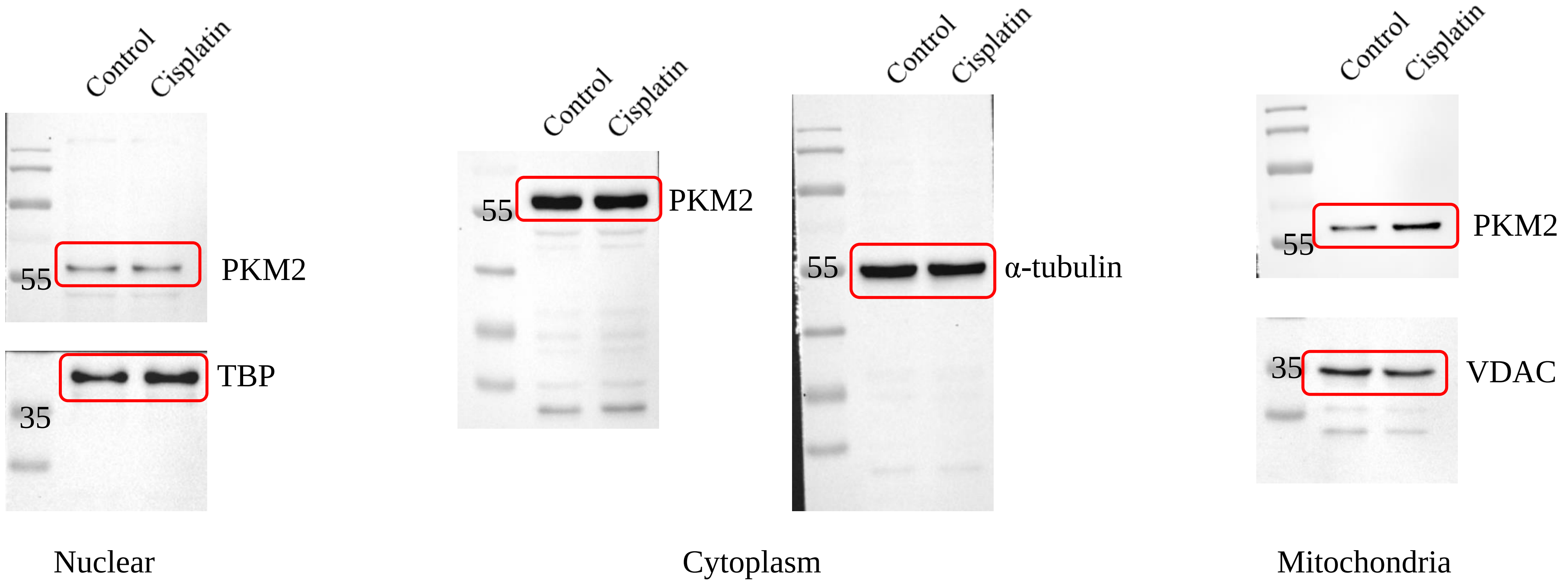

Control
Cisplatin
PKM2
55
TBP
35
Control
Cisplatin
55
PKM2
Control
Cisplatin
55
α-tubulin
Control
Cisplatin
PKM2
55
35
VDAC
Nuclear
Cytoplasm
Mitochondria

Figure 1 I

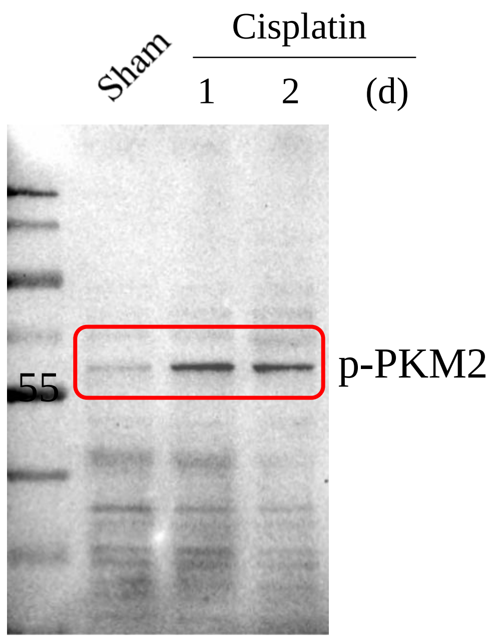
Sham
Cisplatin
1
2
(d)
55
p-PKM2

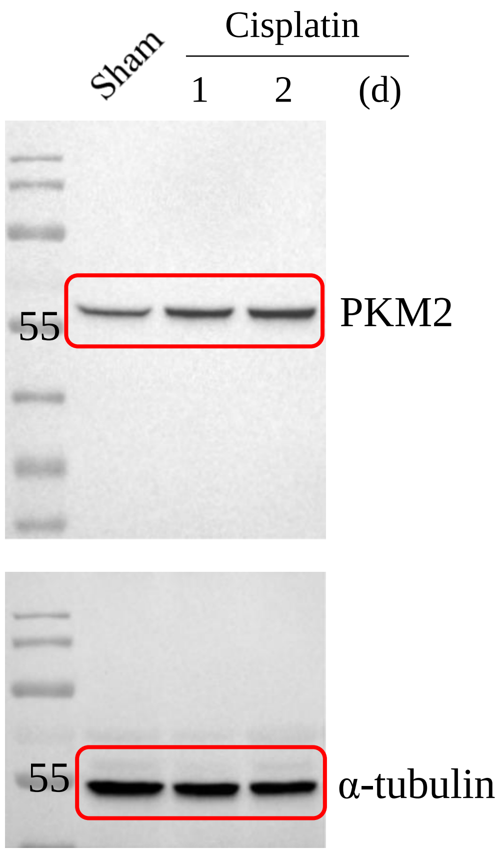
Sham
Cisplatin
1
2
(d)
55
PKM2
55
α-tubulin

Figure 1 J

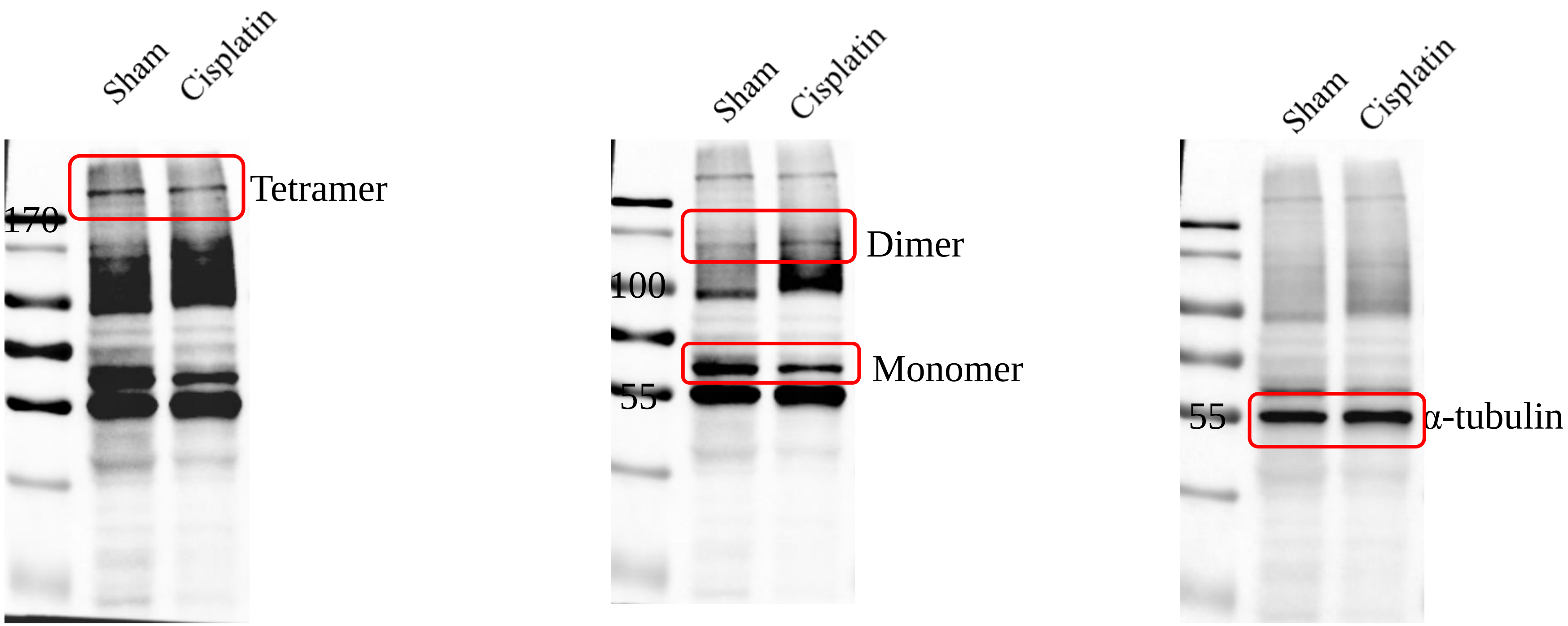

Sham
Cisplatin
Tetramer
170
Sham
Cisplatin
Dimer
100
Monomer
55
Sham
Cisplatin
55
α-tubulin

Figure 1 K

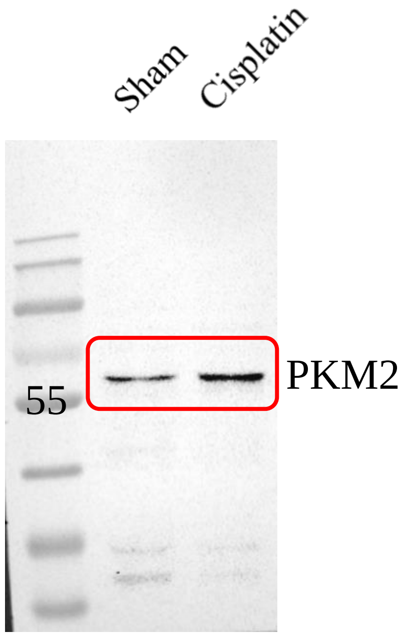

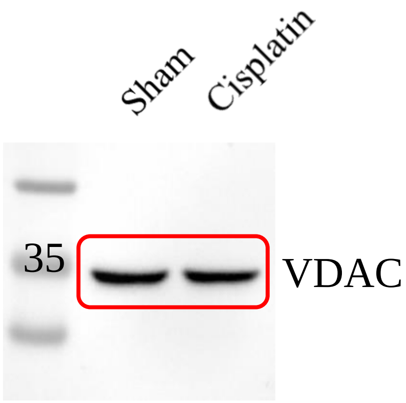

Figure 2 D

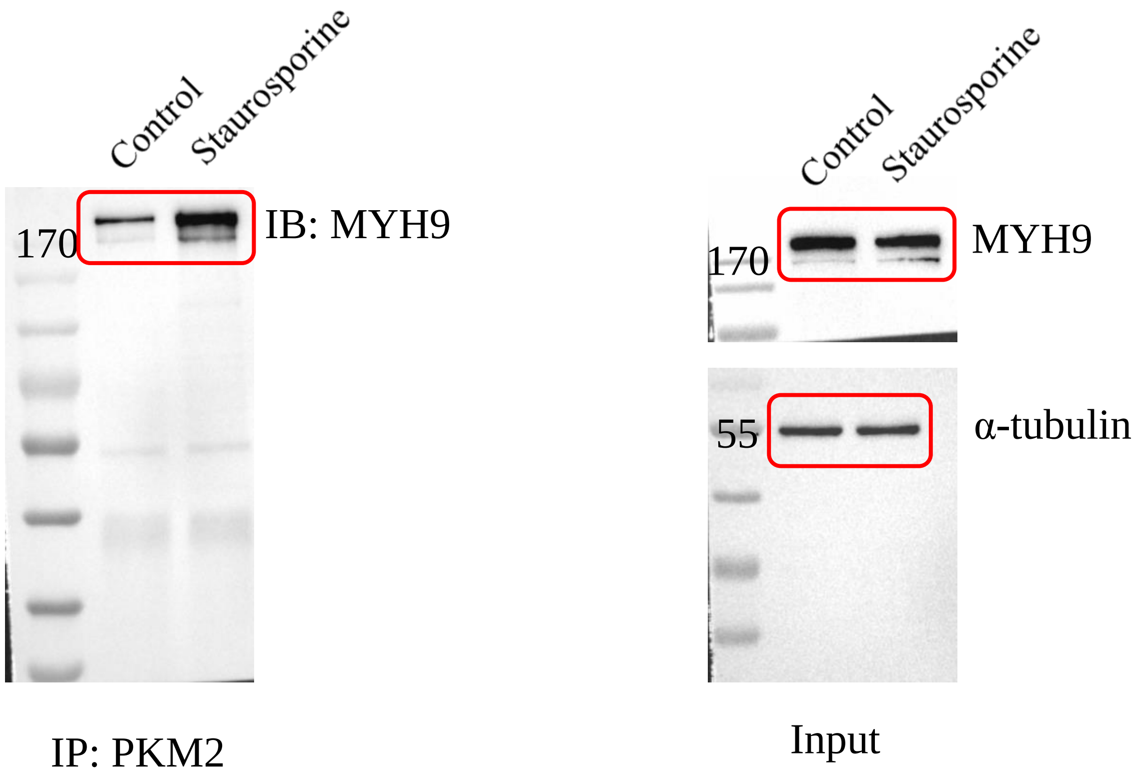

Control
Staurosporine
170
IB: MYH9
IP: PKM2
Control
Staurosporine
170
MYH9
55
α-tubulin
Input

Figure 2 E

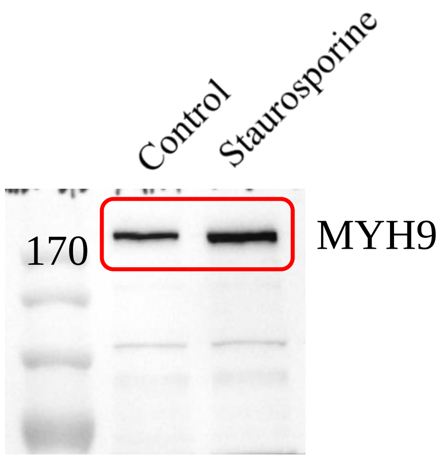

Control
Staurosporine
170
MYH9

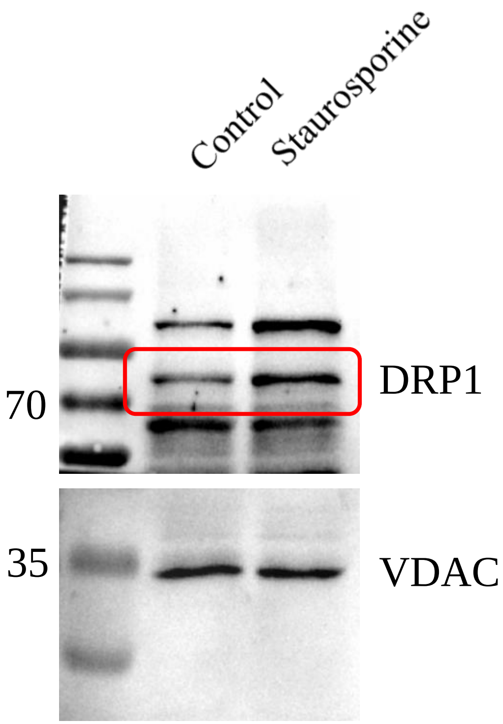

Control
Staurosporine
70
DRP1
35
VDAC

Figure 2 F

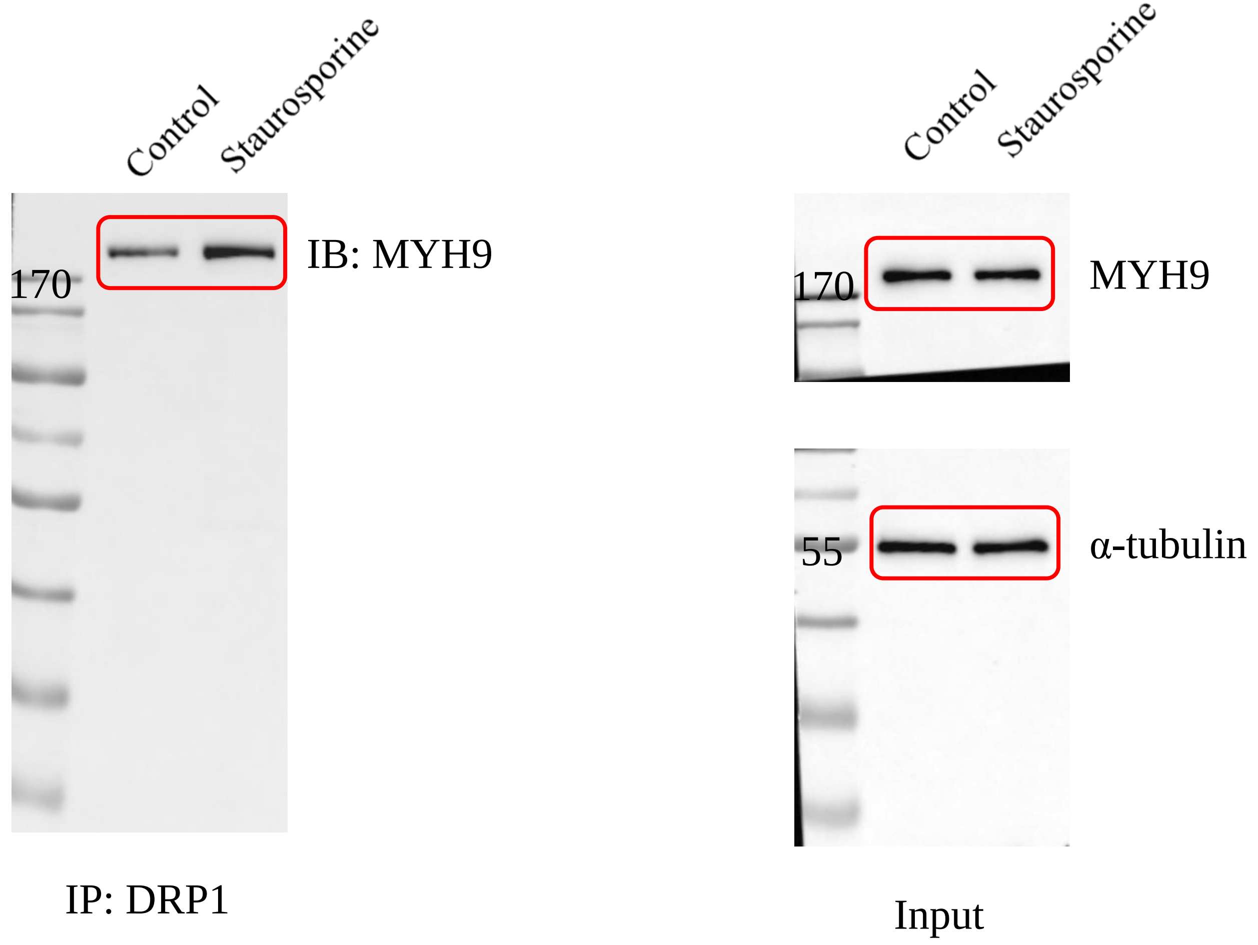
Control
Staurosporine
IB: MYH9
170
IP: DRP1
Control
Staurosporine
MYH9
170
α-tubulin
55
Input

Figure 2 G

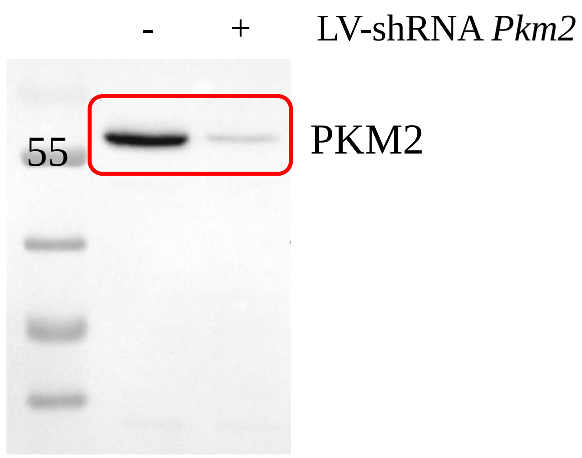

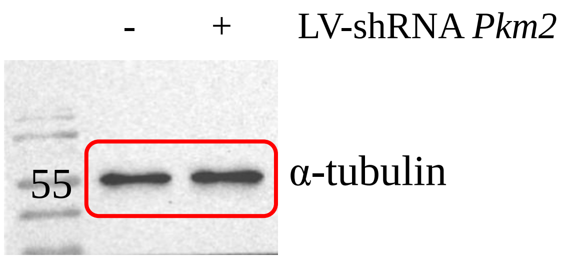

Figure 2 H

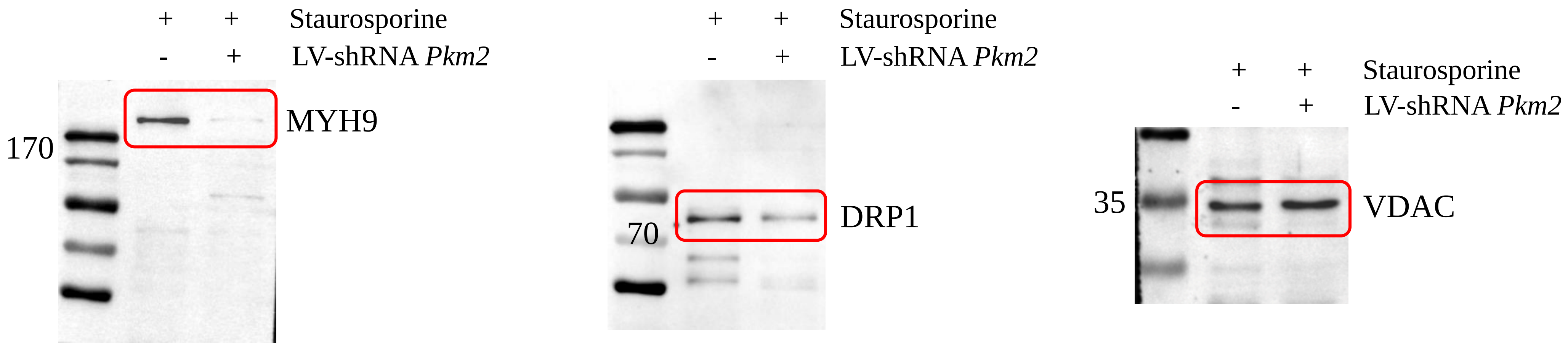

+ + Staurosporine
- + LV-shRNA *Pkm2*
170
MYH9
+ + Staurosporine
- + LV-shRNA *Pkm2*
70
DRP1
+ + Staurosporine
- + LV-shRNA *Pkm2*
35
VDAC

Figure 2 K

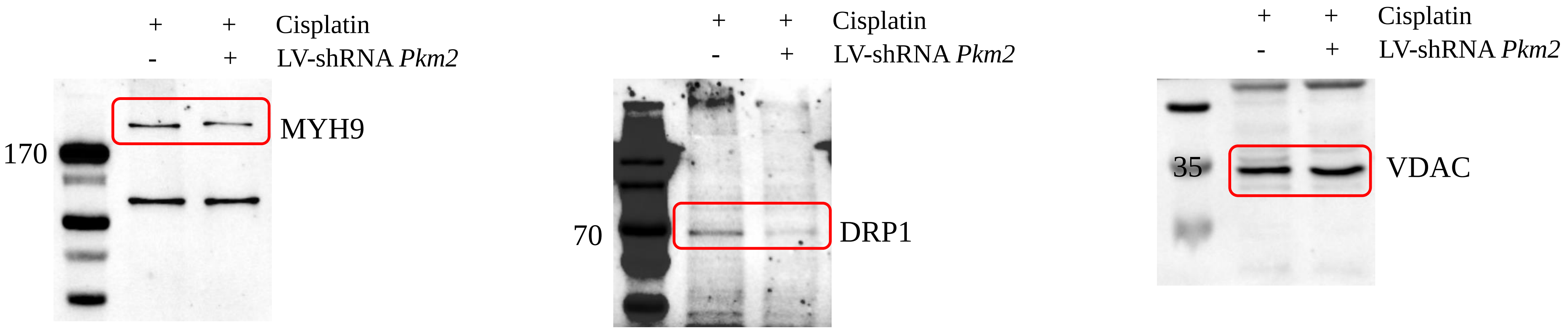

+ + Cisplatin
- + LV-shRNA *Pkm2*
170
MYH9
+ + Cisplatin
- + LV-shRNA *Pkm2*
70
DRP1
+ + Cisplatin
- + LV-shRNA *Pkm2*
35
VDAC

Figure 2 N

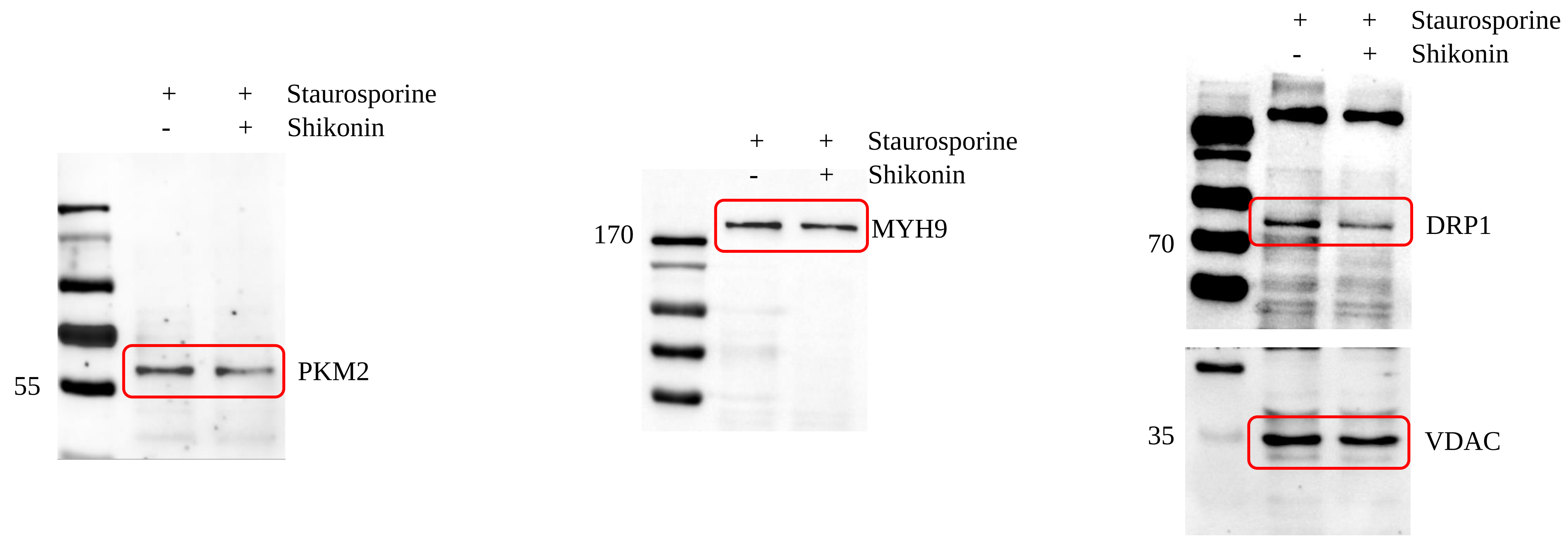
+ + Staurosporine
- + Shikonin
55
PKM2
+ + Staurosporine
- + Shikonin
170
MYH9
+ + Staurosporine
- + Shikonin
70
DRP1
35
VDAC

Figure 2 O

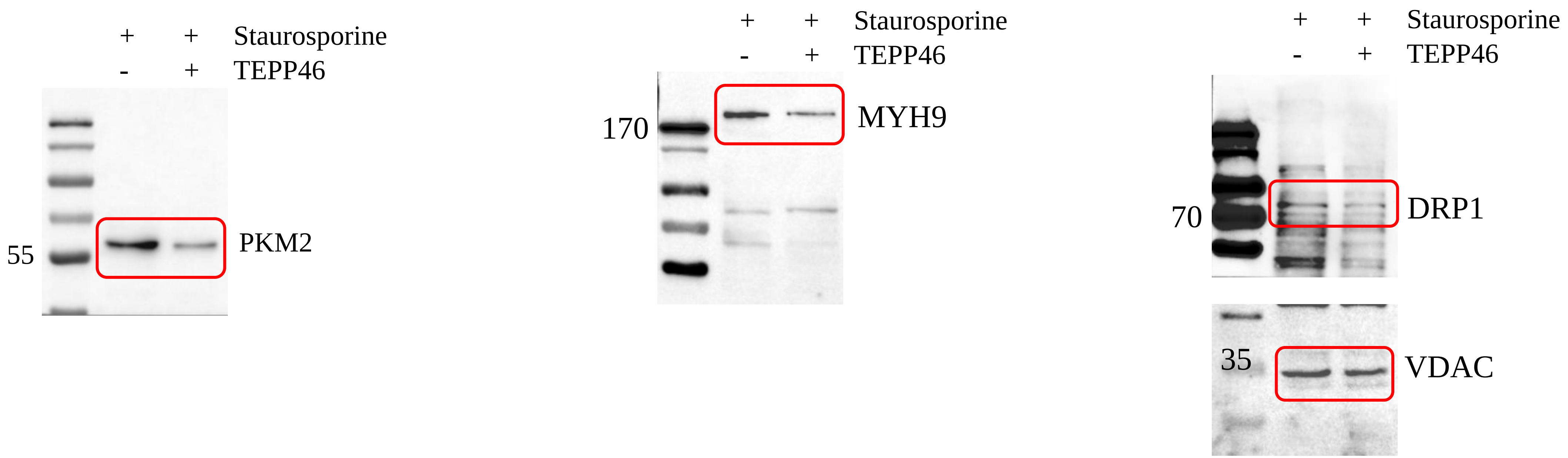
+ + Staurosporine
- + TEPP46
55
PKM2
+ + Staurosporine
- + TEPP46
170
MYH9
+ + Staurosporine
- + TEPP46
70
DRP1
35
VDAC

Figure 2 P

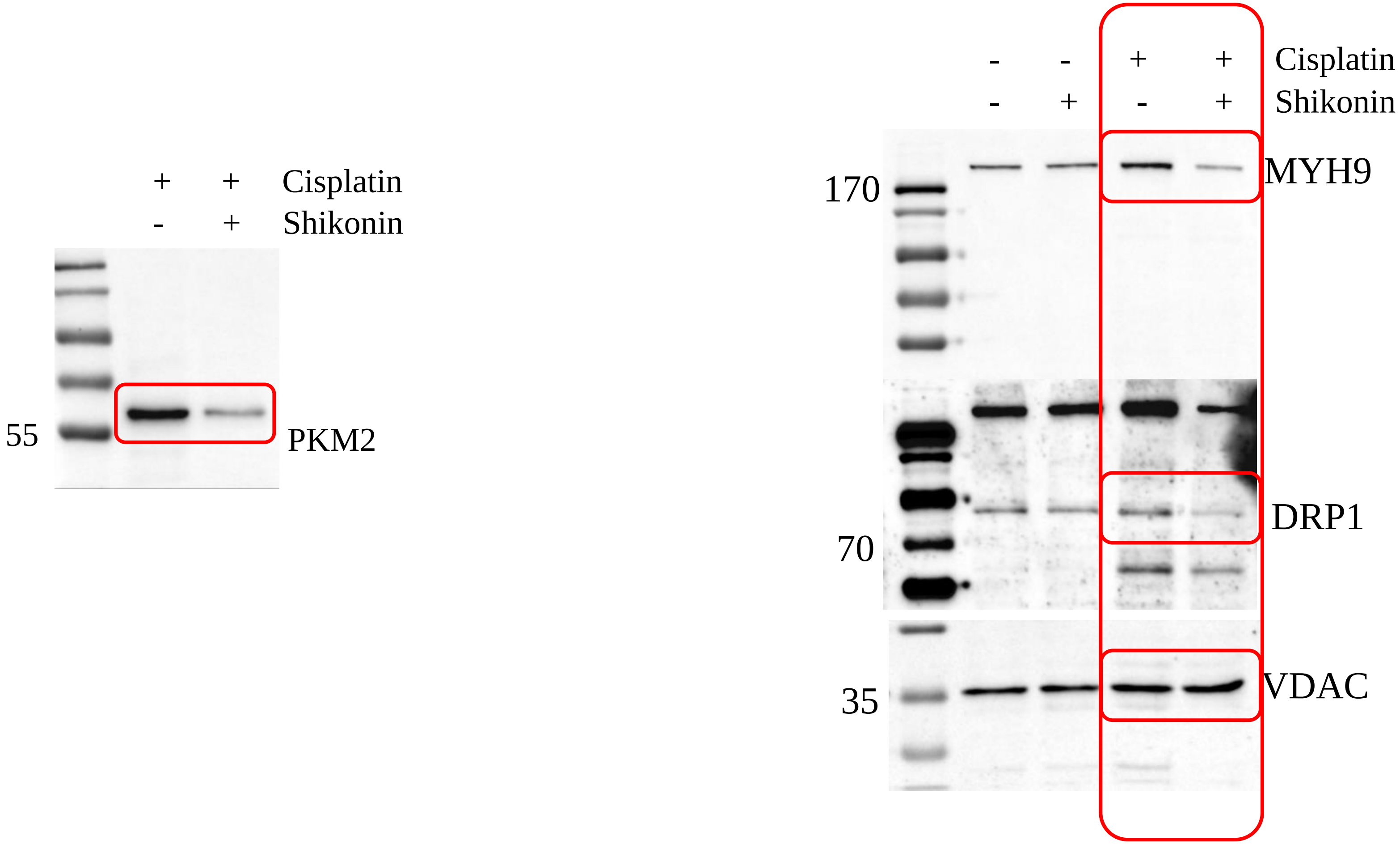
+ + Cisplatin
- + Shikonin
55
PKM2
- - + + Cisplatin
- + - + Shikonin
170
MYH9
70
DRP1
35
VDAC

Figure 2 Q

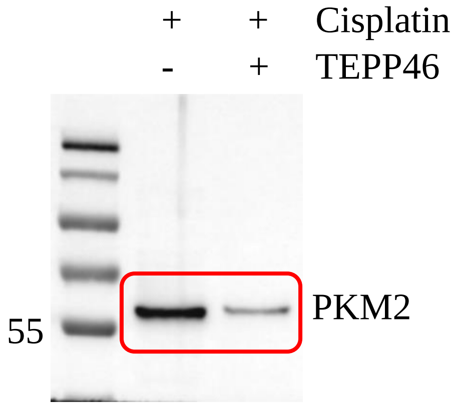
+ + Cisplatin
- + TEPP46
PKM2
55

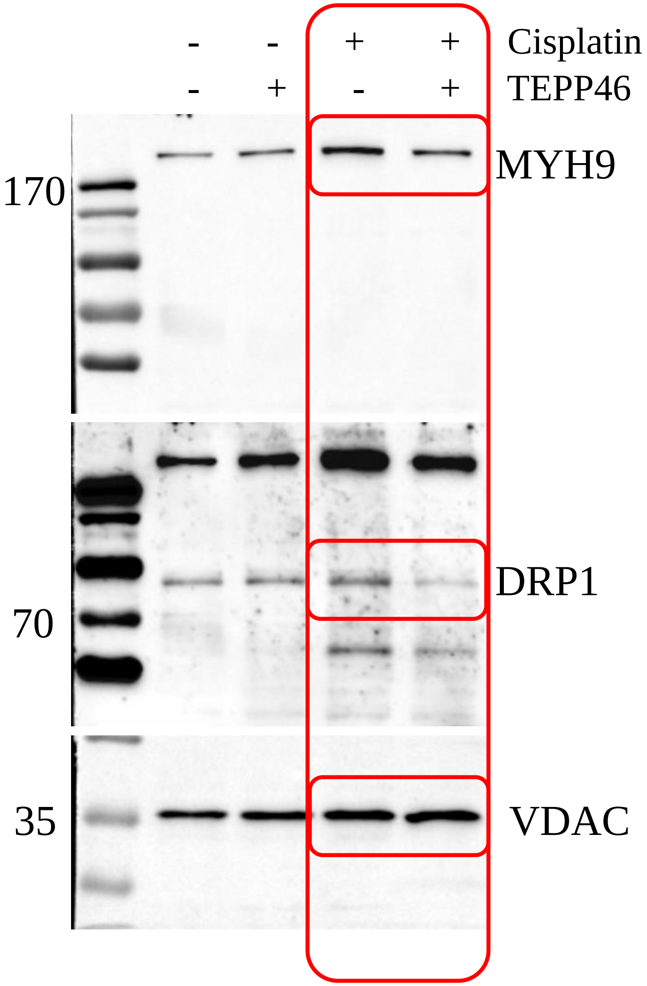
- - + + Cisplatin
- + - + TEPP46
MYH9
170
DRP1
70
VDAC
35

Figure 3 A

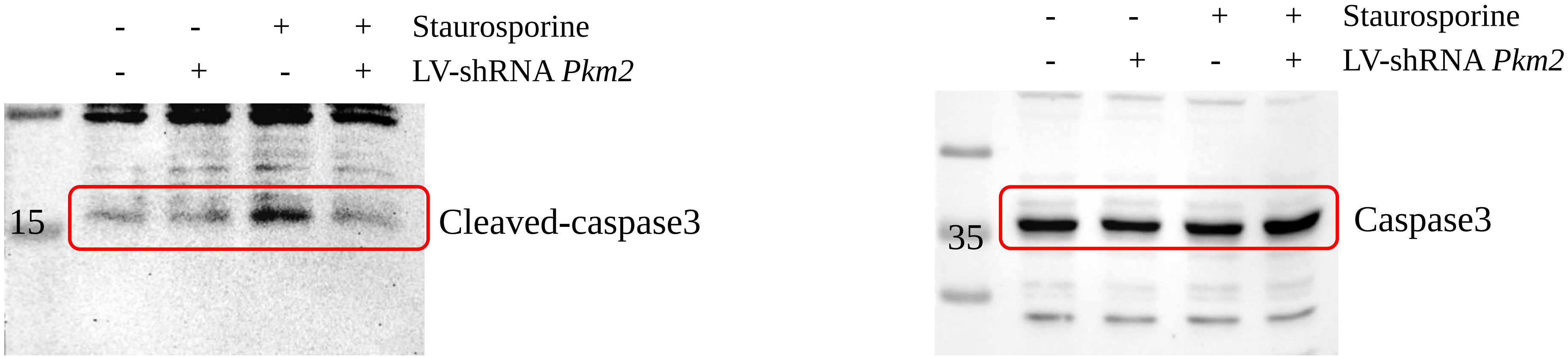

- - + + Staurosporine
- + - + LV-shRNA *Pkm2*
15
Cleaved-caspase3
- - + + Staurosporine
- + - + LV-shRNA *Pkm2*
35
Caspase3

Figure 3 A

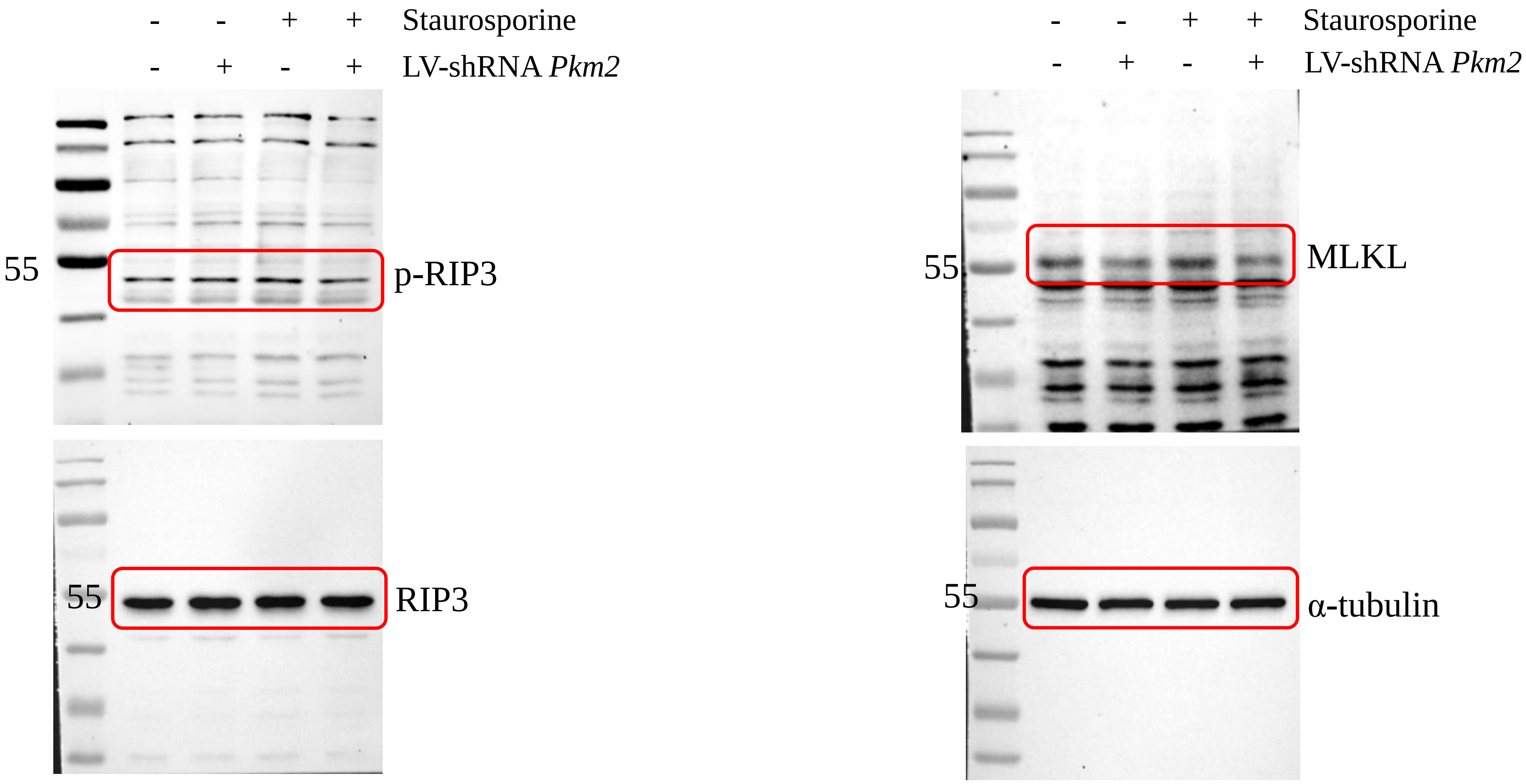

- - + + Staurosporine
- + - + LV-shRNA Pkm2
55
p-RIP3
55
RIP3
- - + + Staurosporine
- + - + LV-shRNA Pkm2
55
MLKL
55
α-tubulin

Figure 3 D

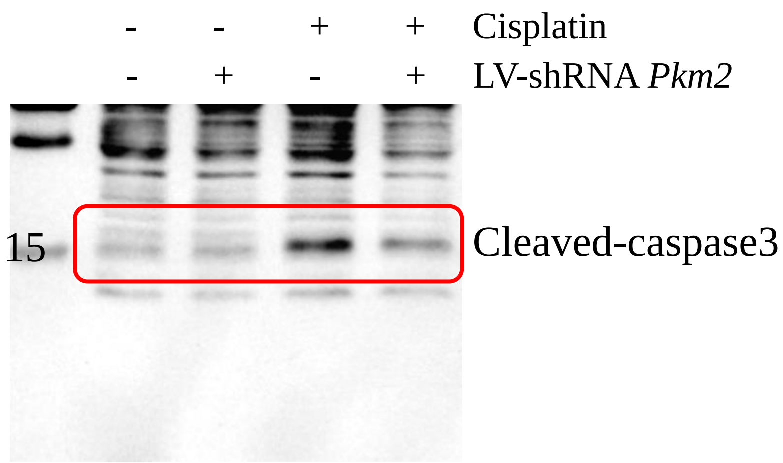

- - + + Cisplatin
- + - + LV-shRNA *Pkm2*
15
Cleaved-caspase3

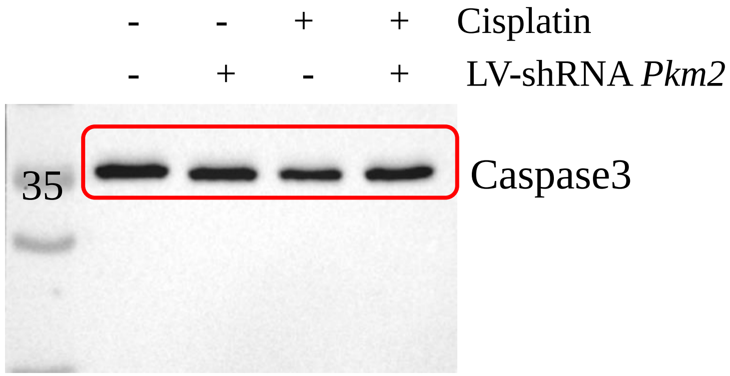

- - + + Cisplatin
- + - + LV-shRNA *Pkm2*
35
Caspase3

Figure 3 D

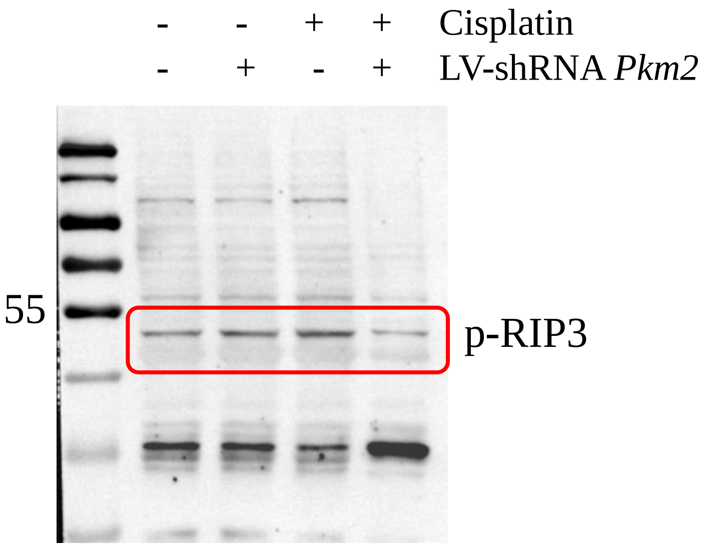

- - + + Cisplatin
- + - + LV-shRNA *Pkm2*
55
p-RIP3

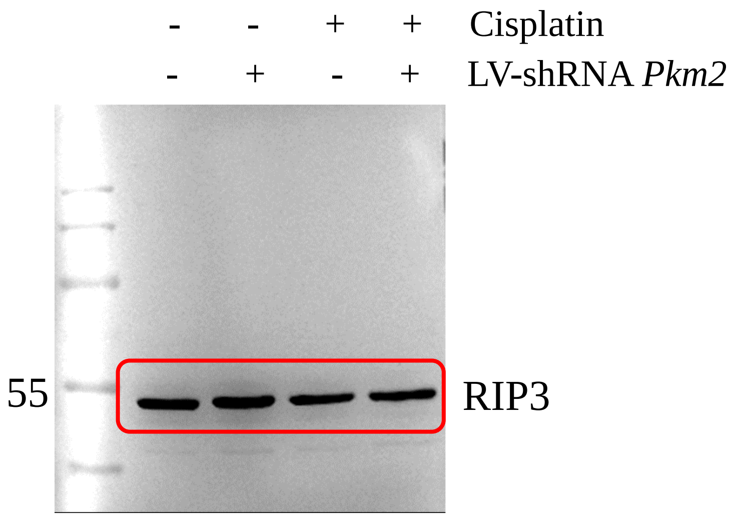

- - + + Cisplatin
- + - + LV-shRNA *Pkm2*
55
RIP3

Figure 3 D

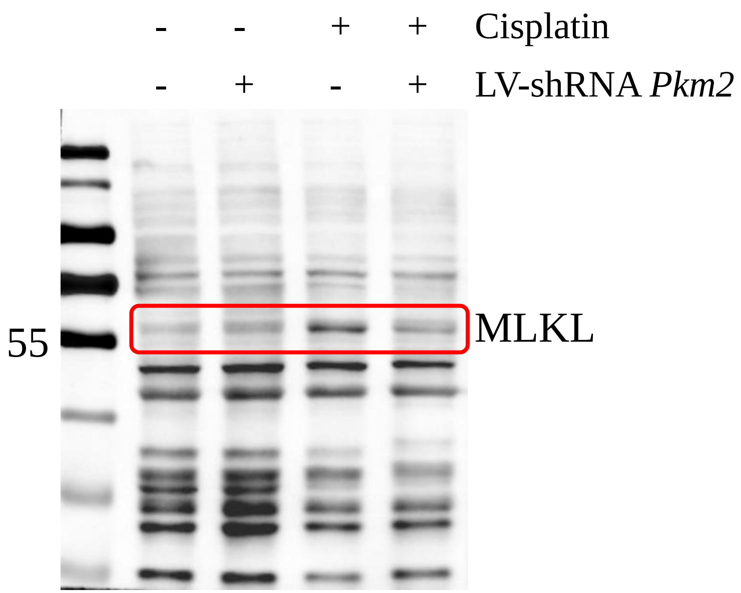
- - + + Cisplatin
- + - + LV-shRNA *Pkm2*
MLKL
55

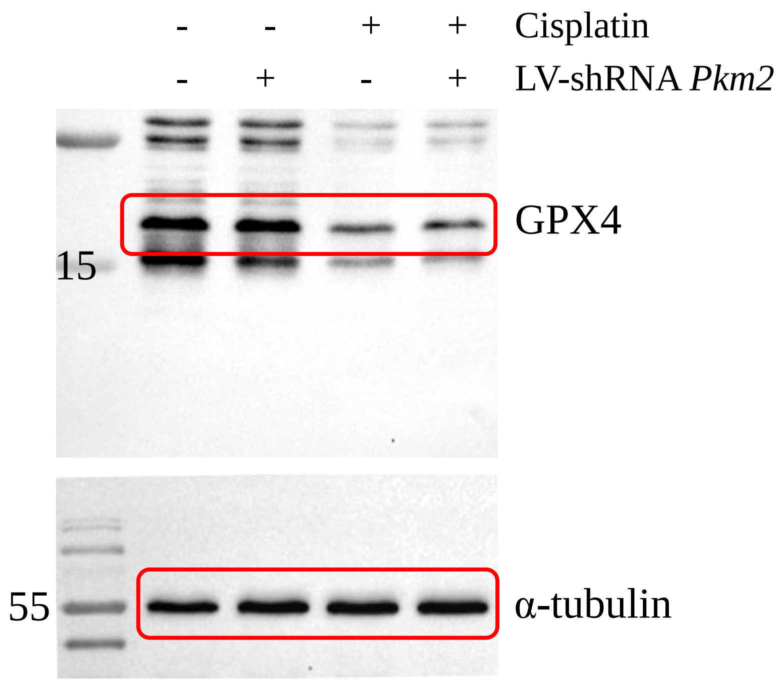
- - + + Cisplatin
- + - + LV-shRNA *Pkm2*
GPX4
15
55
α-tubulin

Figure 4 A

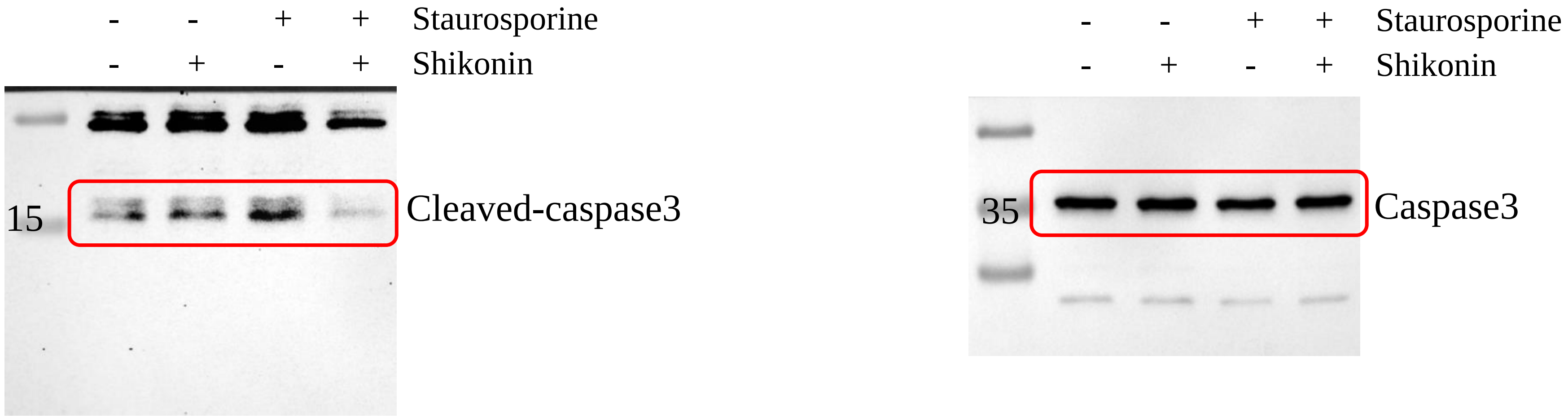

- - + + Staurosporine
- + - + Shikonin
15
Cleaved-caspase3
- - + + Staurosporine
- + - + Shikonin
35
Caspase3

Figure 4 A

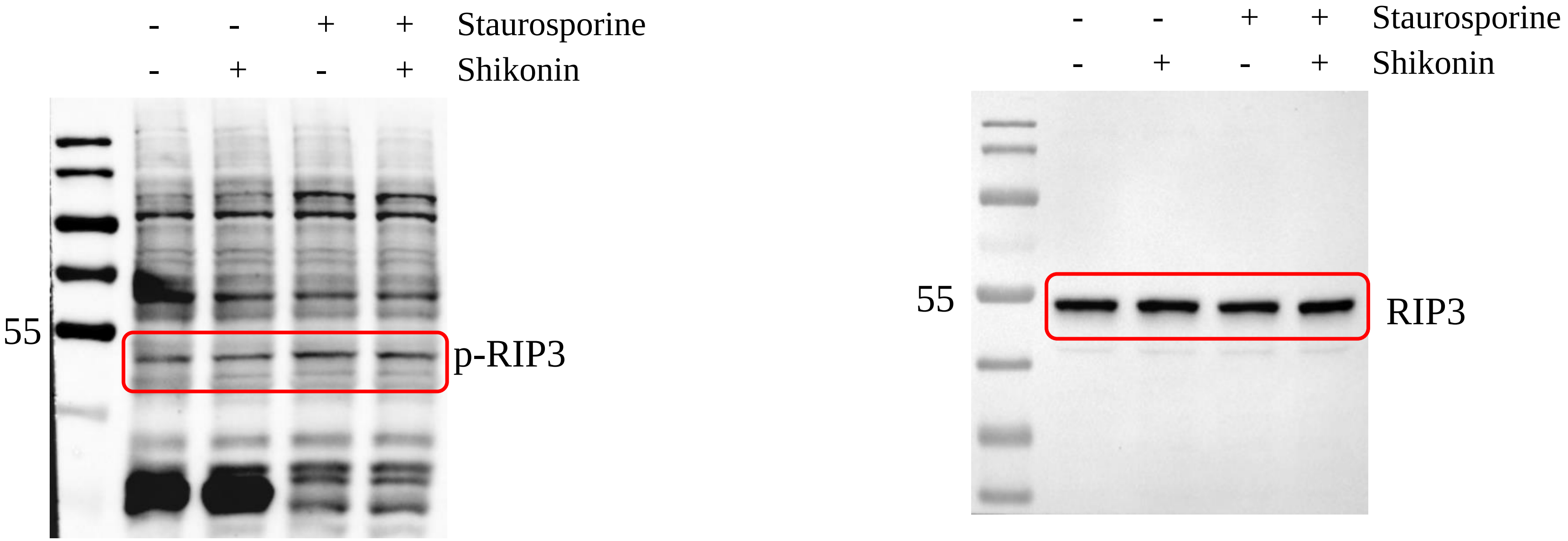

- - + + Staurosporine
- + - + Shikonin
55
p-RIP3
- - + + Staurosporine
- + - + Shikonin
55
RIP3

Figure 4 A

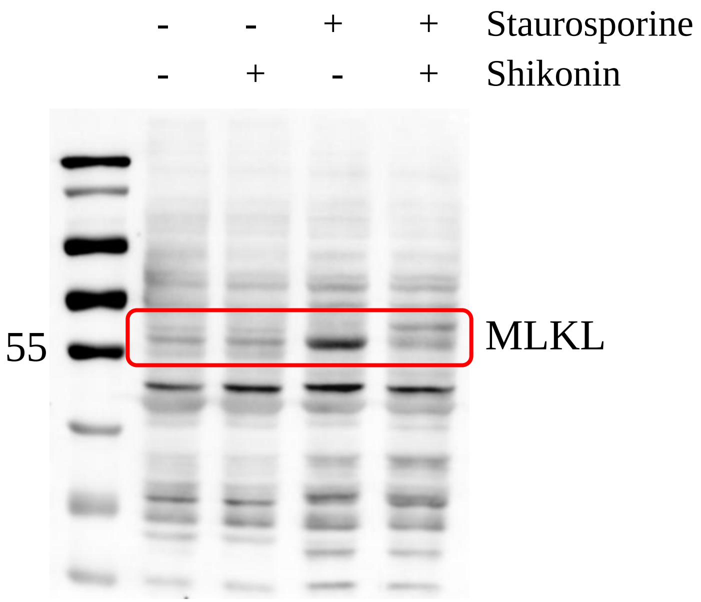

- - + + Staurosporine
- + - + Shikonin
55
MLKL

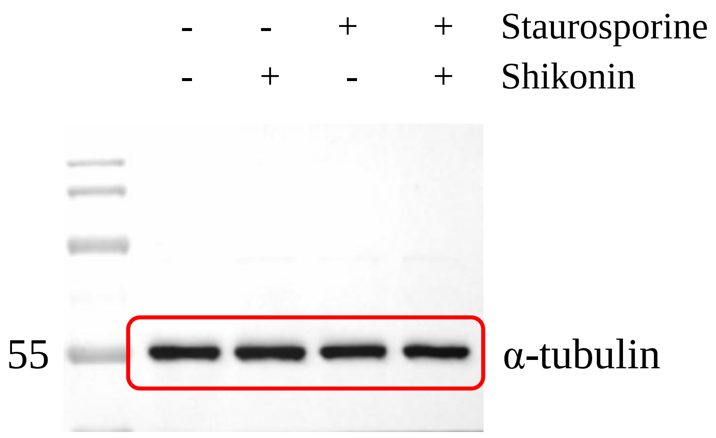

- - + + Staurosporine
- + - + Shikonin
55
α-tubulin

Figure 4 E

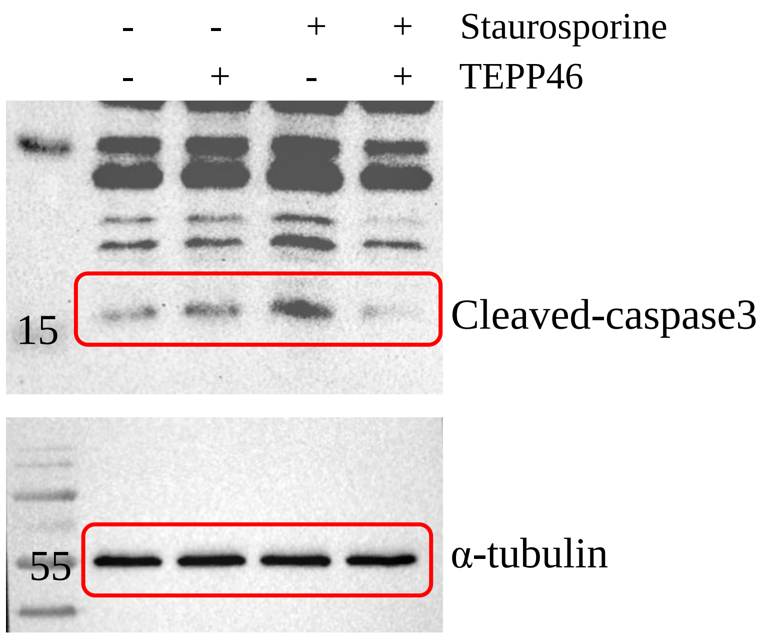

- - + + Staurosporine
- + - + TEPP46
15
Cleaved-caspase3
55
α-tubulin

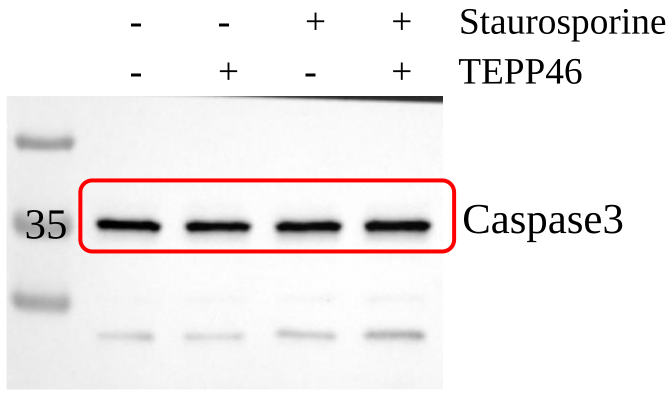

- - + + Staurosporine
- + - + TEPP46
35
Caspase3

Figure 4 E

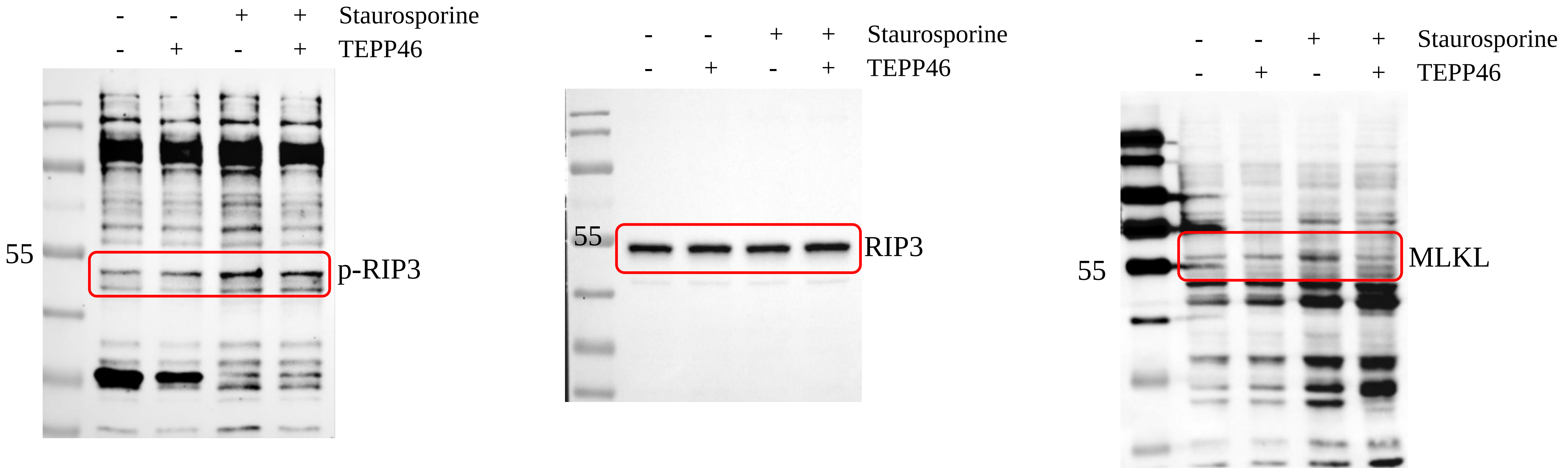
- - + + Staurosporine
- + - + TEPP46
55
p-RIP3
- - + + Staurosporine
- + - + TEPP46
55
RIP3
- - + + Staurosporine
- + - + TEPP46
55
MLKL

Figure 4 I

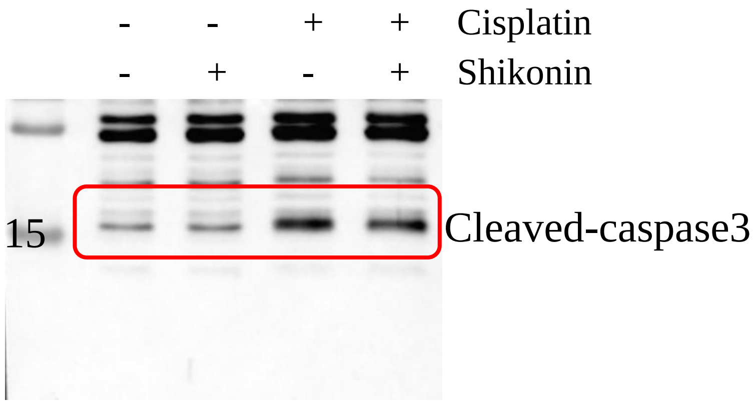

- - + + Cisplatin
- + - + Shikonin
15
Cleaved-caspase3

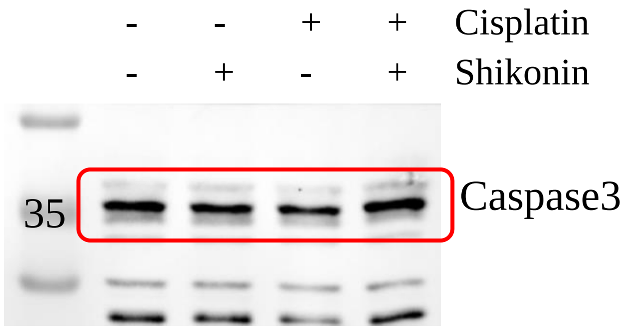

- - + + Cisplatin
- + - + Shikonin
35
Caspase3

Figure 4 I

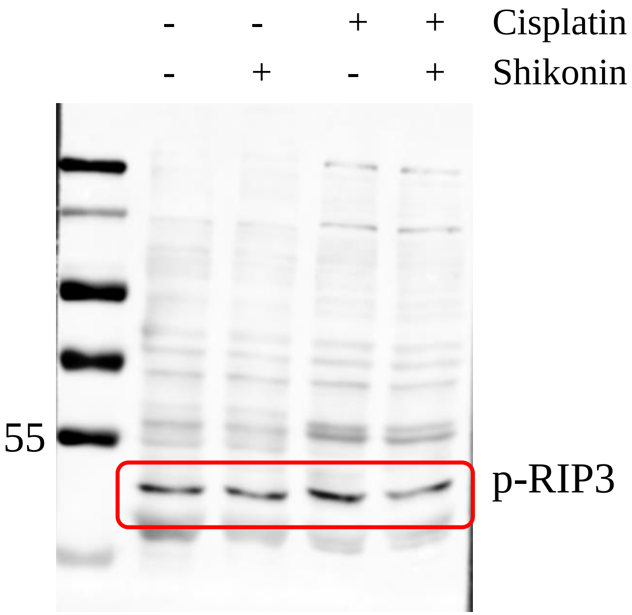

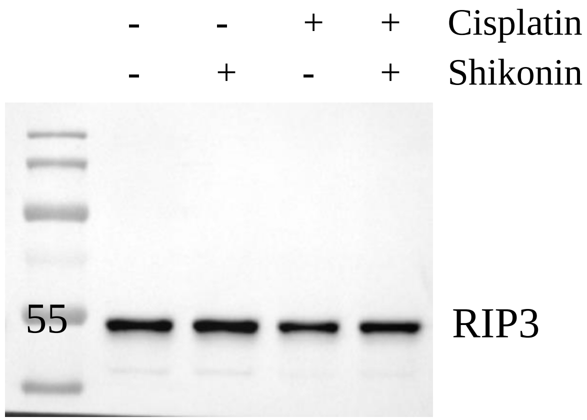

Figure 4 I

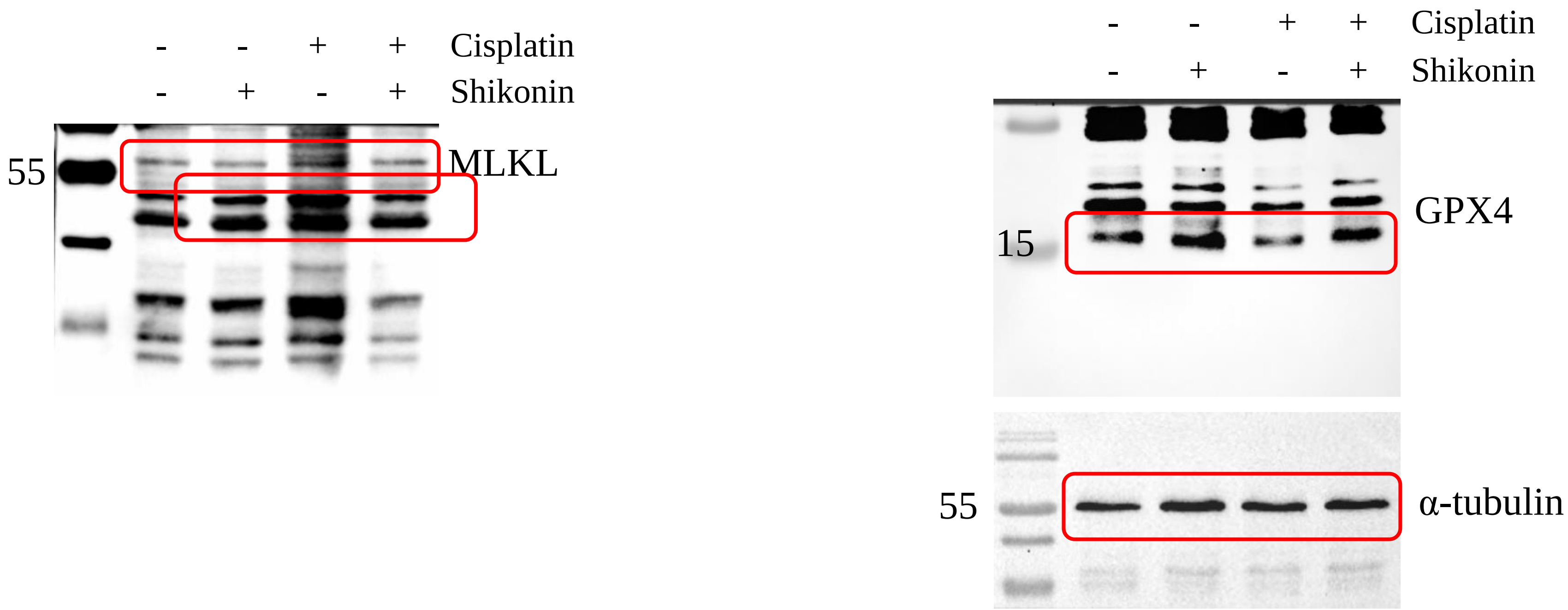

- - + + Cisplatin
- + - + Shikonin
55
MLKL
- - + + Cisplatin
- + - + Shikonin
GPX4
15
55
α-tubulin

Figure 4 M

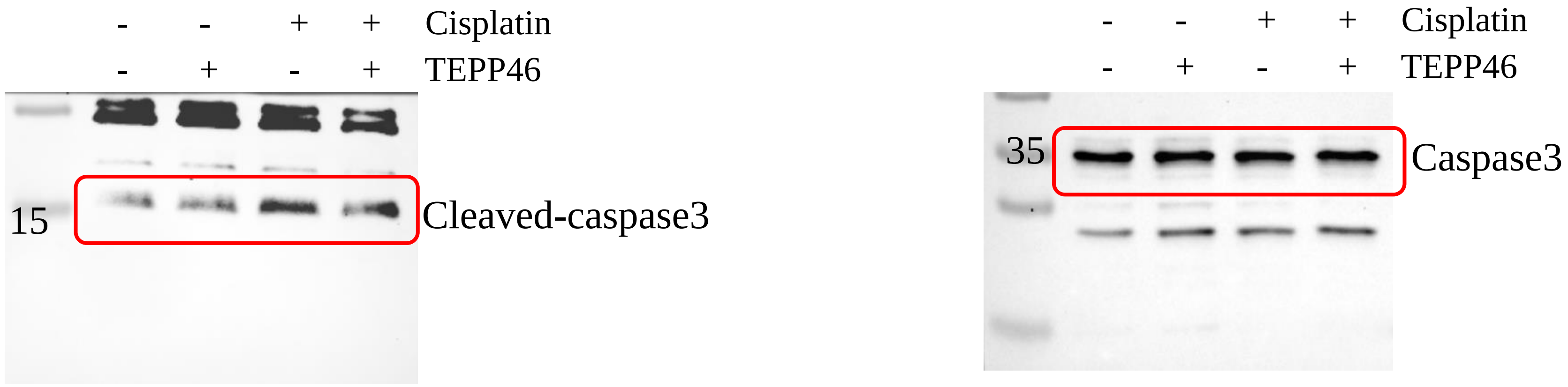
- - + + Cisplatin
- + - + TEPP46
15
Cleaved-caspase3
- - + + Cisplatin
- + - + TEPP46
35
Caspase3

Figure 4 M

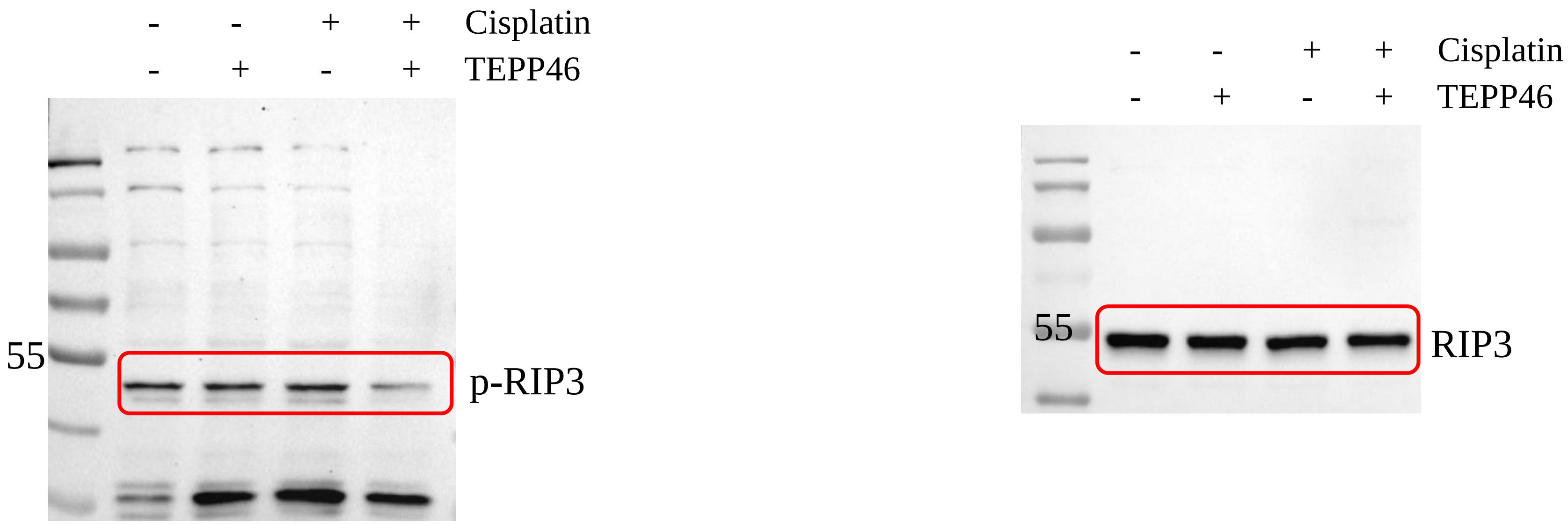

Figure 4 M

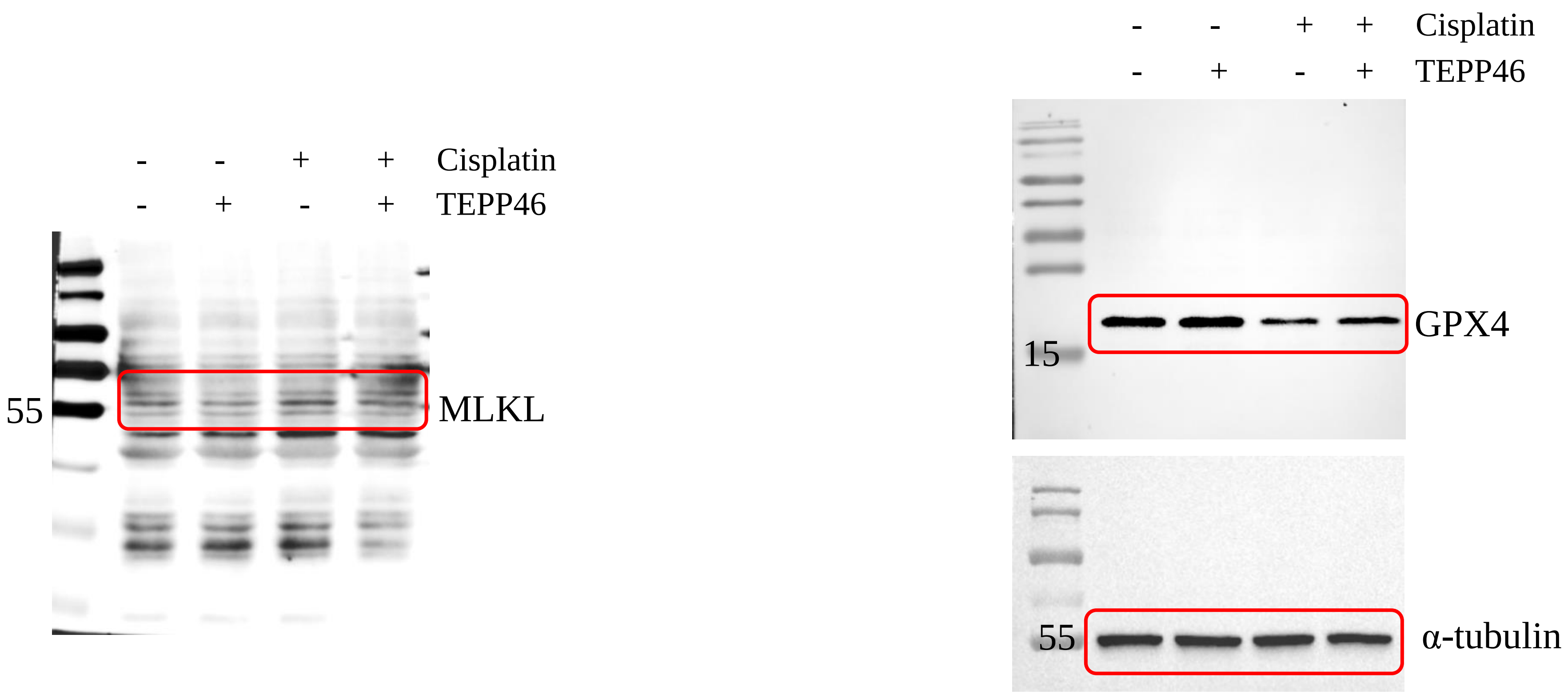

- - + + Cisplatin
- + - + TEPP46
55
MLKL
- - + + Cisplatin
- + - + TEPP46
15
GPX4
55
α-tubulin

Figure 5 A

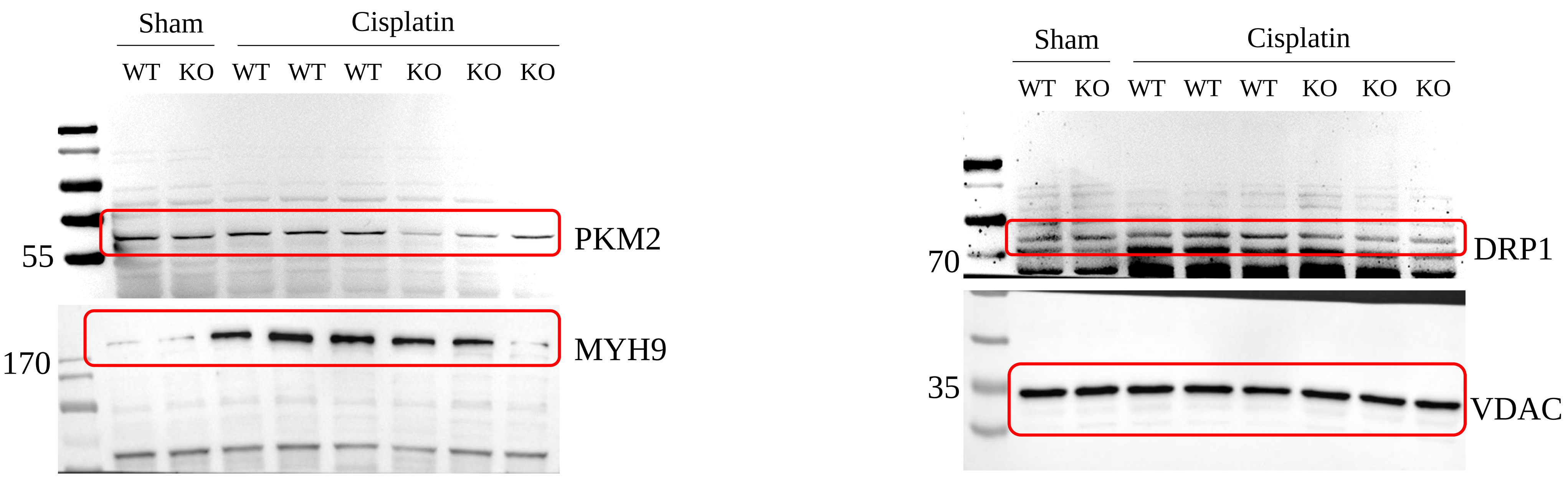

Sham
Cisplatin
WT KO WT WT WT KO KO KO
55
PKM2
170
MYH9
Sham
Cisplatin
WT KO WT WT WT KO KO KO
70
DRP1
35
VDAC

Figure 5 H

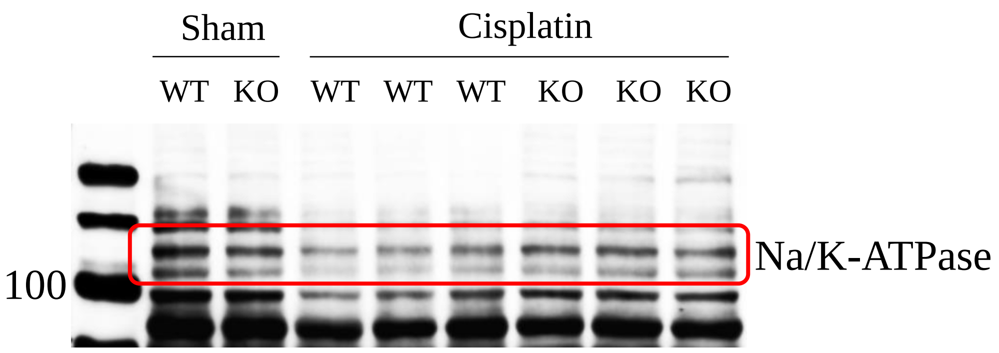
Sham
Cisplatin
WT KO WT WT WT KO KO KO
100
Na/K-ATPase

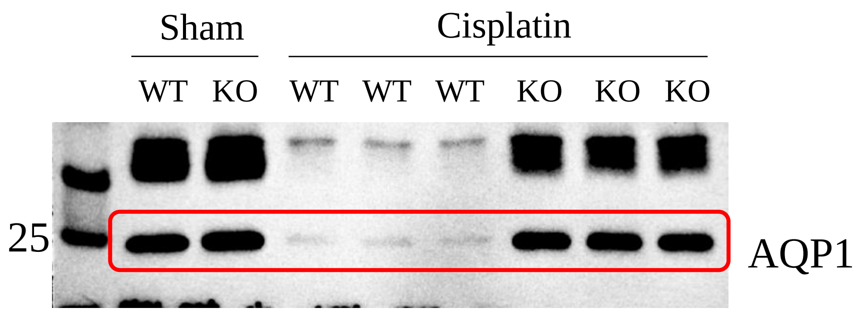
Sham
Cisplatin
WT KO WT WT WT KO KO KO
25
AQP1

Figure 5 H

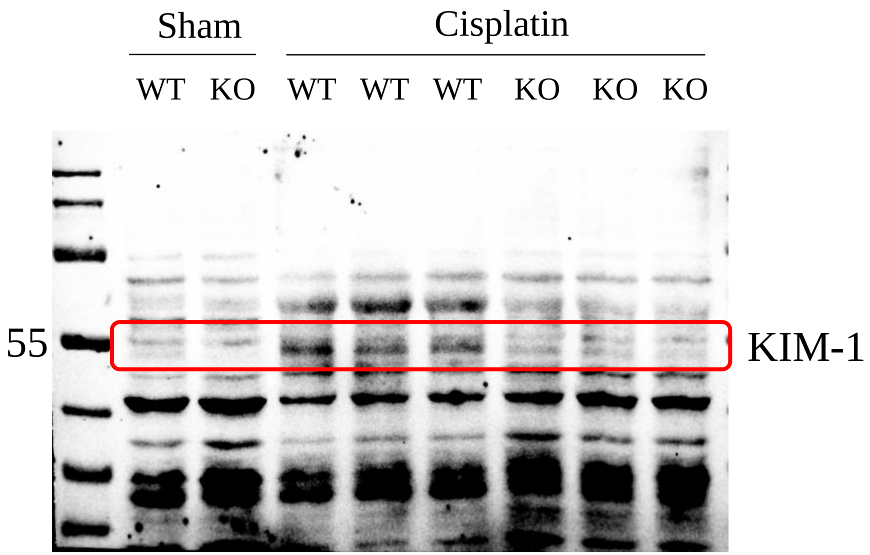

Figure 5 H

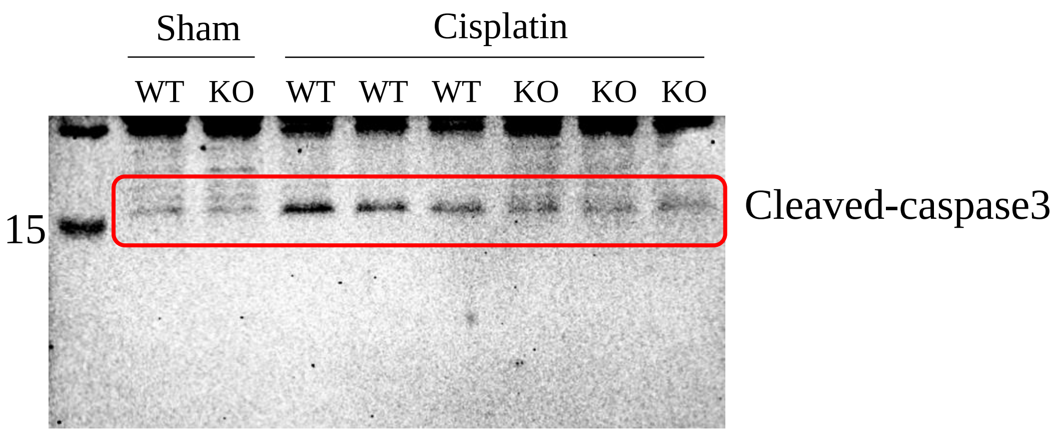

Sham
Cisplatin
WT KO WT WT WT KO KO KO
15
Cleaved-caspase3

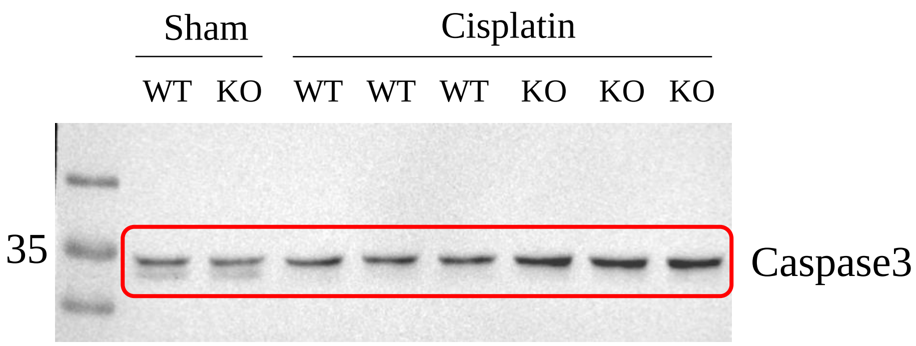

Sham
Cisplatin
WT KO WT WT WT KO KO KO
35
Caspase3

Figure 5 H

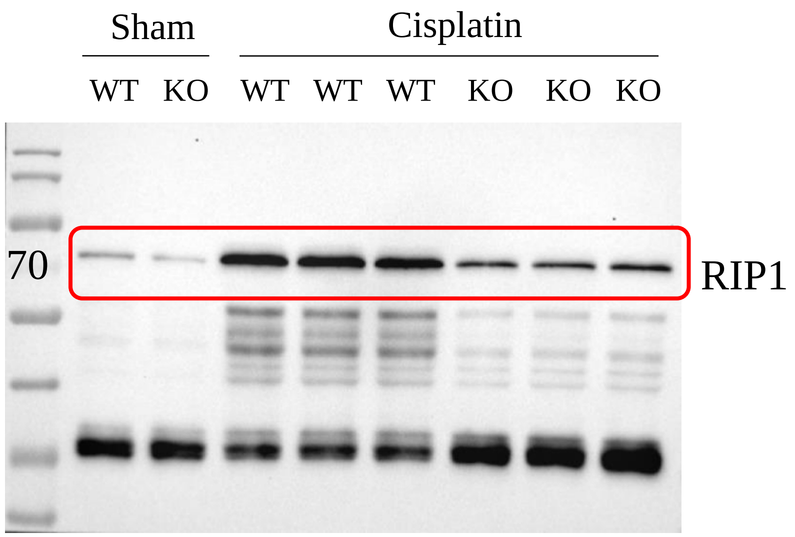

Sham
Cisplatin
WT KO WT WT WT KO KO KO
70
RIP1

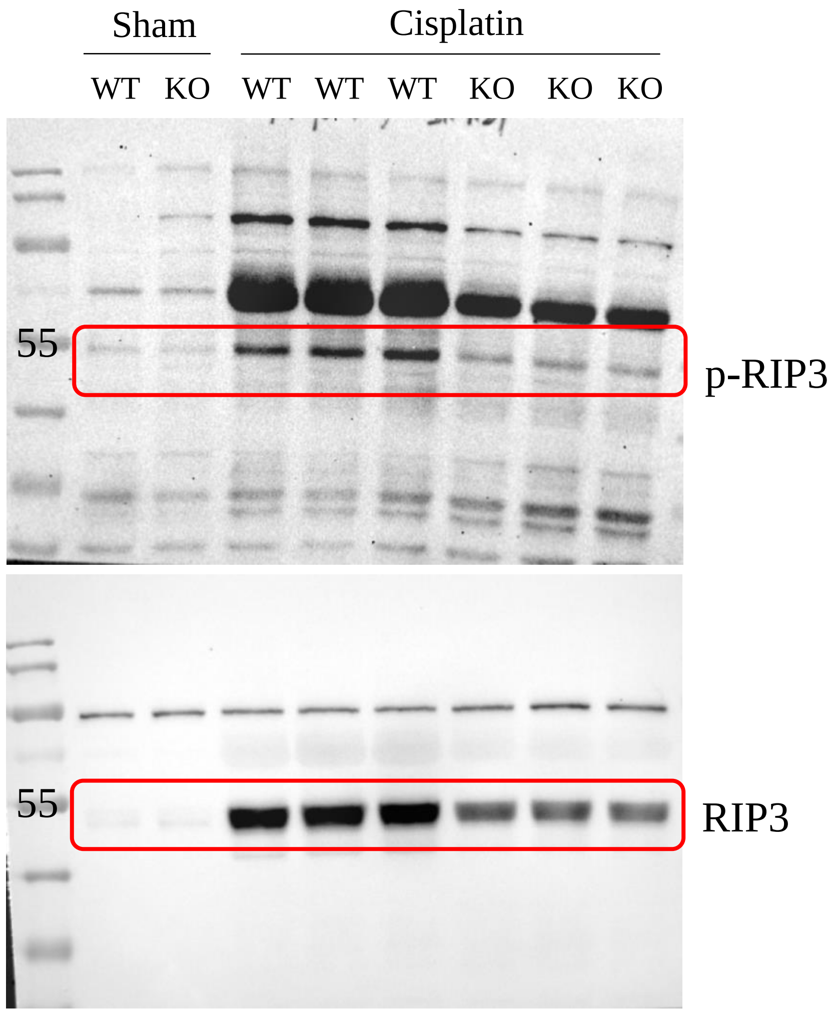

Sham
Cisplatin
WT KO WT WT WT KO KO KO
55
p-RIP3
55
RIP3

Figure 5 H

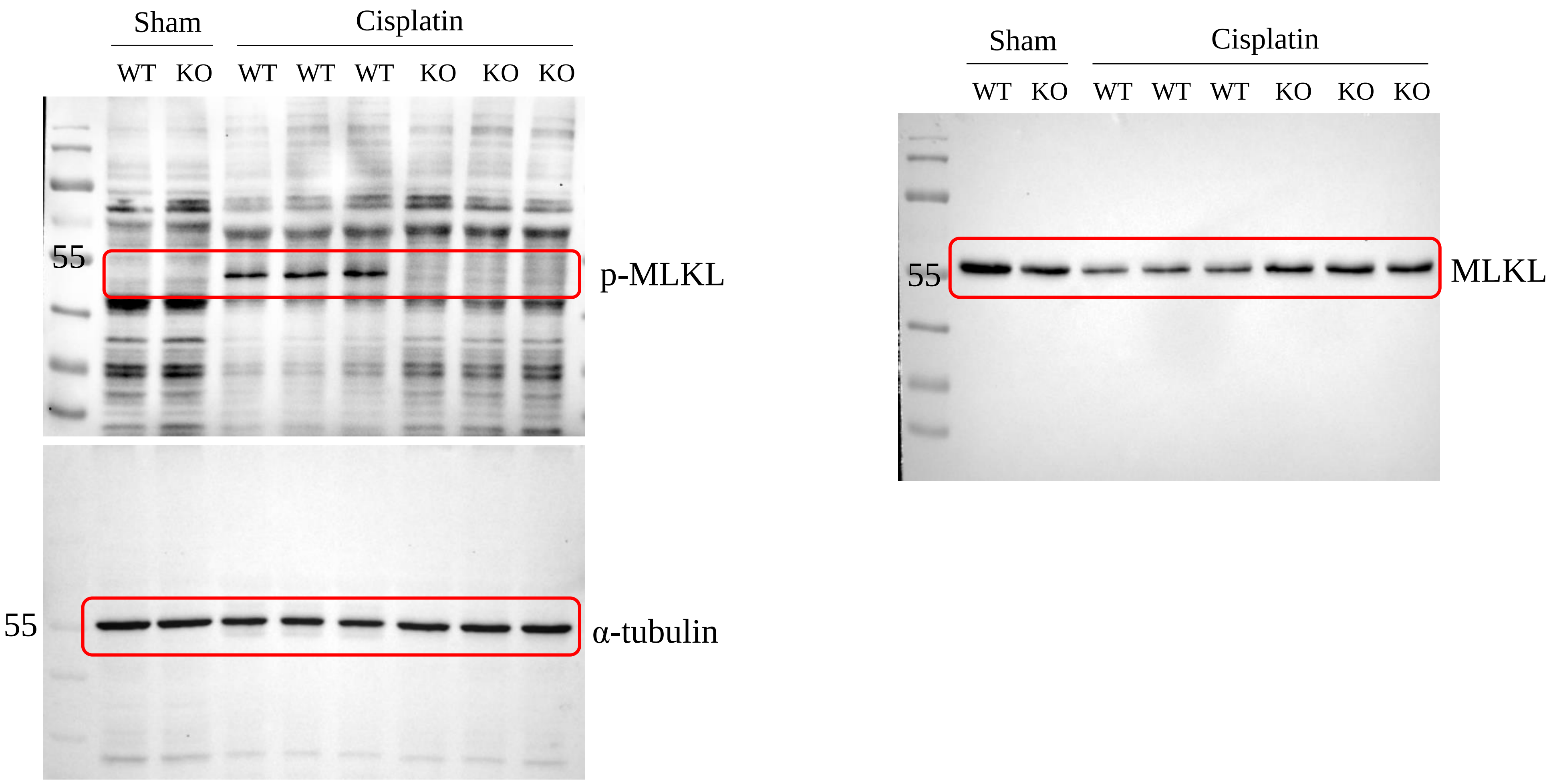
Sham
Cisplatin
WT KO WT WT WT KO KO KO
55
p-MLKL
55
α-tubulin
Sham
Cisplatin
WT KO WT WT WT KO KO KO
55
MLKL

Figure 5 H

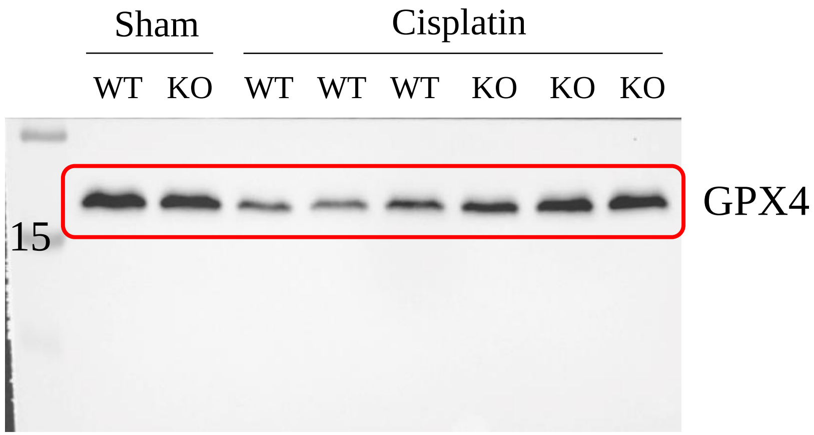

Figure 6 A

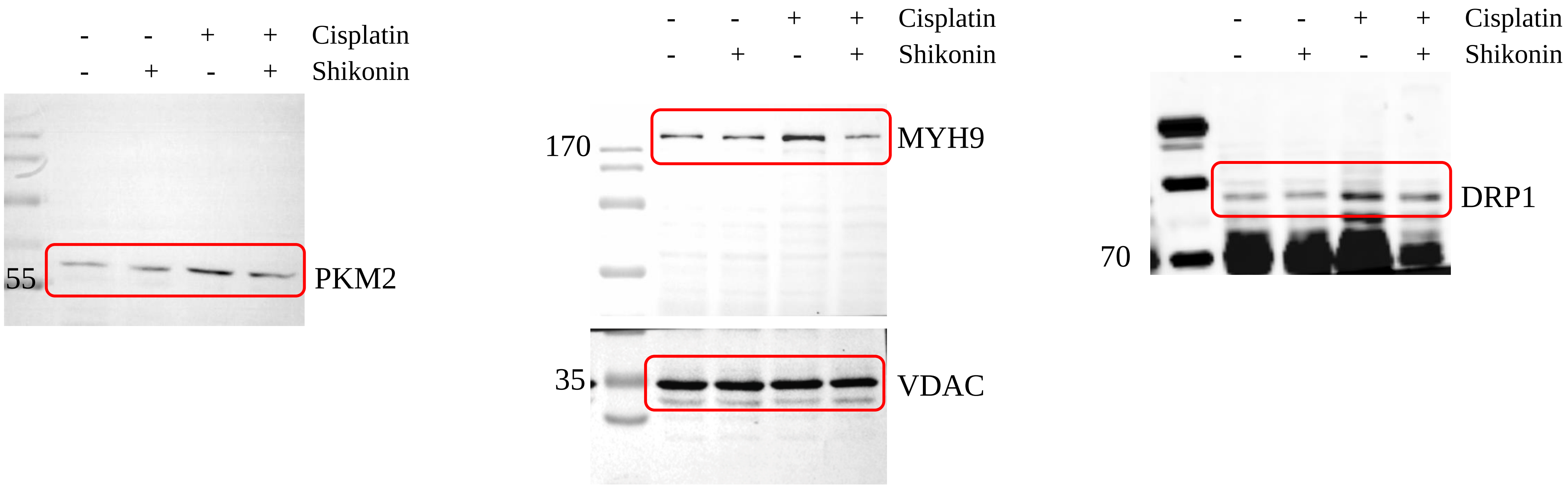

- - + + Cisplatin
- + - + Shikonin
55
PKM2
- - + + Cisplatin
- + - + Shikonin
170
MYH9
35
VDAC
- - + + Cisplatin
- + - + Shikonin
DRP1
70

Figure 6 B

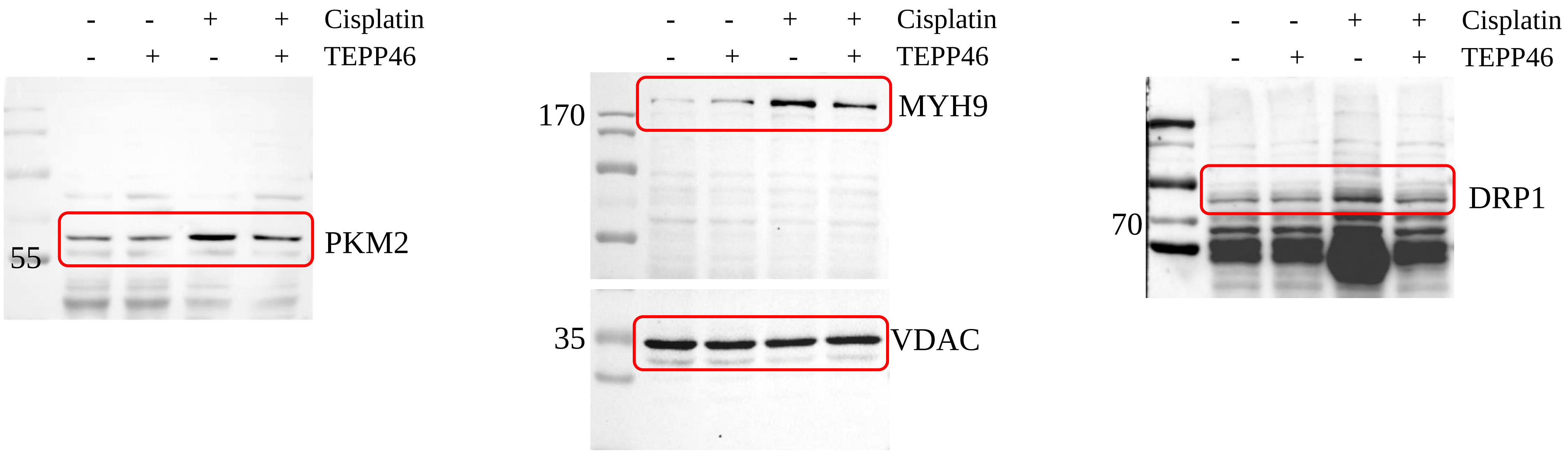

- - + + Cisplatin
- + - + TEPP46
55
PKM2
- - + + Cisplatin
- + - + TEPP46
170
MYH9
35
VDAC
- - + + Cisplatin
- + - + TEPP46
70
DRP1

Figure 6 I

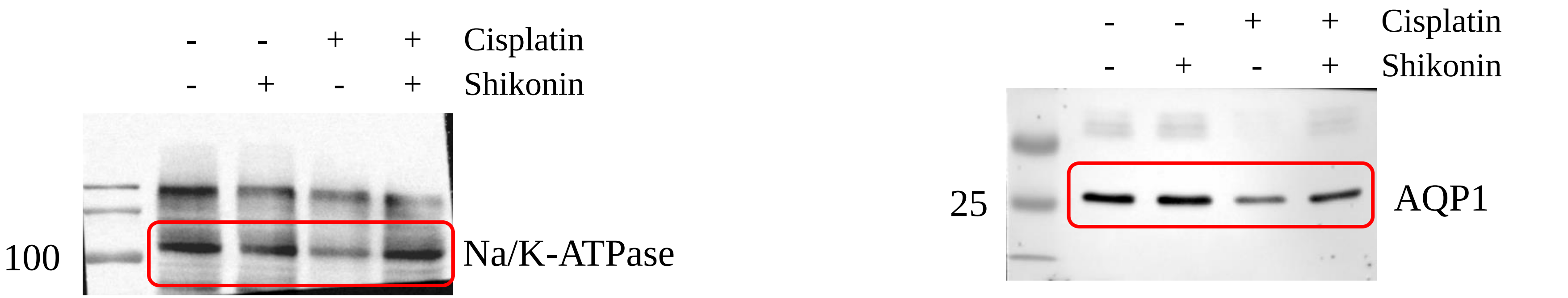
- - + + Cisplatin
- + - + Shikonin
100
Na/K-ATPase
- - + + Cisplatin
- + - + Shikonin
25
AQP1

Figure 6 I

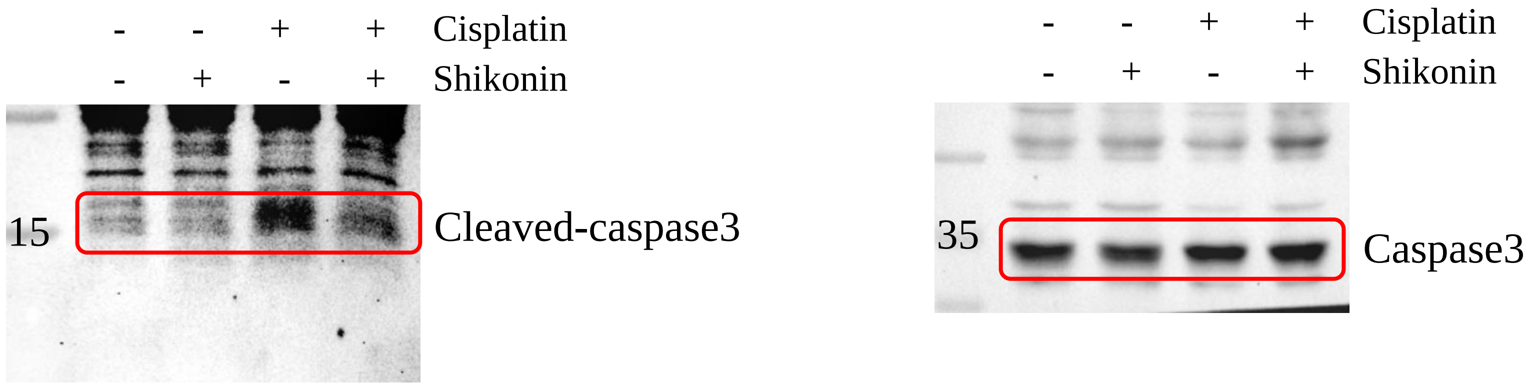

- - + + Cisplatin
- + - + Shikonin
15
Cleaved-caspase3
- - + + Cisplatin
- + - + Shikonin
35
Caspase3

Figure 6 I

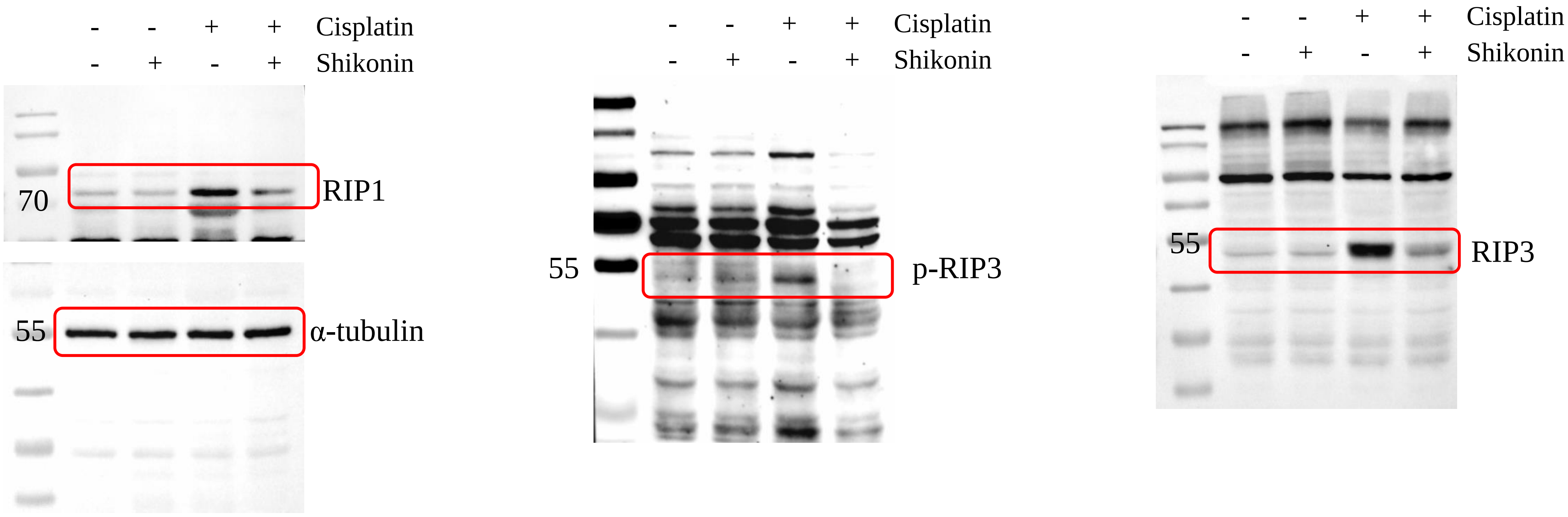
- - + + Cisplatin
- + - + Shikonin
70
RIP1
55
α-tubulin
- - + + Cisplatin
- + - + Shikonin
55
p-RIP3
- - + + Cisplatin
- + - + Shikonin
55
RIP3

Figure 6 I

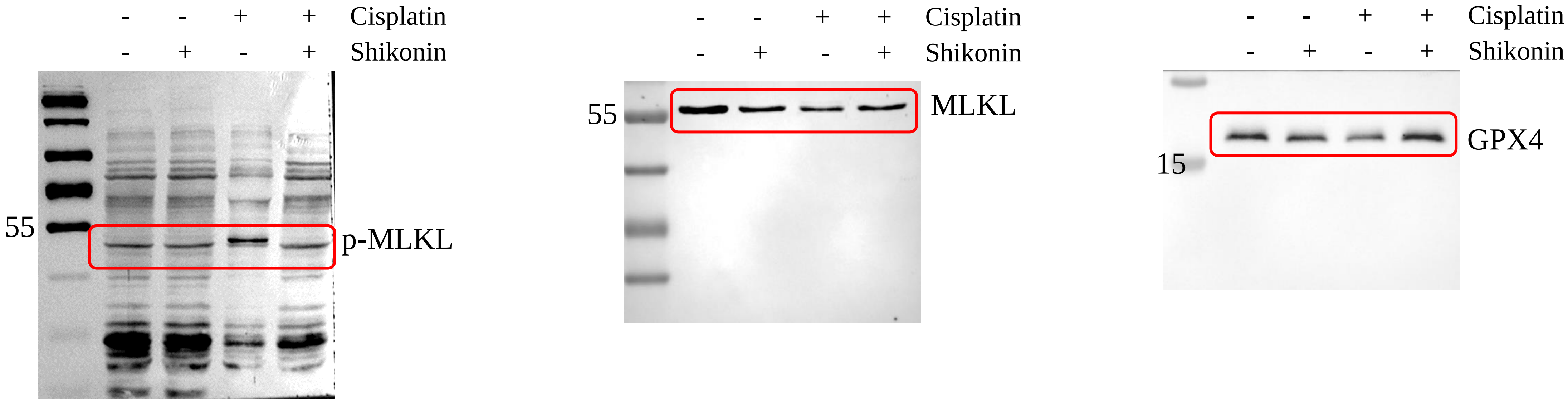

- - + + Cisplatin
- + - + Shikonin
55
p-MLKL
- - + + Cisplatin
- + - + Shikonin
55
MLKL
- - + + Cisplatin
- + - + Shikonin
15
GPX4

Figure 6 J

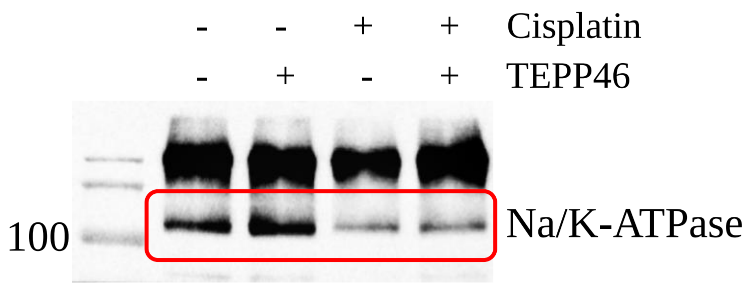

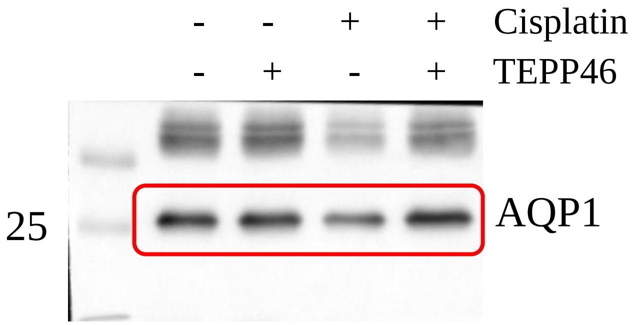

Figure 6 J

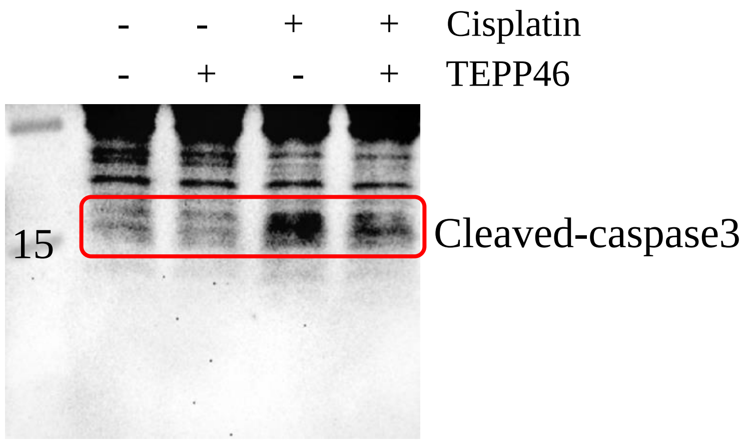

- - + + Cisplatin
- + - + TEPP46
15
Cleaved-caspase3

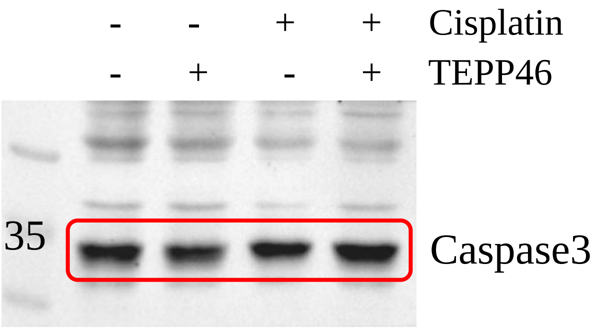

- - + + Cisplatin
- + - + TEPP46
35
Caspase3

Figure 6 J

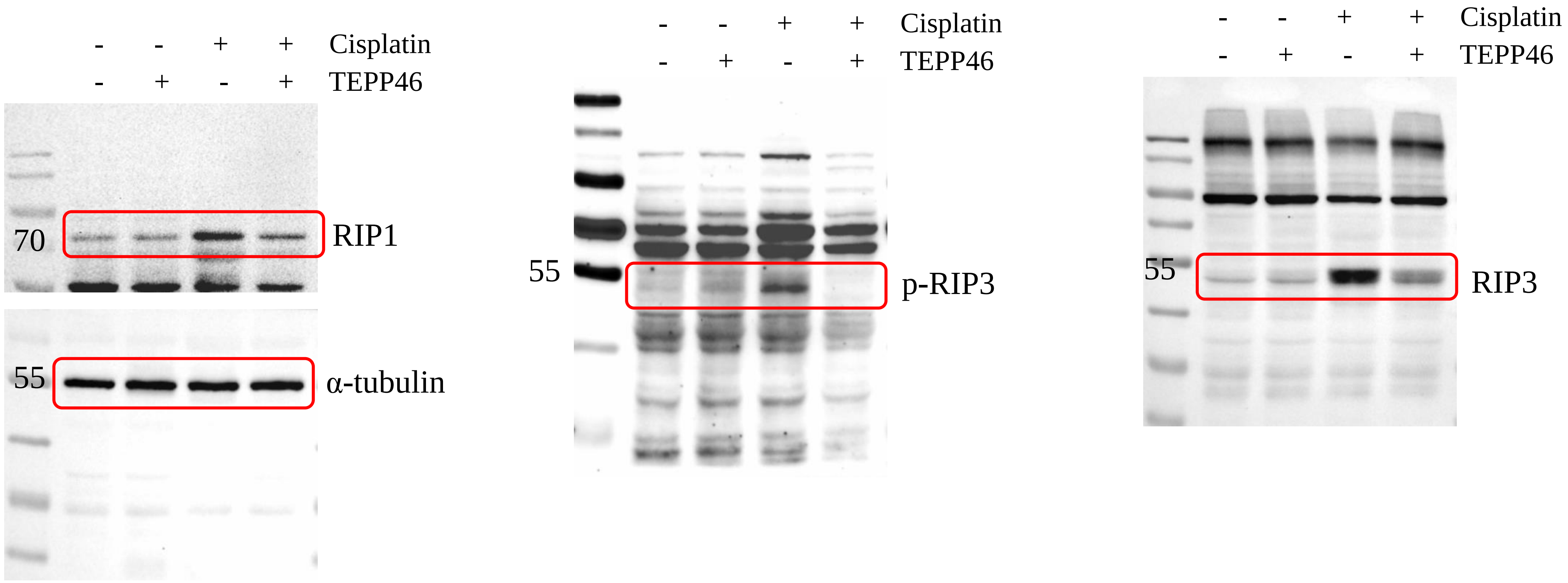

- - + + Cisplatin
- + - + TEPP46
70
RIP1
55
α-tubulin
- - + + Cisplatin
- + - + TEPP46
55
p-RIP3
- - + + Cisplatin
- + - + TEPP46
55
RIP3

Figure 6 J

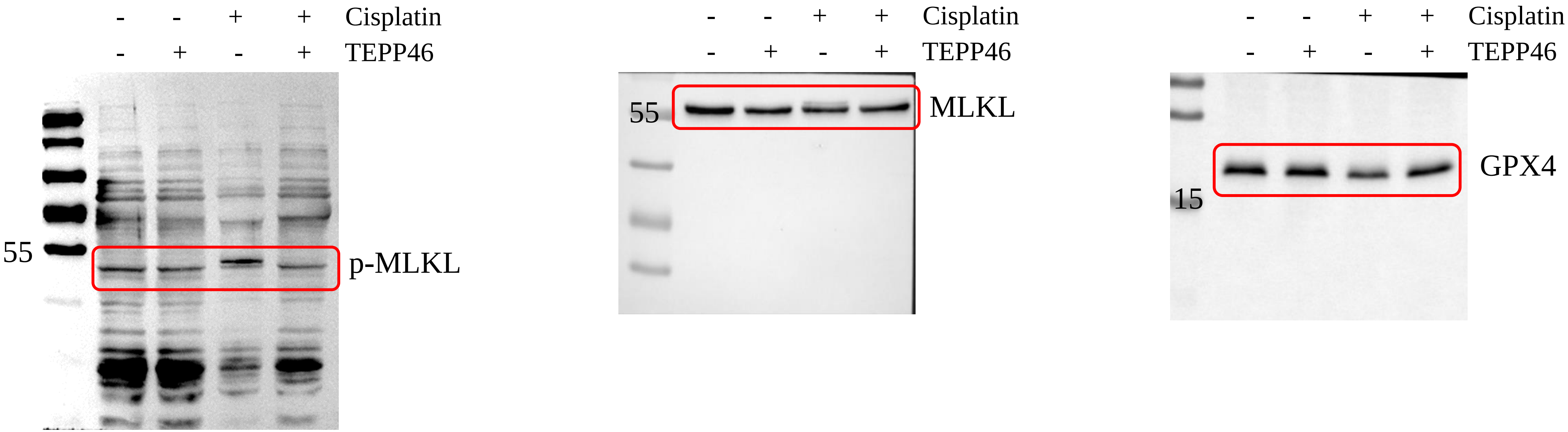

- - + + Cisplatin
- + - + TEPP46
55
p-MLKL
- - + + Cisplatin
- + - + TEPP46
55
MLKL
- - + + Cisplatin
- + - + TEPP46
15
GPX4

Supplementary Figure 1 A-B

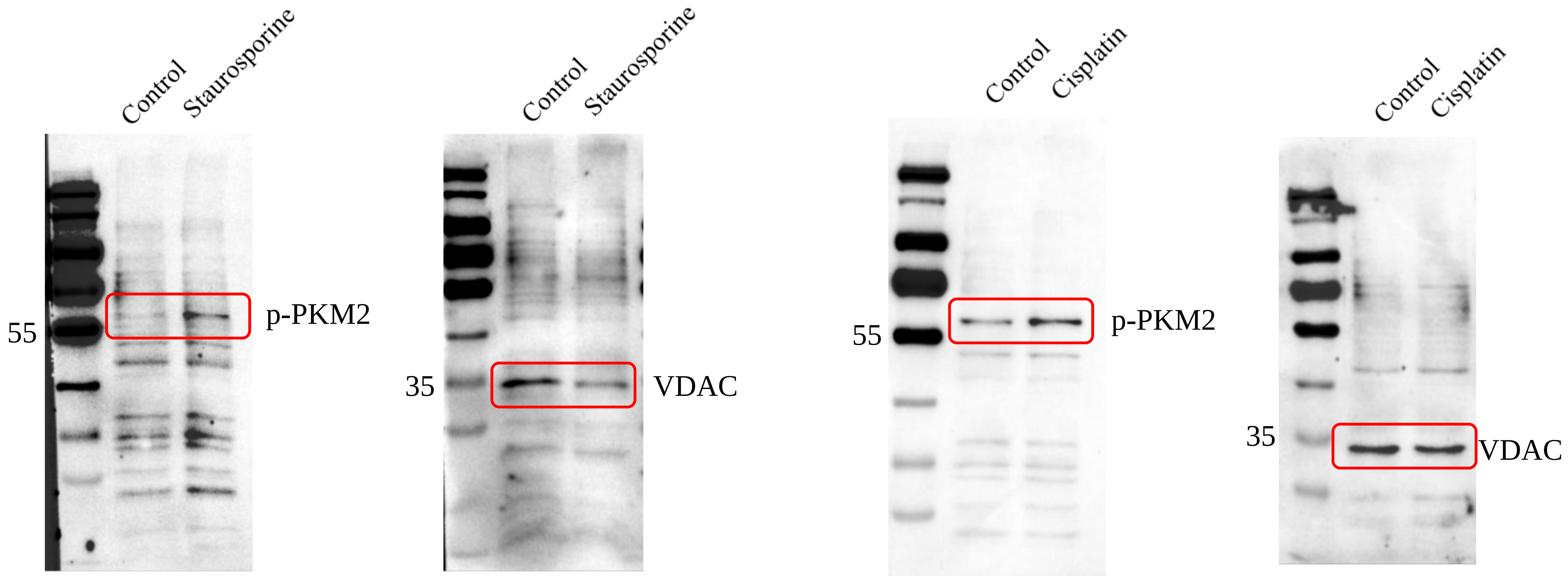

Supplementary Figure 1 C

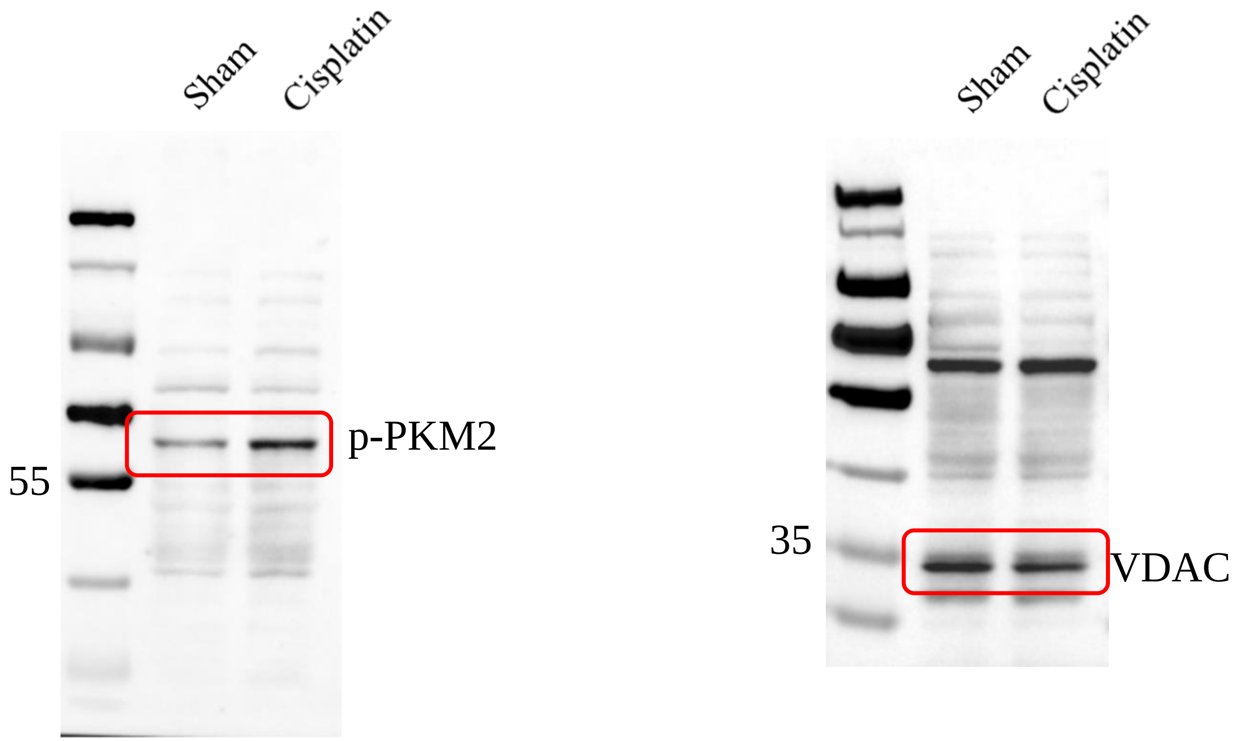

Supplementary Figure 1 D

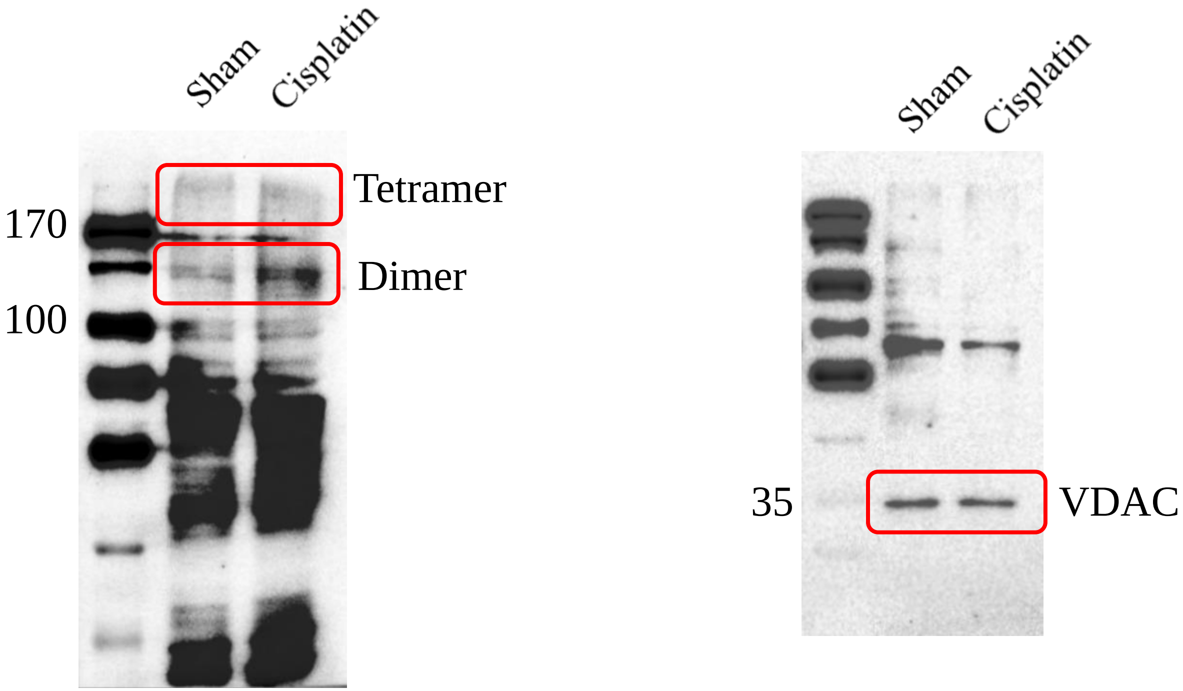

Supplementary Figure 2 A

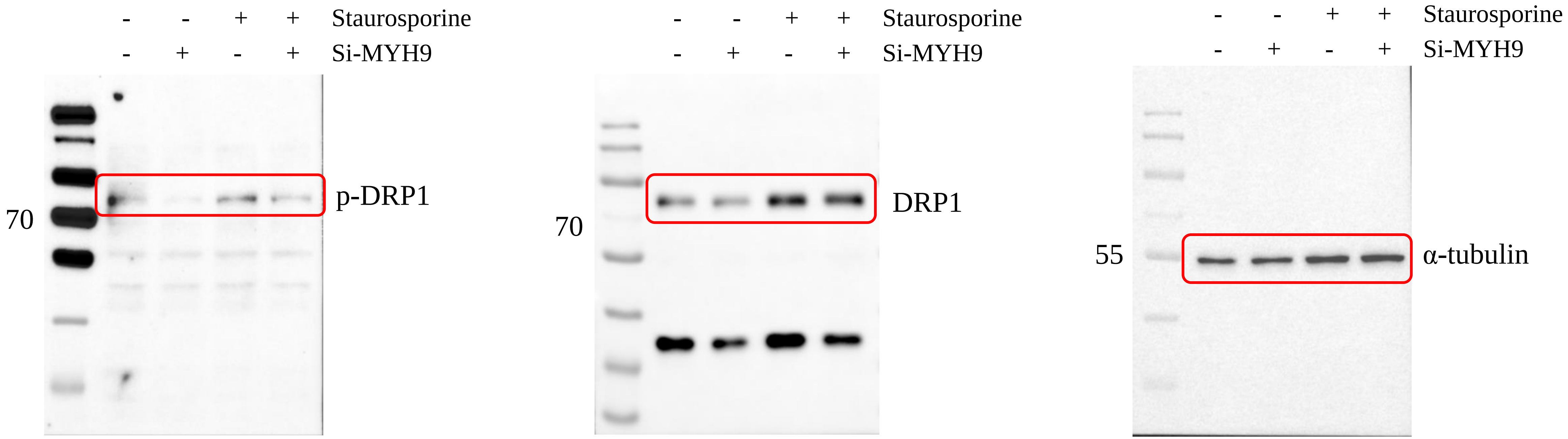

Supplementary Figure 2 D

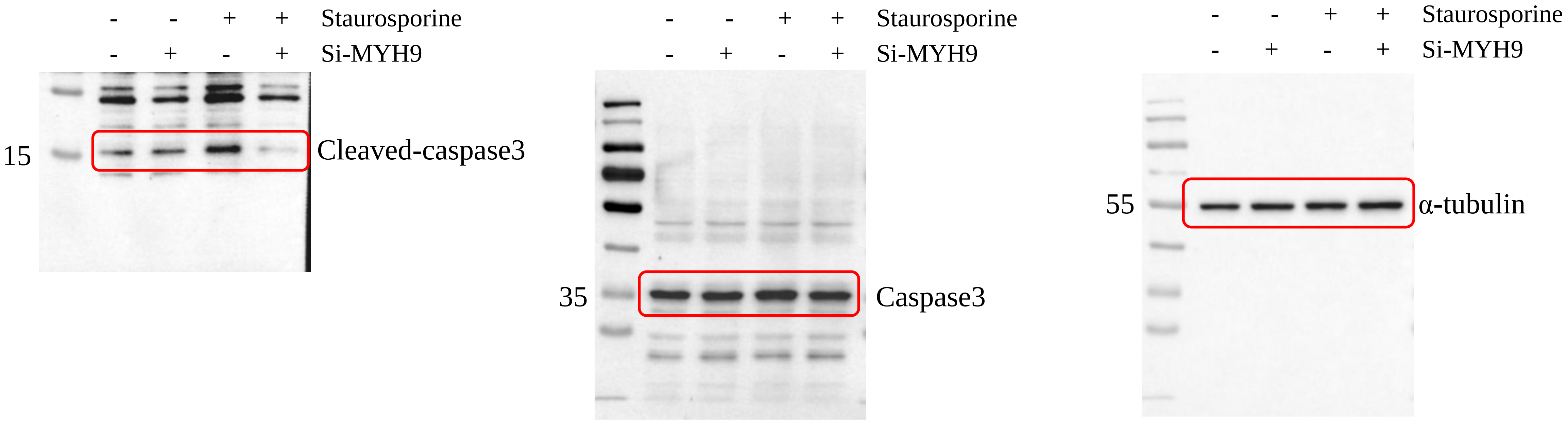

Supplementary Figure 2 D

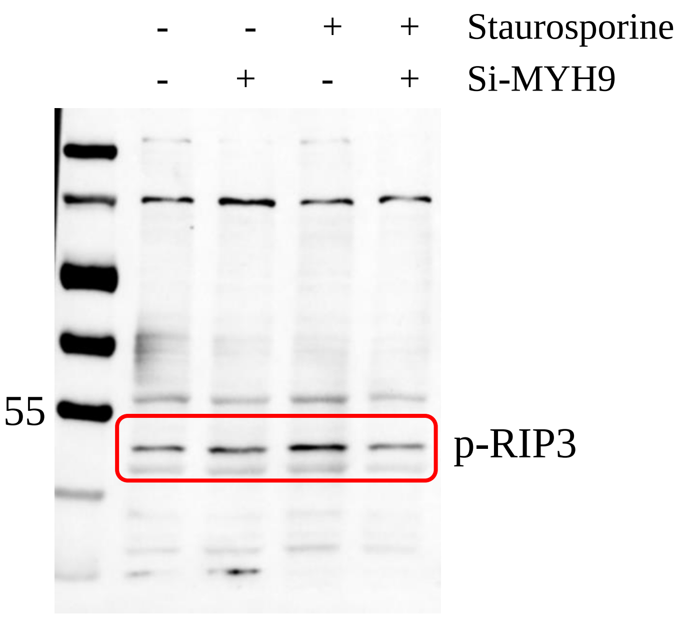

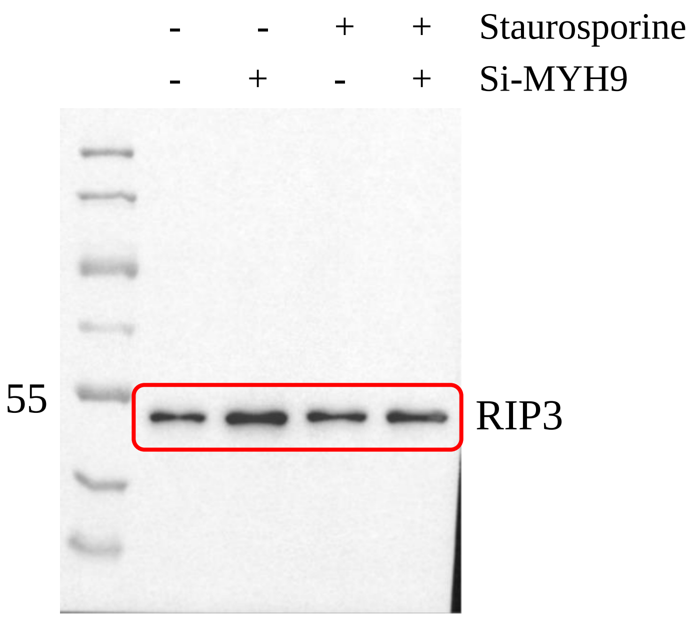

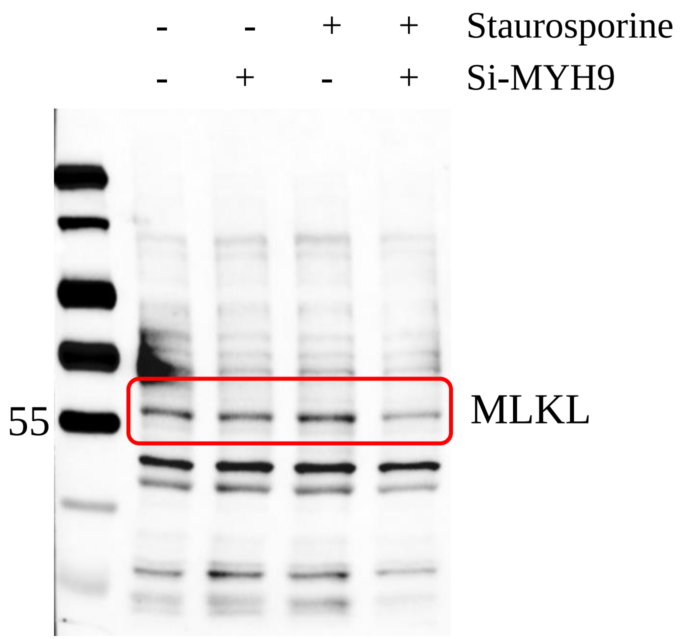

Supplementary Figure 2 G

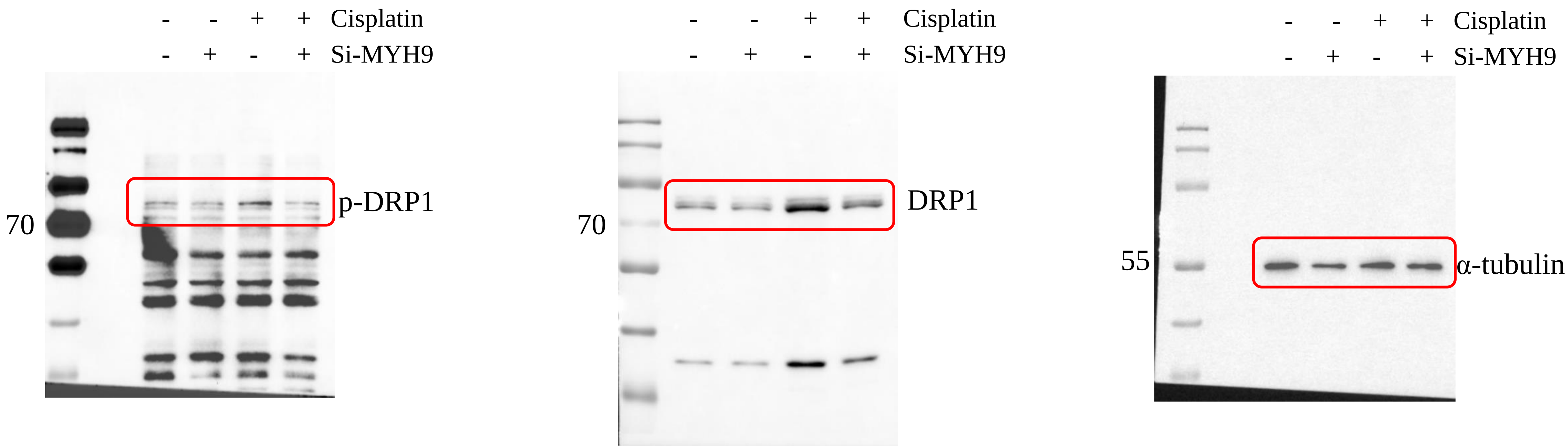

Supplementary Figure 2 J

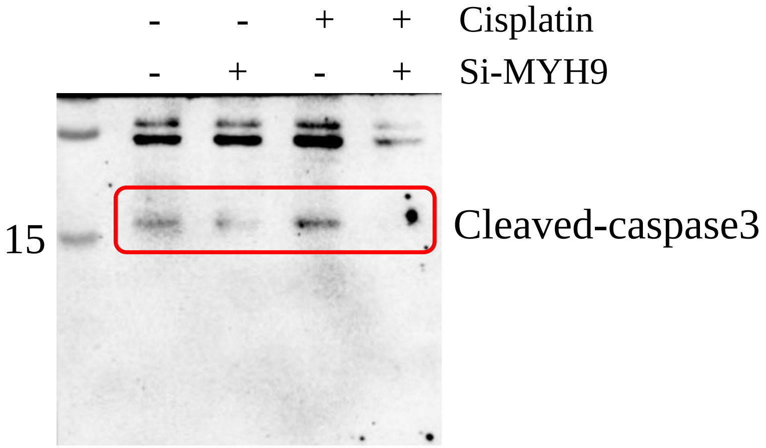

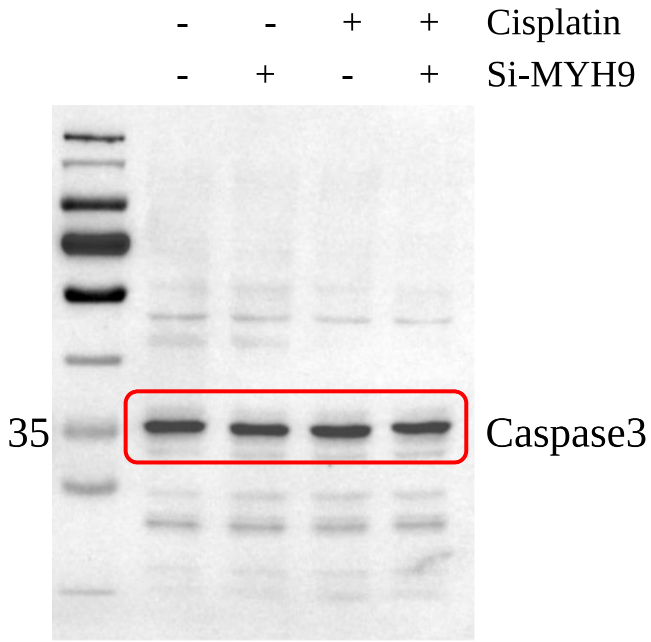

Supplementary Figure 2 J

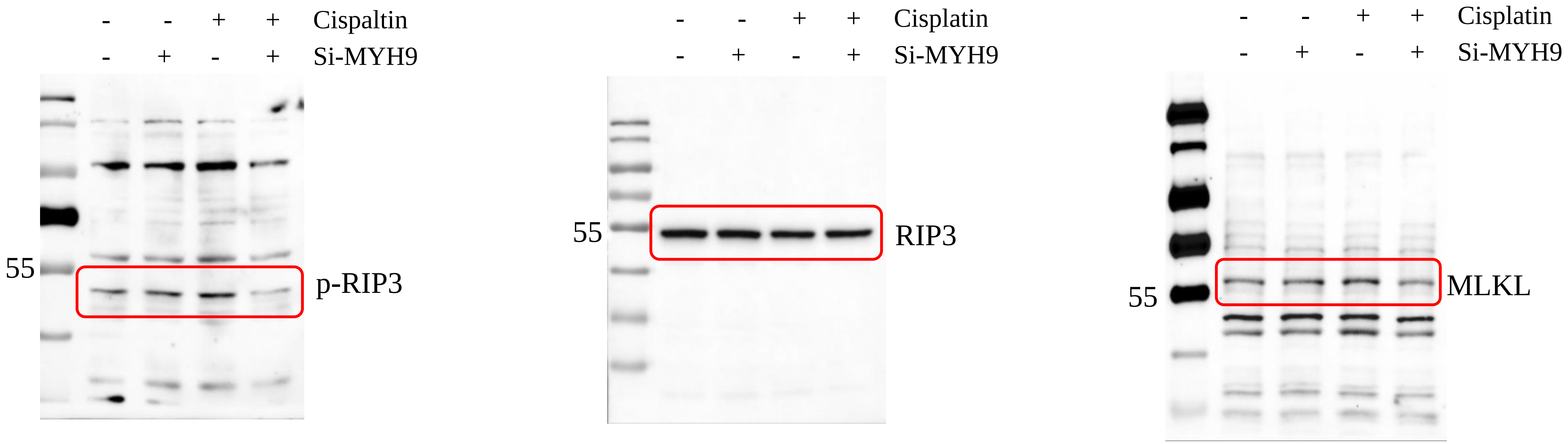

Supplementary Figure 2 J

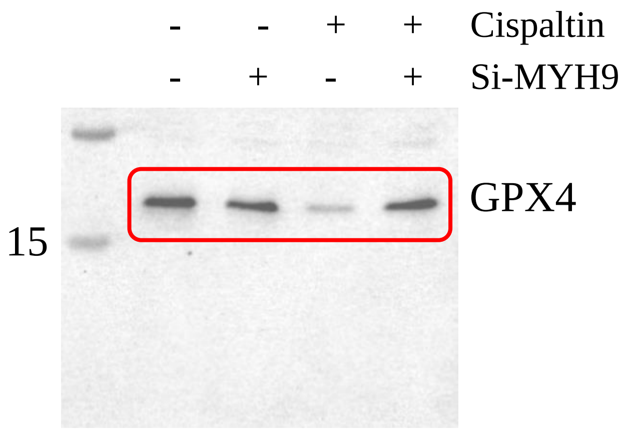

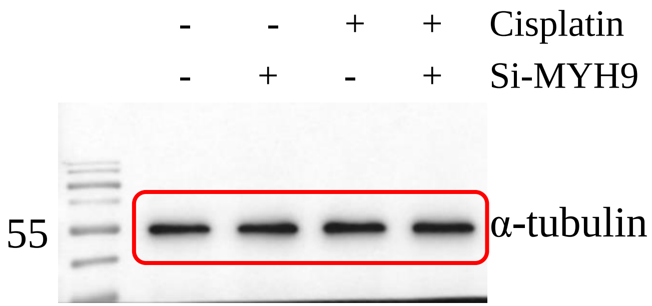

Supplementary Figure 3 A

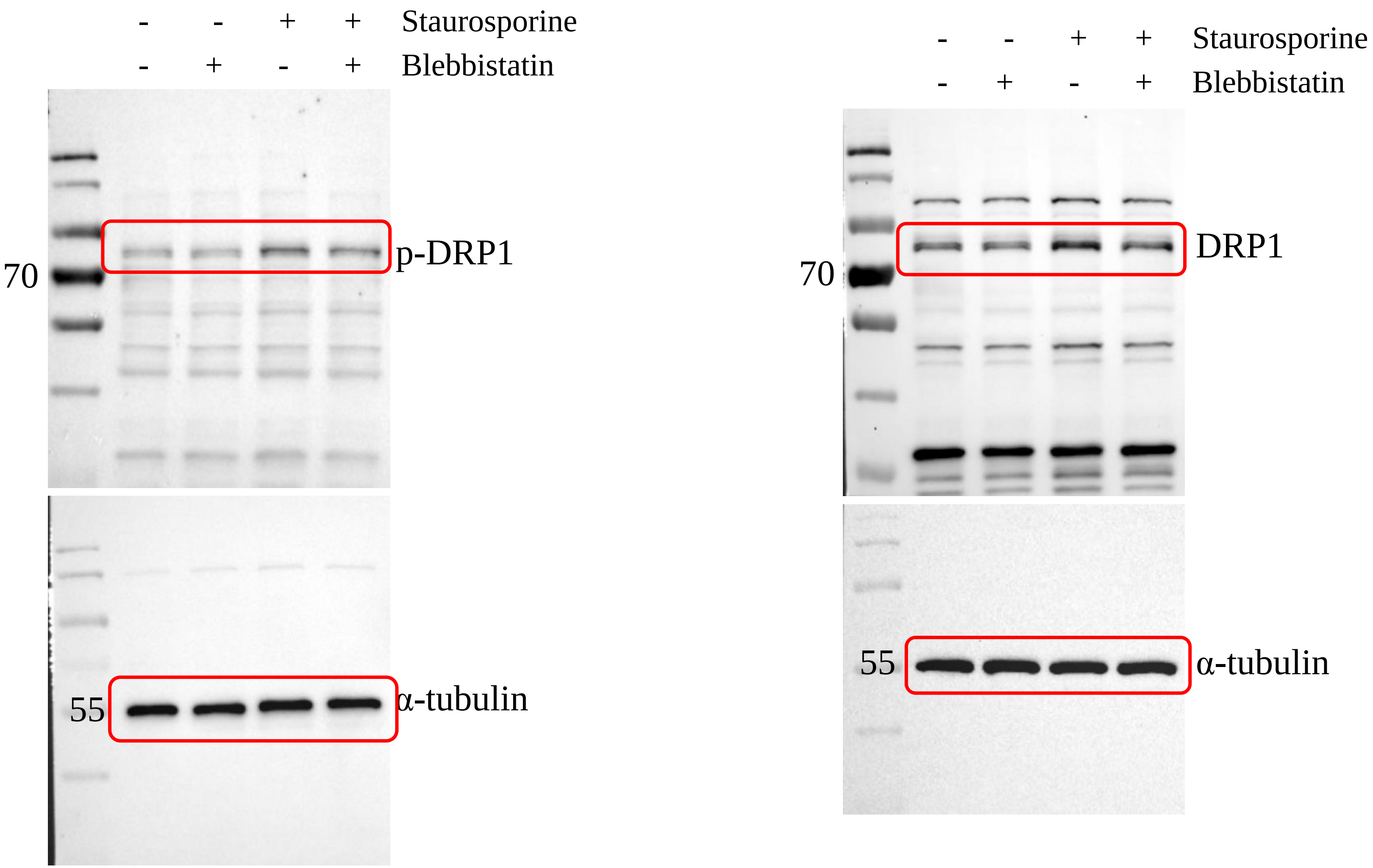

Supplementary Figure 3 D

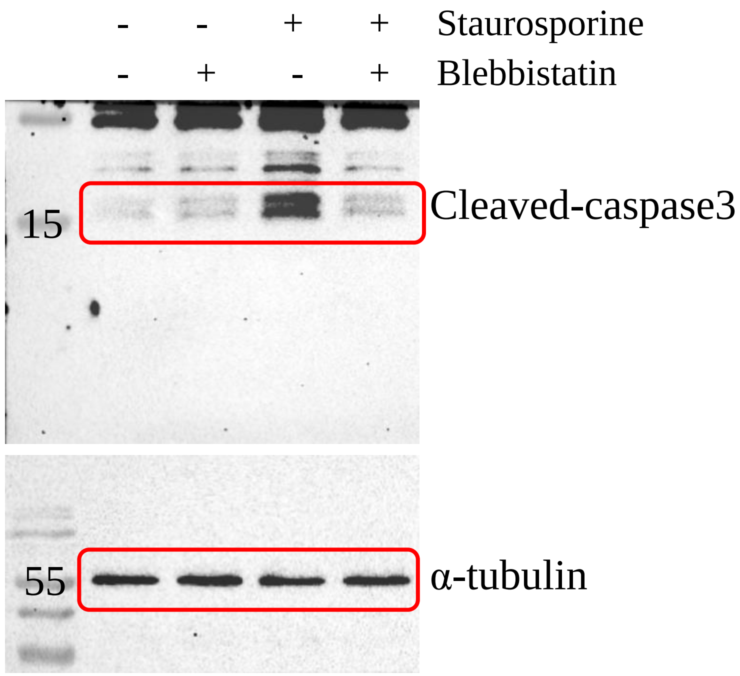

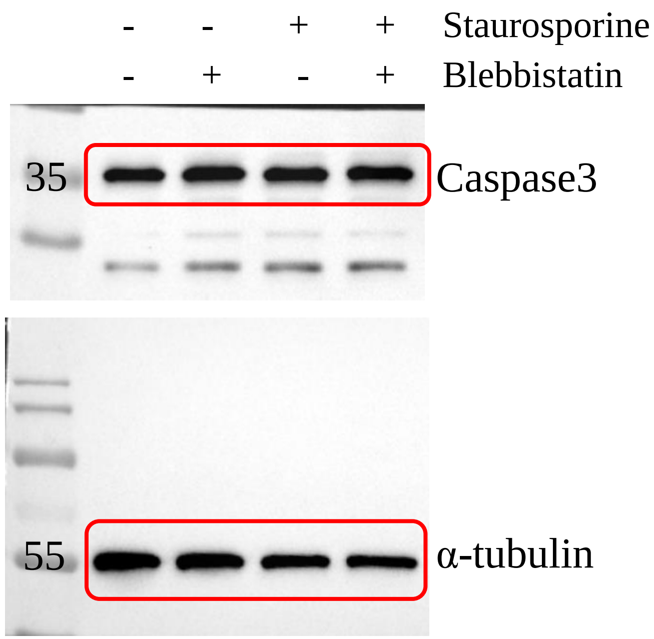

Supplementary Figure 3 D

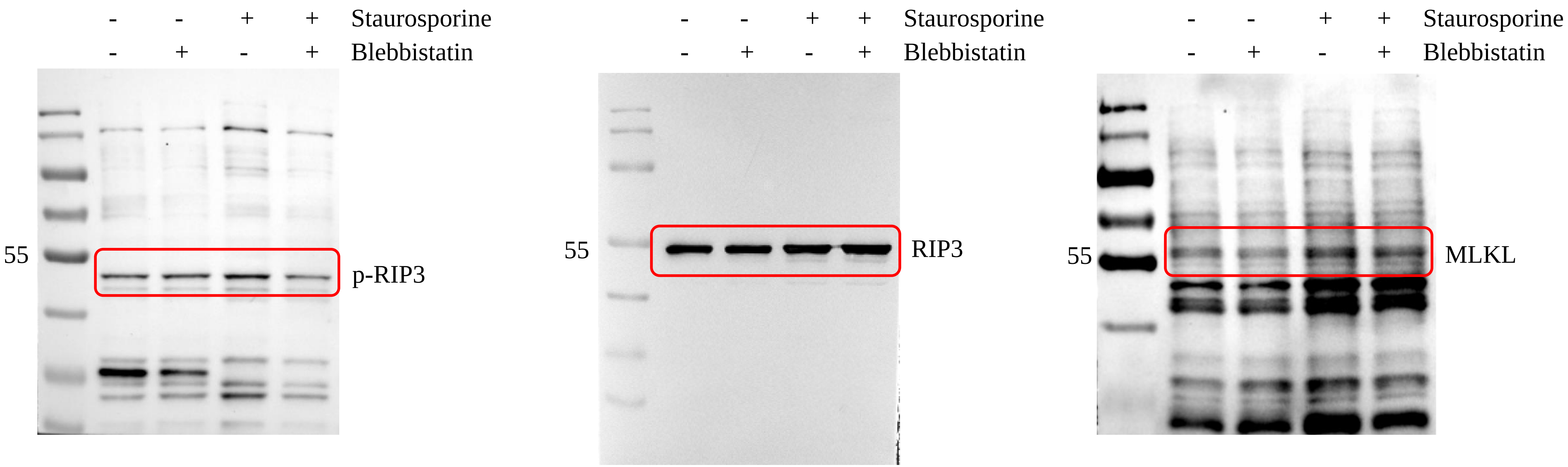

Supplementary Figure 3 G

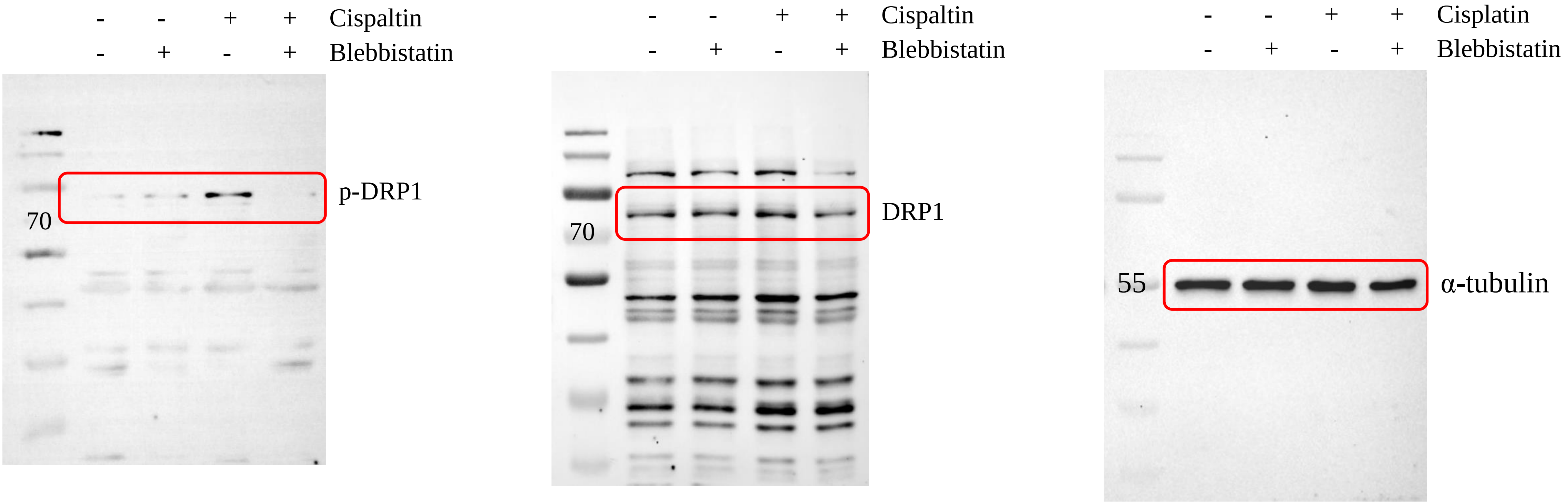

Supplementary Figure 3 J

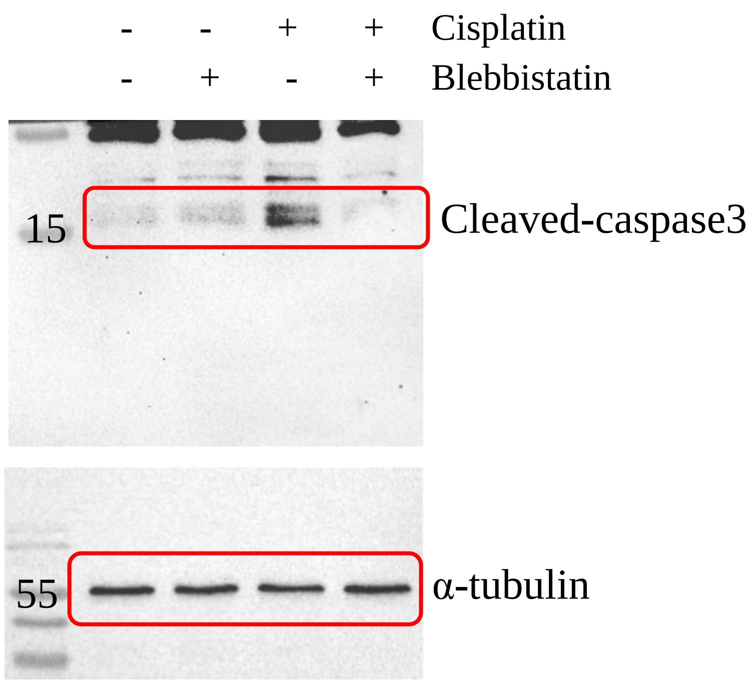

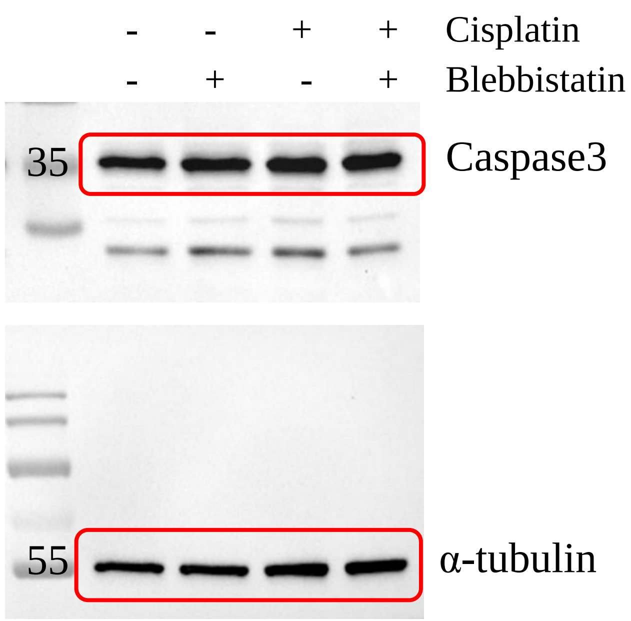

Supplementary Figure 3 J

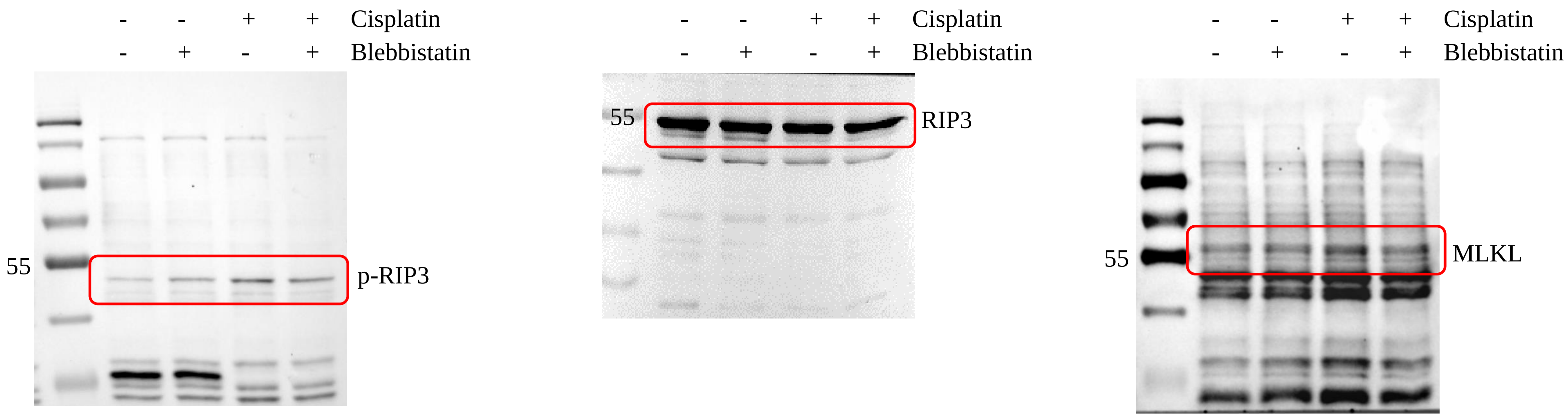

Supplementary Figure 3 J

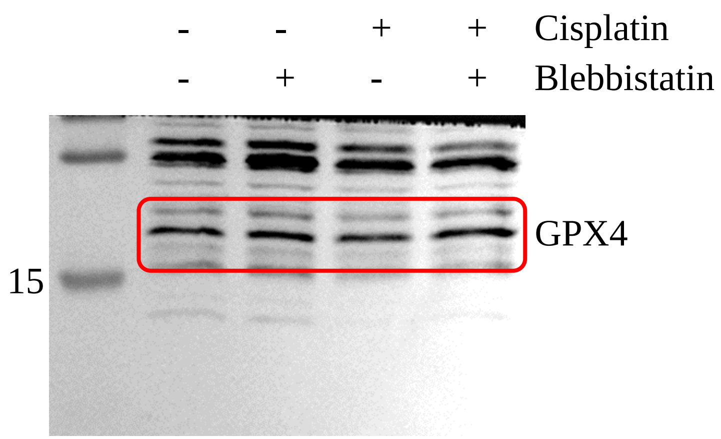

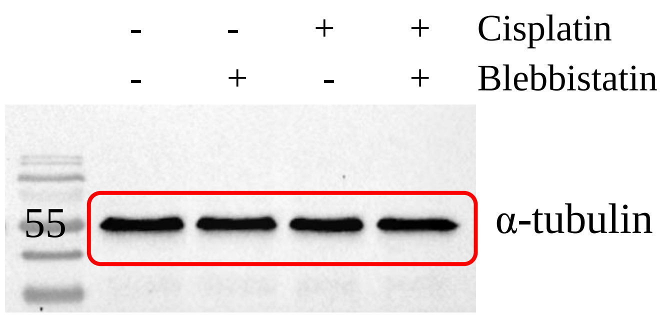

Supplementary Figure 4 A

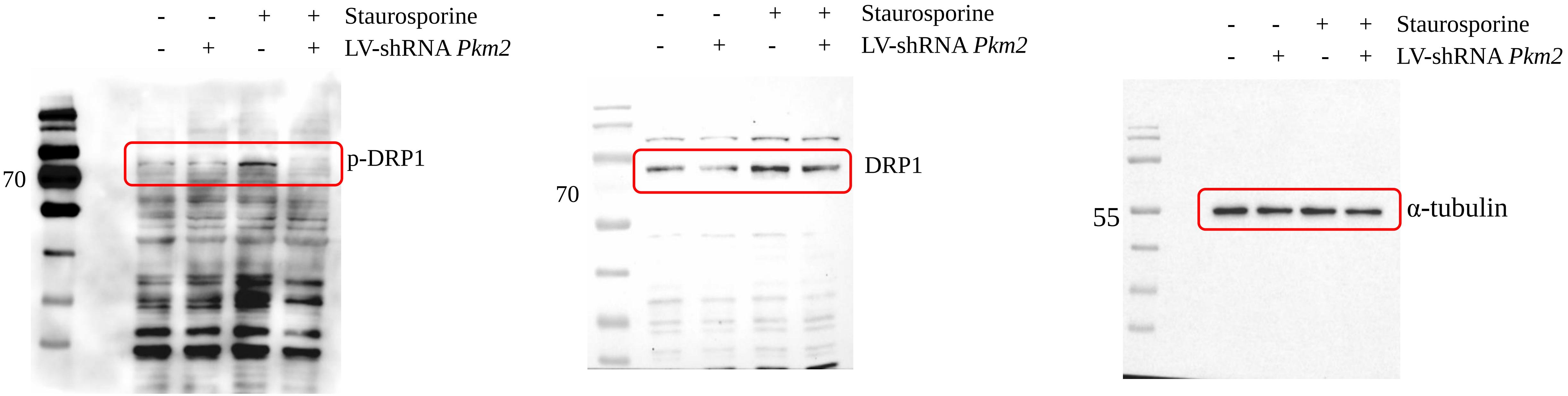

Supplementary Figure 4 B

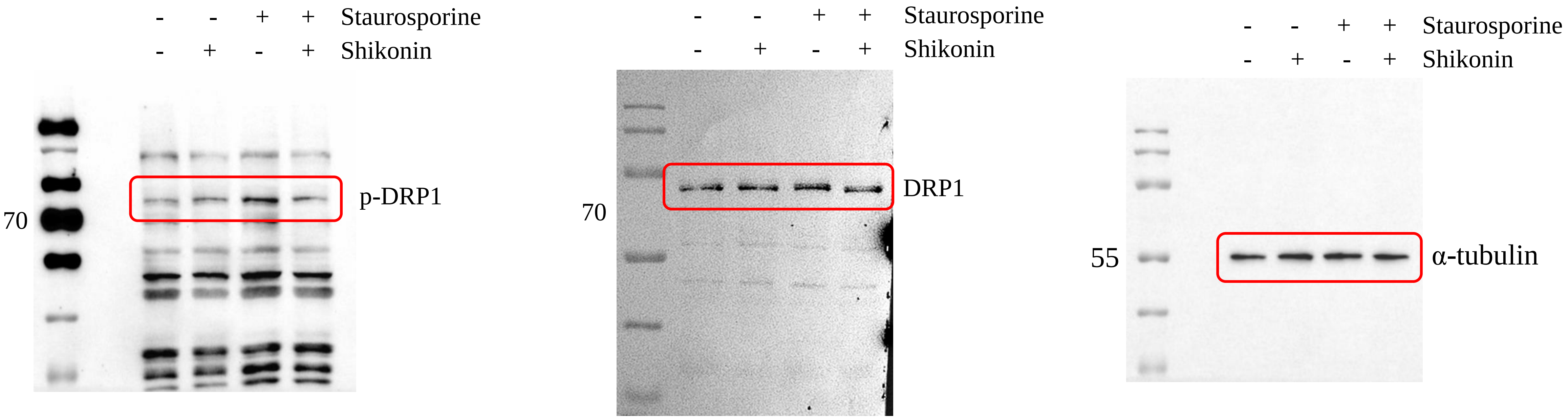

Supplementary Figure 4 C

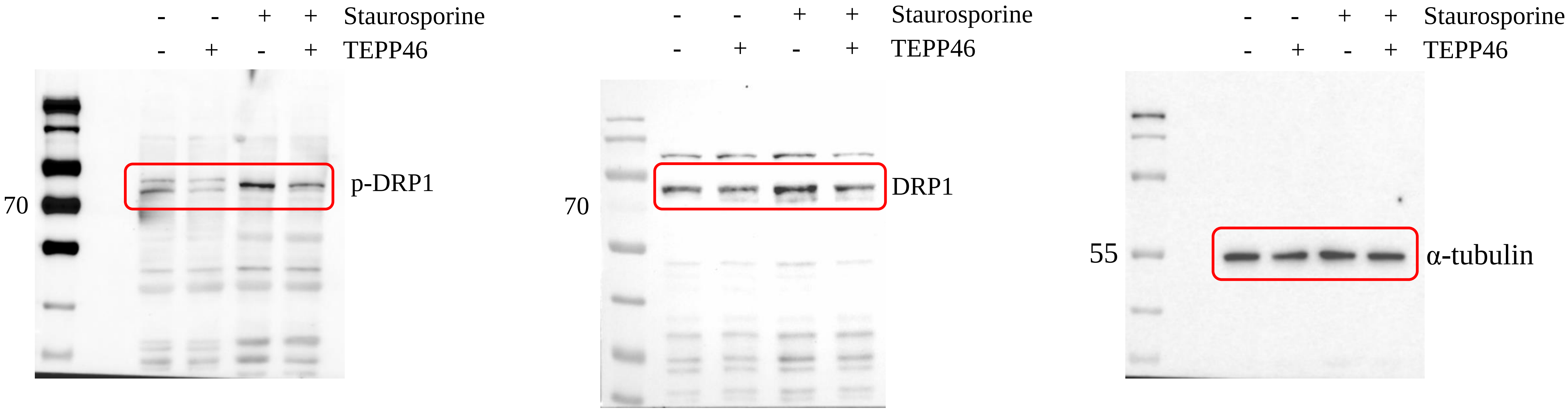

Supplementary Figure 4 D

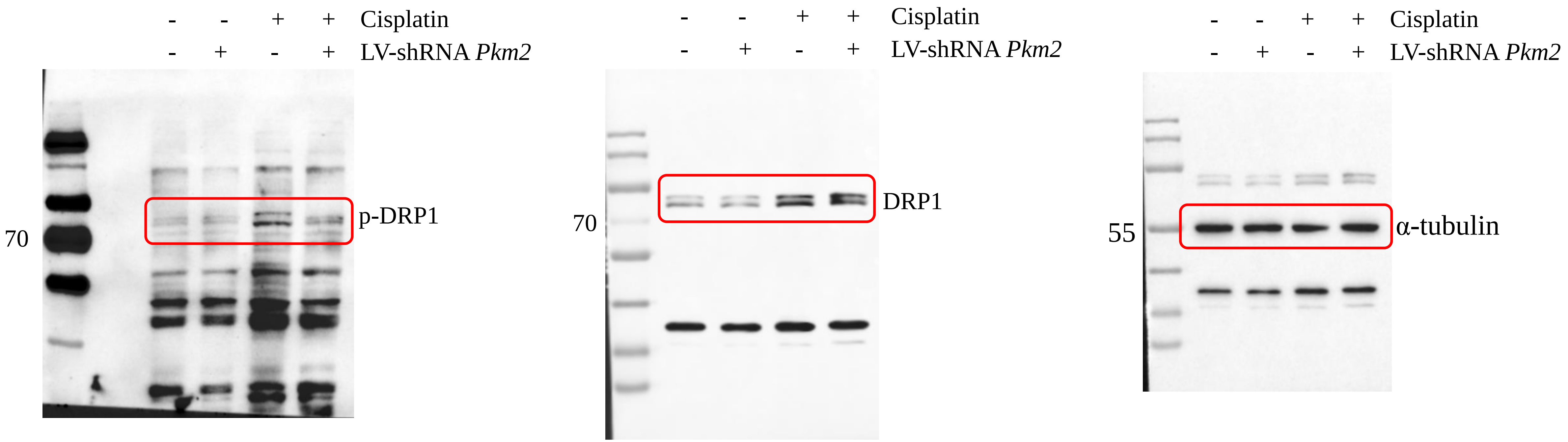

Supplementary Figure 4 E

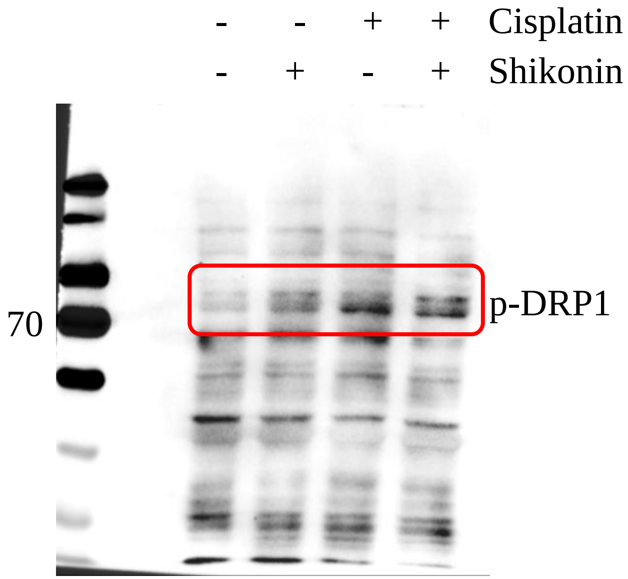

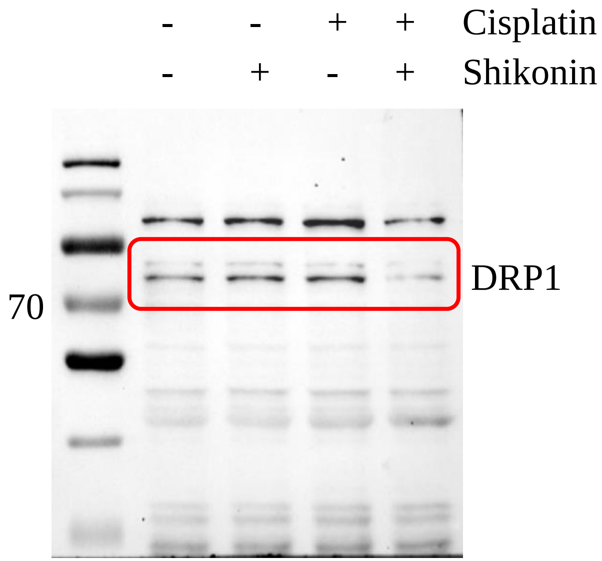

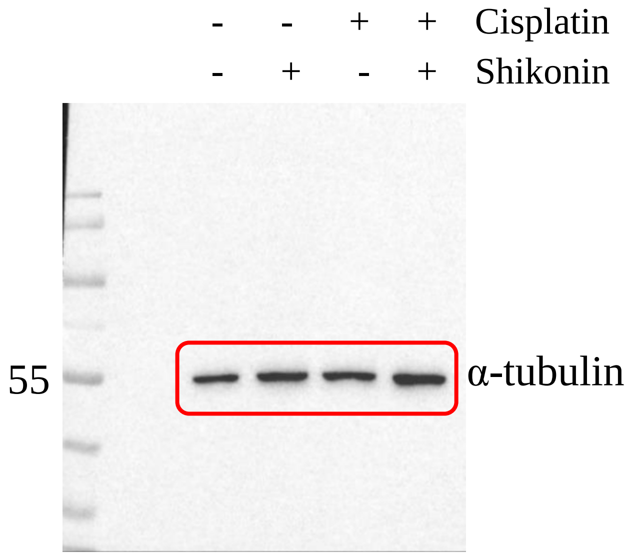

Supplementary Figure 4 F

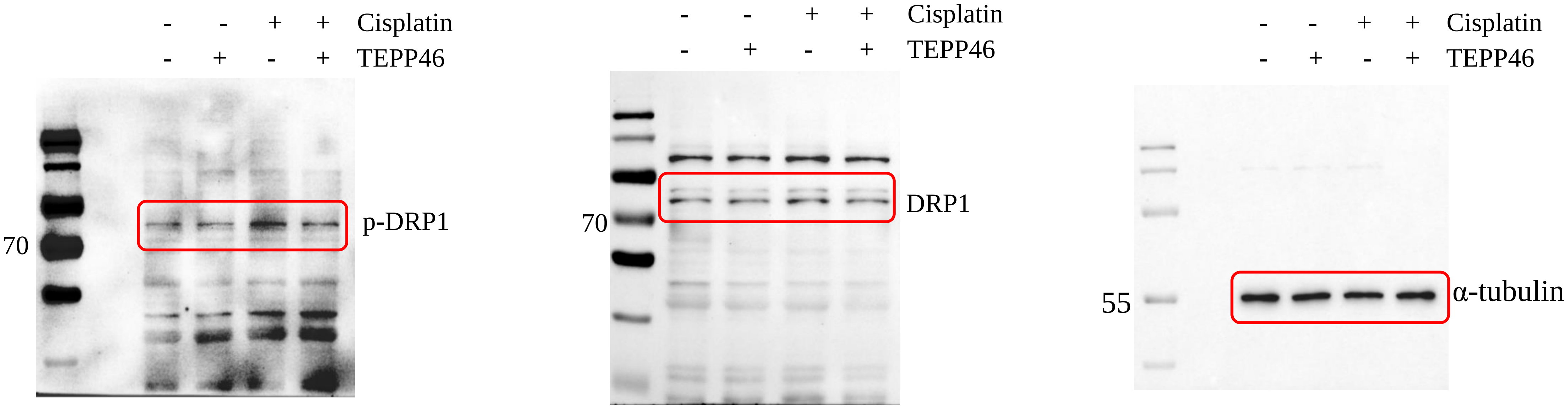

Supplementary Figure 4 G

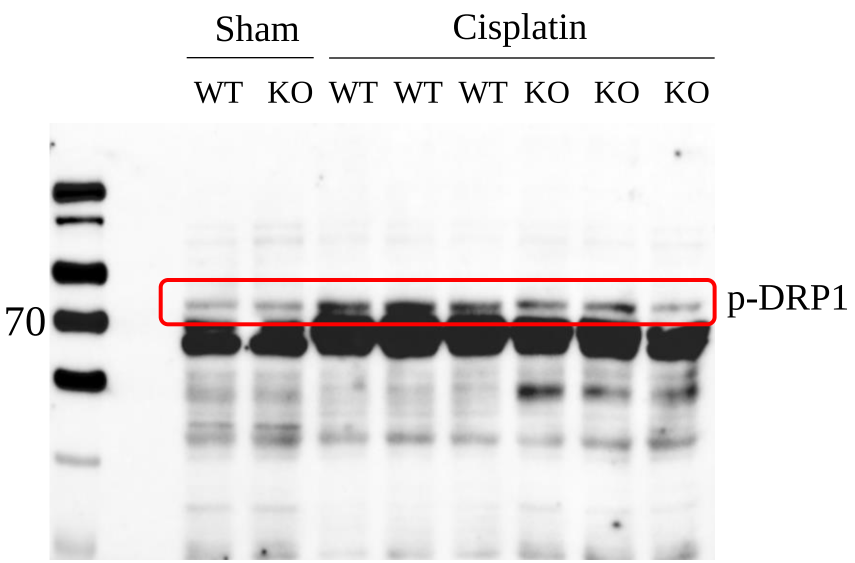

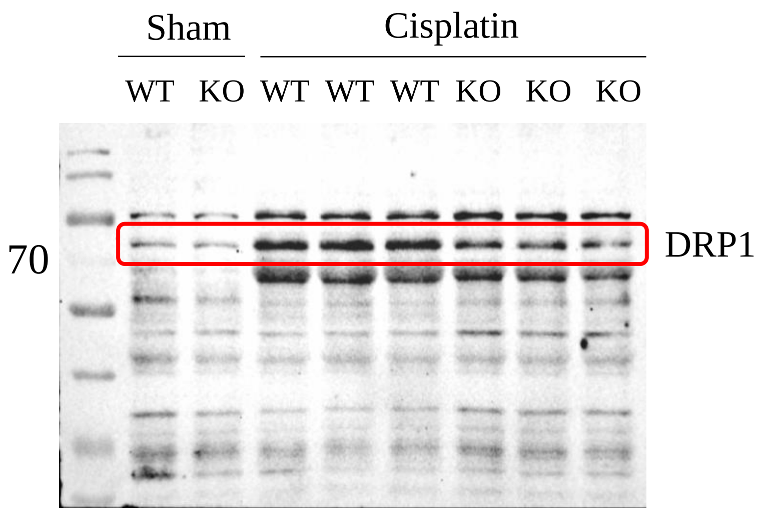

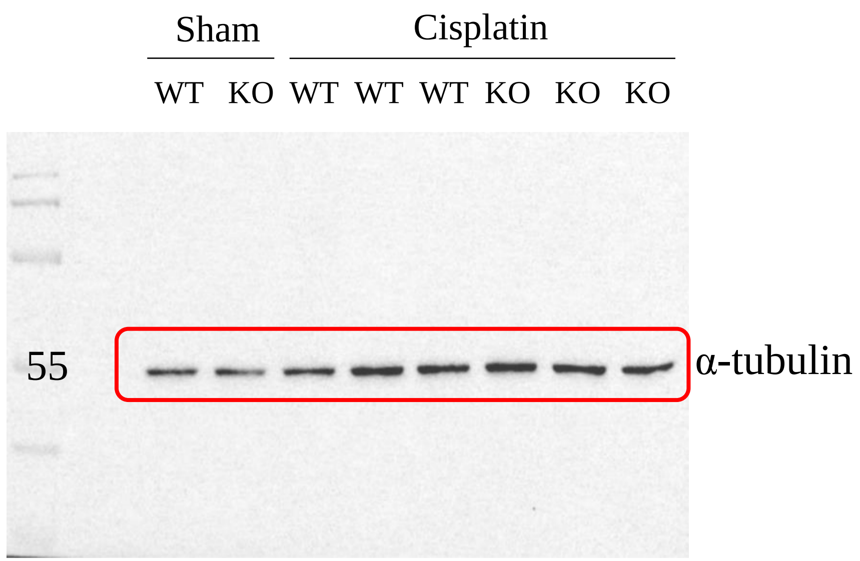

Supplementary Figure 4 H

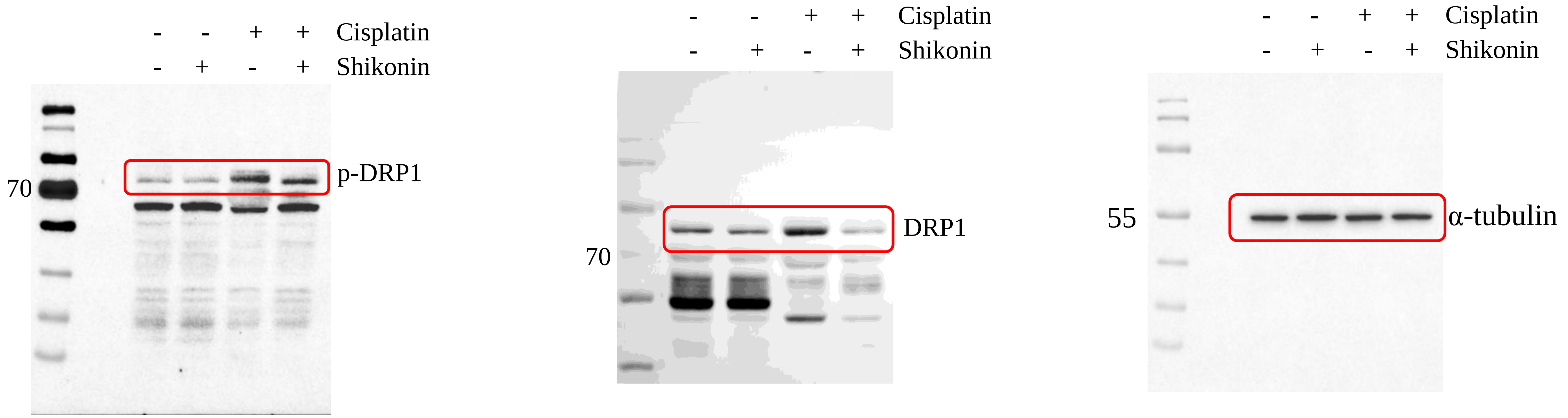

Supplementary Figure 4 I

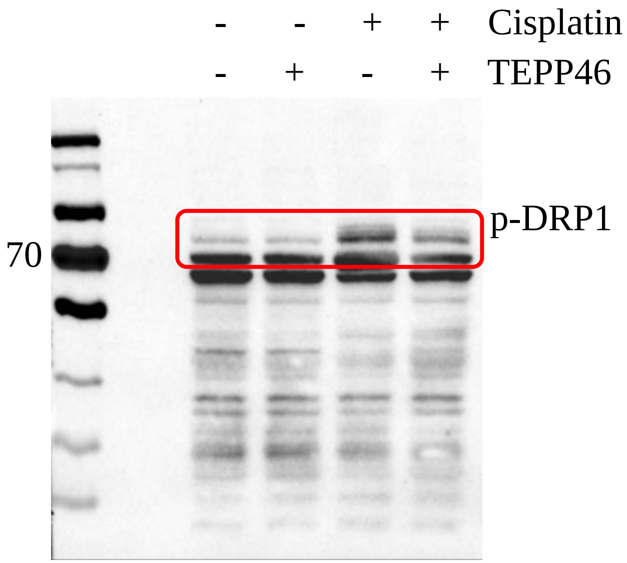

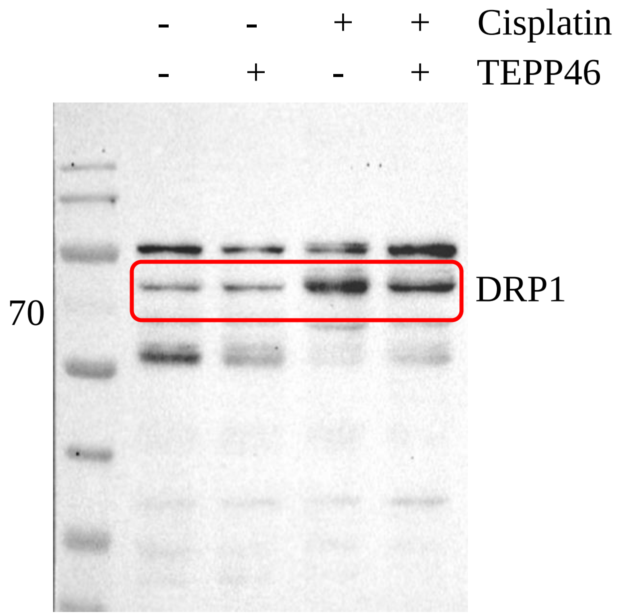

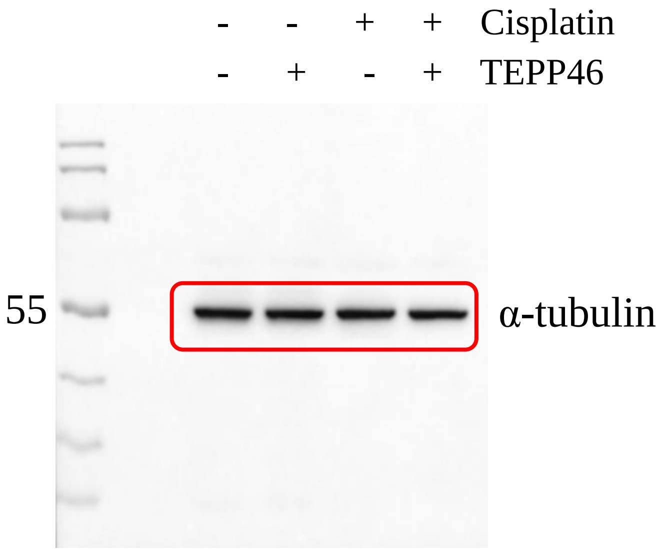

Supplementary Figure 6 C

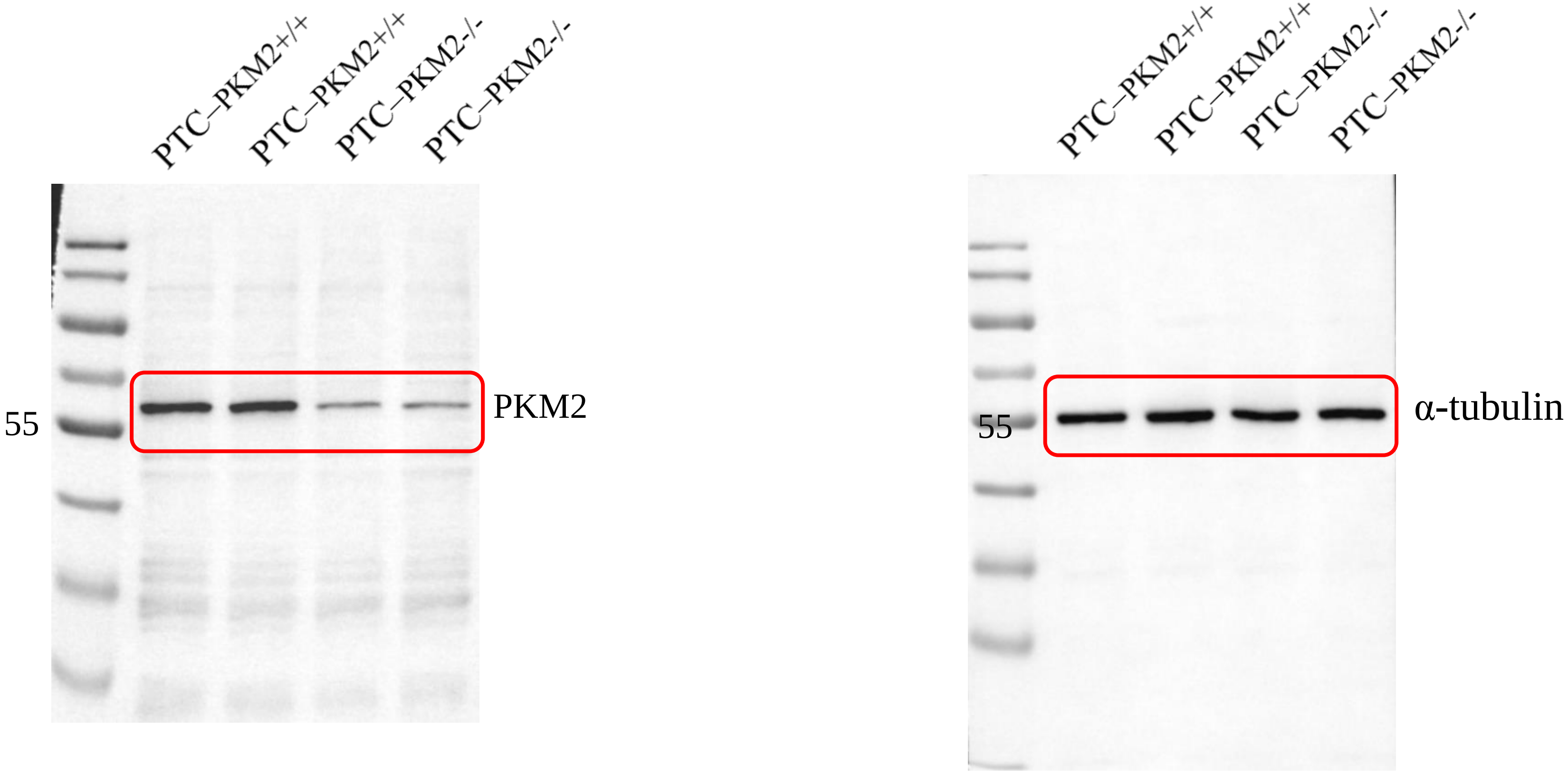

Supplement: Supplementary file 2 — original data files [file 41419_2023_6195_MOESM2_ESM.pdf]
